# Identification of Chicken Transglutaminase 1 and In Situ Localization of Transglutaminase Activity in Avian Skin and Esophagus

**DOI:** 10.3390/genes12101565

**Published:** 2021-09-30

**Authors:** Attila Placido Sachslehner, Marta Surbek, Julia Lachner, Surya Paudel, Leopold Eckhart

**Affiliations:** 1 Skin Biology Laboratory, Department of Dermatology, Medical University of Vienna, 1090 Vienna, Austria; attila.sachslehner@meduniwien.ac.at (A.P.S.); marta.surbek@meduniwien.ac.at (M.S.); julia.lachner@mduniwien.ac.at (J.L.); 2 Clinic for Poultry and Fish Medicine, Department for Farm Animals and Veterinary Public Health, University of Veterinary Medicine Vienna, 1210 Vienna, Austria; Surya.Paudel@vetmeduni.ac.at

**Keywords:** transglutaminase, cornification, keratinocytes, epidermis, esophagus, birds, chicken, evolution, enzyme activity in situ

## Abstract

Transglutaminase 1 (TGM1) is a membrane-anchored enzyme that cross-links proteins during terminal differentiation of epidermal and esophageal keratinocytes in mammals. The current genome assembly of the chicken, which is a major model for avian skin biology, does not include an annotated region corresponding to *TGM1*. To close this gap of knowledge about the genetic control of avian cornification, we analyzed RNA-sequencing reads from organotypic chicken skin and identified *TGM1* mRNA. By RT-PCR, we demonstrated that *TGM1* is expressed in the skin and esophagus of chickens. The cysteine-rich sequence motif required for palmitoylation and membrane anchorage is conserved in the chicken TGM1 protein, and differentiated chicken keratinocytes display membrane-associated transglutaminase activity. Expression of *TGM1* and prominent transglutaminase activity in the esophageal epithelium was also demonstrated in the zebra finch. Altogether, the results of this study indicate that *TGM1* is conserved among birds and suggest that chicken keratinocytes may be a useful model for the study of TGM1 in non-mammalian cornification.

## 1. Introduction

The outermost epidermal layer of amniotes (mammals, reptiles and birds) consists of multiple layers of cornified keratinocytes. Cornification is a mode of programmed cell death that involves the covalent cross-linking of proteins in the cell periphery to form a so-called cornified envelope [1,2,3]. A large portion of the cornified envelope proteins is encoded by genes of the epidermal differentiation complex (EDC) which was originally identified in mammals [4,5,6]. Later studies showed that an EDC is also present in other amniotes [7,8,9,10,11,12].

The cross-linking of epidermal proteins depends on disulfide bonds between cysteine residues and isopeptide bonds between glutamine and lysine residues [1,13]. The latter bond is formed under the control of transglutaminases (TGMs) [14]. In the human epidermis, TGM1, TGM3 and TGM5 are implicated in cornified envelope formation [15,16]. TGM1 is anchored to the cell membrane, whereas TGM3 and TGM5 are cytosolic enzymes [17]. The association of TGM1 with the membrane depends on the palmitoylation of a cysteine-rich sequence motif close to the amino-terminus of the TGM1 protein. Defects of the human TGM1 gene cause autosomal recessive lamellar ichthyosis, a debilitating and life-threatening skin disease [18], and targeted inactivation of *TGM1* in mice leads to perinatal death due to skin barrier impairment [19,20]. Apart from the skin, TGM1 is highly abundant in the esophagus, where its expression coincides with that of the EDC gene *Cornulin (CRNN)* [21,22]. Esophageal keratinocytes form cornified envelopes in vitro [23], but the functions of TGM1 and transglutamination in the esophagus are not fully understood at present.

In addition to *TGM1* in mammals, *TGM1* orthologs were reported to be conserved in alligators, Xenopus frogs and fishes [24,25]. The existence of *TGM1* in fish suggests that *TGM1* originated prior to the evolution of a continuous cornified layer on the skin surface, which first appeared in amphibians. Surprisingly, there is no annotated region corresponding to *TGM1* in the current release of the chicken (*Gallus gallus*) genome sequence (assembly: GCF_016699485.2), raising the question as to whether epidermal transglutamination might occur by an atypical mechanism in the main avian model species [26,27]. The uncertainty about the conservation of a central enzyme of epidermal transglutamination is worrying, because most of the research on avian epidermal cornification proteins was conducted in the chicken [7,8,28,29,30,31,32,33,34,35,36,37]. Recently, transglutamination of the EDC protein cornulin was proposed to influence the mechanical properties of the avian esophagus [38]. According to this hypothesis, loss of cornulin in the avian clade Passeri has led to higher elasticity of the oroesophageal cavity, a feature supporting pure-tone song in songbirds [38]. However, the enzymatic process of transglutamination has not yet been reported for the esophagus of birds.

In view of the important role of the chicken as a model for non-mammalian skin biology, we aimed at determining whether the chicken has a homolog of mammalian *TGM1* and whether chicken skin and esophagus show a pattern of TGM activity similar to that in the homologous organs of mammals.

## 2. Materials and Methods

### 2.1. Animals

Tissue samples were prepared from commercial broiler chickens (21 days old; Ross-308), specified pathogen-free embryos (18 days of incubation, corresponding to Hamburger and Hamilton stage 44 [39]; VALO Biomedica, Sachsenring, Germany) and zebra finches and mice (strain C57BL/6J) immediately after killing the animals. The Ethics Committee of the Medical University of Vienna decided that, in agreement with the national laws, a permission for sacrificing animals (zebra finches and mice) for organ preparation was not required. For sampling in chickens, non-treated negative control birds from an animal trial that was performed with different objectives at the University of Veterinary Medicine Vienna were utilized during necropsy. The trial was approved by the institutional ethics and animal welfare committee and the national authority according to §§ 26ff. of Animal Experiments Act, Tierversuchsgesetz 2012–TVG 2012 (license number BMBWF GZ: 2020-0.761.569).

### 2.2. Transglutaminase In Situ Activity Assay

Fresh tissue samples were dissected and washed in phosphate-buffered saline (PBS). Chicken in vitro skin models were obtained from a previous study [40]. The samples were placed in cryomolds (Tissue-Tek^®^ Cryomold^®^, Sakura FineTec, Torrance, CA, USA) filled with optimal cutting temperature (OCT) compound (Scigen, Paramount, Canada) and snap-frozen in liquid nitrogen. Cryo-sections of 6 µm thickness were prepared with a cryostat (Leica CM3050S) and stored at −20 °C or stained immediately. An in situ transglutaminase activity assay was performed based on published protocols [41,42,43]. Briefly, cryosections were thawed at room temperature for 10 min before they were placed in PBS for 5 min at room temperature to remove remaining OCT from the slides. The sections were encircled with a liquid blocker (Daido Sangyo Co. Ltd. Tokyo, Japan) and subsequently incubated with 1% bovine serum albumin (BSA) in 1 M Tris-HCl, pH 7.4 at room temperature for 30 min. Subsequently, the samples were incubated with 5 µM Alexa-fluor 555-cadaverine (catalog number A30677, Thermo Fisher Scientific, Waltham, MA, USA) as TGM substrate in 0.1 M Tris-HCl pH 7.4, and either 5 mM CaCl_2_ to facilitate transglutaminase activity or 5 mM EDTA to suppress transglutaminase activity (negative control) for 2 h at room temperature under protection from light. The reaction was stopped by incubating the sections in 25 mM EDTA in PBS for 5 min. The sections were rinsed in PBS and then incubated with 1 µg/mL nucleic acid stain Hoechst 33258 (Molecular Probes, Eugene, OR, USA) at room temperature protected from light for 5 min. Afterwards, the sections were rinsed in PBS and mounted with Permafluor (catalog number TA-030-FM, Thermo Fisher Scientific, Waltham, MA, USA). Commercially available nail polish was used to seal the slides to prevent the sections from drying. Sections were studied with an Olympus BX63 light microscope and images were taken with an Olympus UC-90 camera. Fluorescent images were taken and merged with cellSens Dimensions (version 1.16). Confocal images were taken with a Zeiss 980 confocal laser scanning microscope using the program Zen (Blue edition). Where indicated in the figure legends, the brightness of images of both activity labeling and negative controls was equally increased.

### 2.3. RNA Preparation and RT-PCR

Tissue samples were placed in RNA-later (AM7020, Invitrogen, Carlsbad, CA, USA) immediately after dissection and stored overnight at 4 °C, and subsequently at −80 °C for a long-term duration. RNA isolation was performed with Trizol (catalog number 30-2010 PEQ-GOLD Trifast, VWR, Radnor, PA, USA) from homogenized tissues according to published protocols [44]. cDNA synthesis was performed with the qScript^®^ cDNA synthesis kit (catalog number 95047-025, Quantabio, Beverly, MA, USA). Chicken TGM1 was amplified with the intron-spanning primers 5′-CTGTCCTGCCCCGCATCCATC-3′ and 5′-GAGCTGCAATTCGACGCCTC-3′. Chicken TGM2 was amplified with the primers 5′-TCTGTGTATCGCTCTGCTCC-3′ and 5′-TGGGCTCCAGGGACTACATA-3′. Zebra finch TGM1 was amplified with the primer pair 5’-GTGCAGGTCGTGTTCCAGAA-3′ and 5’-CCAATCAGTGACGCCGCTC-3’. cDNA of the housekeeping gene EEF1A1 was amplified with the primer pairs 5′-GGCCCGAAGTTCCTGAAATC-3′ and 5′-CTGTTGGTGTCATCAAGGCC-3′ for chicken and 5′-AGATGGCCCCAAATTCCTGA-3’ and 5’-GTCGCTGTTGGTGTCATCAA-3′ for zebra finch. Primers were synthesized by Microsynth (Switzerland). cDNA was PCR-amplified with the Dream Taq DNA polymerase (catalog number EP0702, Thermo Scientific, Waltham, MA) according to a published protocol [45] which was modified by the addition of 5% DMSO (Merck, Darmstadt, Germany) to the reaction buffer.

### 2.4. Chicken TGM1 Sequence Assembly In Silico

Publicly available TGM1 protein sequences of human (GenBank accession number: NP_000350.1), zebra finch (XP_041568788) and Swainson’s thrush (XP_032940238) were used as queries for tBLASTn searches [46] in sequence reads of a chicken in vitro skin model [40] (NCBI accession number: SRX9610284). The reads were translated with the Expasy Translate tool at https://web.expasy.org/translate/ (accessed on 28 September 2021). The 3’-terminal end of the chicken TGM1 sequence was identified by tBLASTn in the whole genome shotgun sequence of the chicken (NCBI accession: JAENSL010000336). Overlapping sequence reads and exons of chicken TGM1 were assembled to obtain the complete coding sequence of chicken TGM1.

### 2.5. Molecular Phylogenetics 

Transglutaminase sequences for the phylogenetic analysis were downloaded from NCBI GenBank (Table S1). The sequences were aligned using MAFFT (Version 7.427) [47] with the parameters --maxiterate set to 1000 and --localpair. Alignments were trimmed using BMGE, version 1.12 [48], by setting the entropy-like value of the BLOSUM matrix to -BLOSUM30, the maximum entropy threshold (-h) to 1, and the minimum length of selected regions (-b) to 1. The first three amino-terminal amino acid residues were excluded manually in AliView [49]. The model for amino acid replacement was calculated using ProtTest (Version 3.0) [50,51]. All available matrices (-all-matrices) and models with rate variation among sites (-all-distributions) were included. The likelihood of the predicted models was assessed with the Akaike information criterion (-sort A) [52]. The selected amino acid substitution model was LG [53]. Maximum likelihood tree and bootstrap analysis (-b 100) were performed using PHYML (Version 20120412) [54]. Tree topology (t), branch length (l), and rate parameters (r) were optimized (-o tlr). Visualization and annotation of phylogenetic trees was performed with FigTree (http://tree.bio.ed.ac.uk/software/figtree/, accessed on 28 September 2021).

## 3. Results

### 3.1. Identification of Chicken TGM1

**Figure 1:**
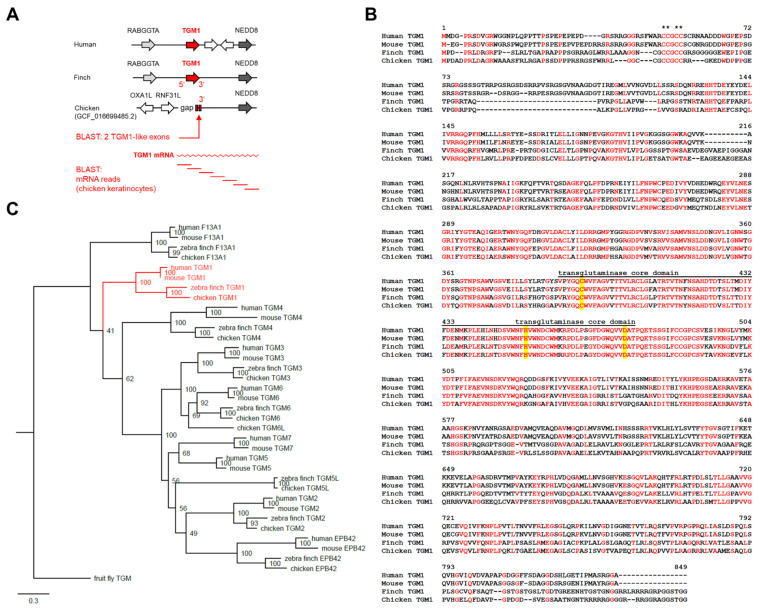
Identification and sequence of chicken *TGM1*. (**A**) Gene locus of *TGM1* in human, mouse, zebra finch and chicken. Synteny is indicated by the conserved genes *TINF2* and *NEDD8* (grey). Two *TGM1*-like exons were identified at the position indicated by the arrow. Genes are indicated by arrows pointing in the direction of transcription. BLAST search for RNA-sequencing reads covering the 5’ regions of chicken *TGM1* is schematically shown at the bottom of the panel. (**B**) Alignment of amino acid sequences of TGM1 proteins from human, mouse, zebra finch and chicken. Identical residues in all sequences are shown with red fonts. Asterisks indicate the cysteine residues at the palmitoylation site. The transglutaminase core domain [55] is marked by a line above the sequences. Residues of the catalytic triad are highlighted by yellow shading. (**C**) Phylogenetic analysis of TGMs from human, mouse, zebra finch and chicken.

To determine whether *TGM1* is conserved in the chicken, we searched the genome sequence assembly of the chicken for a chromosomal segment homologous to the locus of *TGM1* in other species. In the human and zebra finch (*Taeniopygia guttata*), *TGM1* is located between *RABGGTA* and *NEDD8* (Figure 1A). *RABGGTA* is not present in the current *Gallus gallus* genome annotation release (assembly: GCF_016699485.2), whereas *NEDD8* is located on chromosome 35. On the 5′-side of the *NEDD8* gene, i.e., the expected locus of the *TGM1* gene, there is a sequence gap in the assembly, and the genes predicted on the other side of the gap are not homologous to genes flanking *TGM1* in other species (Figure 1A).

tBLASTn searches using the amino acid sequence of zebra finch TGM1 protein (XP_041568788) as the query revealed the presence of two sequence stretches similar to the last two exons of *TGM1* in the chicken genome region between *NEDD8* and the genome sequence gap mentioned above (Figure 1A). Next, we performed tBLASTn searches in the RNA-sequence reads that we had previously obtained in a transcriptome analysis of chicken epidermis reconstituted in vitro [40]. Using this approach, we obtained a series of overlapping short reads which, together with the sequence of the *TGM1*-like exons, besides *NEDD8*, covered the entire coding sequence of chicken *TGM1* (Figure 1A and Figure S1, Table S2).

Translation of the predicted chicken *TGM1* mRNA sequence (Figure S1) and alignment of the resulting amino acid sequence with TGM1 of human, mouse and zebra finch showed a high degree of sequence conservation (Figure 1B). The palmitoylation sequence motif (Figure 1B, asterisks) and the amino acid residues (cysteine, histidine, aspartate) representing the catalytic triad (Figure 1B, yellow shading) within the transglutaminase core domain are conserved in chicken TGM1. Molecular phylogenetics confirmed that chicken TGM1 is orthologous to TGM1 of human, mouse and zebra finch (Figure 1C and Figure S2).

### 3.2. TGM1 Is Expressed in the Epidermis and Esophagus of the Chicken

**Figure 2:**
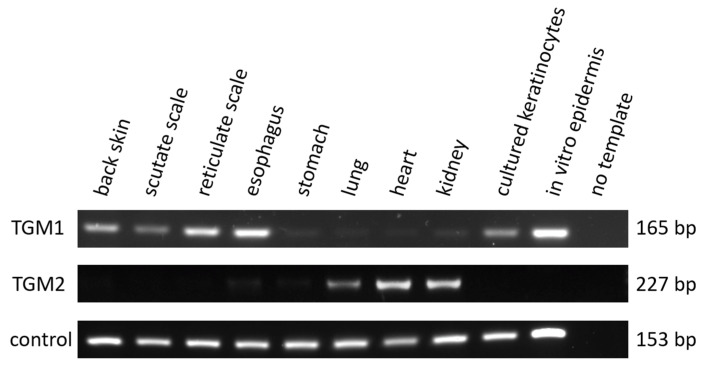
Expression analysis of *TGM1* and *TGM2* in chicken tissues and cells. RNA from the indicated tissues and cultured cells was subjected to RT-PCR with primers specific for *TGM1*, *TGM2*, and the house-keeping gene *EEF1A1* (control). PCR products were analyzed by agarose gel electrophoresis, revealing bands that correspond to the predicted size of the PCR products.

Using primers annealing to *TGM1*-specific sequences, we performed RT-PCR screening of chicken tissues and isolated cells. *TGM1* mRNA was detected in the skin from the back, scutate scales on the legs, reticulate scales from the toes and in the esophagus, but not or only at very low levels in the stomach, lung, heart and kidney of chickens (Figure 2). *TGM1* is also expressed in isolated keratinocytes and in the epidermal compartment of chicken skin reconstituted in vitro [40] (Figure 2). By contrast, *TGM2* was detected only in lung, heart and kidney (Figure 2). Transcripts of the house-keeping gene *EEF1A1* were detected in all samples. TGM1 expression was not only detected in adult chicken skin and esophagus tissues, but also in the tissues sampled from embryos at Hamburger and Hamilton stage 44 (Figure S3). RT-PCR analysis of zebra finch tissues demonstrated conserved expression of *TGM1* in the skin and esophagus (Figure S4).

### 3.3. TGM Activity Is Present in Differentiated Epithelial Cells of Chicken Skin and Esophagus

**Figure 3:**
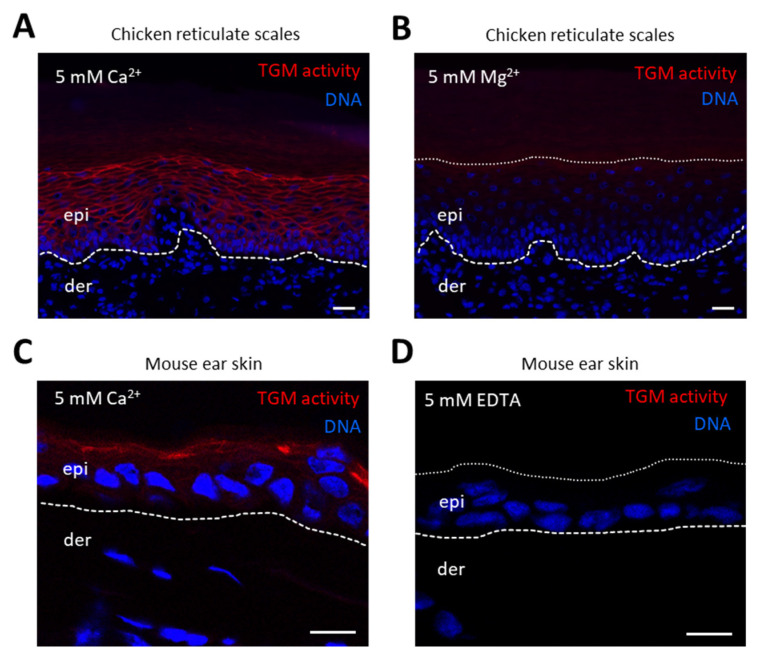
In situ labeling of transglutaminase (TGM) activity in chicken reticulate scales and mouse skin. Reticulate scales from an adult chicken (**A**,**B**) and mouse ear skin (**C**,**D**) were cryosectioned and incubated with Alexa-fluor 555-cadaverine (red) in buffer containing either calcium (**A**,**C**), magnesium (**B**) or EDTA (**D**). Nuclear DNA was labeled with Hoechst 33258 (blue). The images were recorded with an Olympus BX63 microscope (**A**,**B**) and a Zeiss 980 confocal laser scanning microscope (**C**,**D**). White dashed lines indicate the lower border of the epidermis (**A**–**D**) and white dotted lines indicate the upper border of the epidermis in images of samples incubated with magnesium (**B**) and EDTA (**D**). Scale bars = 20 µm (**A**,**B**), 10 µm (**C**,**D**).

To determine if *TGM1* expression correlated with TGM activity, we determined tissue TGM activity in situ. Cryosections were incubated with the TGM substrate cadaverine coupled with a fluorescent label, and calcium, which is required for the catalytic activity of TGMs. TGM activity was readily detected in chicken reticulate scales when sections were labeled in the presence of 5 mM calcium ions (Figure 3A), whereas sections incubated in the presence of the same concentration of magnesium ions did not show a signal (Figure 3B). TGM was also detected in the scutate scales and back skin of adult chickens. Mouse skin, which served as positive control, was labeled in the uppermost layers of the epidermis under the stratum corneum (Figure 3C). Replacement of calcium ions with EDTA, which is a standard negative control in TGM activity labeling experiments [41,42,43], abolished the fluorescent labeling (Figure 3B). These experiments demonstrated calcium-dependent TGM activity in chicken skin and confirmed the specificity of the TGM activity assay.

**Figure 4:**
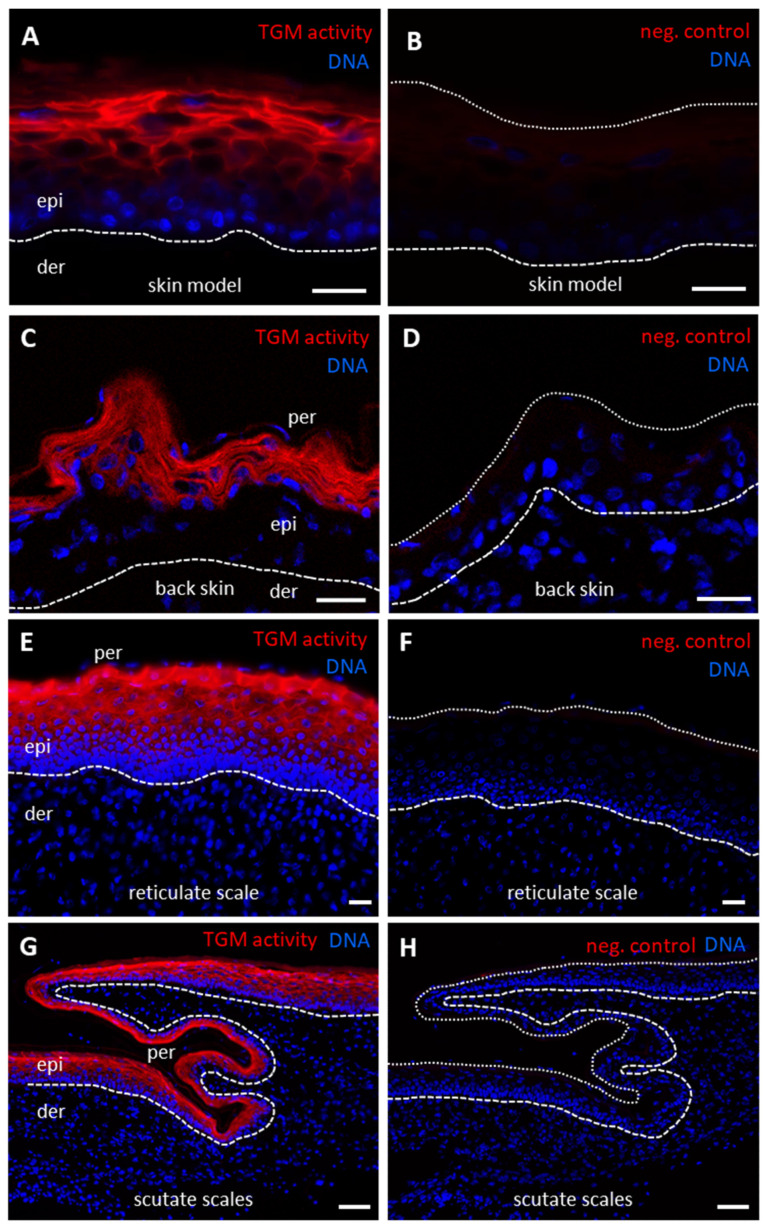
In situ labeling of transglutaminase (TGM) activity in chicken skin models and chicken skin. Skin models consisting of keratinocytes and fibroblasts isolated from chicks and cultured in vitro (**A**,**B**) and back skin (**C**,**D**), reticulate scales (**E**,**F**) and scutate scales (**G**,**H**) from chicken embryos (Hamburger and Hamilton stage 44) were cryosectioned and incubated with Alexa-fluor 555-cadaverine (red) in buffer containing either calcium (**A**,**C**,**E**,**G**) or, as negative (neg.) control, EDTA (**B**,**D**,**F**,**H**). Nuclear DNA was labeled with Hoechst 33258 (blue). The images were recorded with an Olympus BX63 microscope (**A**,**B**,**E**–**H**) and a Zeiss 980 confocal laser scanning microscope (**C**,**D**). The brightness of the images in panels (**A**–**D**) was uniformly increased by 20%. White dashed lines indicate lower border of the epidermis (**A**–**H**) and white dotted lines indicate the upper border of the epidermis in images of negative control samples (**B**,**D**,**F**,**H**). Scale bars = 20 µm (**A**–**F**), 50 µm (**G**,**H**). der, dermis; epi, epidermis; per, periderm.

TGM activity was also detected in chicken skin models (Figure 4A), in the upper layers of the epidermis of back skin (Figure 4C), reticulate scales (Figure 4E) and scutate scales (Figure 4G) of chicken embryos. The periderm, a transient epithelial layer during embryonic development, did not contain TGM activity, as evidenced by the absence of a TGM activity signal in nucleated cells on the surface of embryonic epidermis and scales (Figure 4C,E,G). Negative control experiments in which calcium was replaced by EDTA confirmed the specificity of labeling in samples (Figure 4B,D,F,H). The labeling was most prominently detected at cell membranes, suggesting that the activity corresponded to TGM1, the only membrane-anchored TGM.

**Figure 5:**
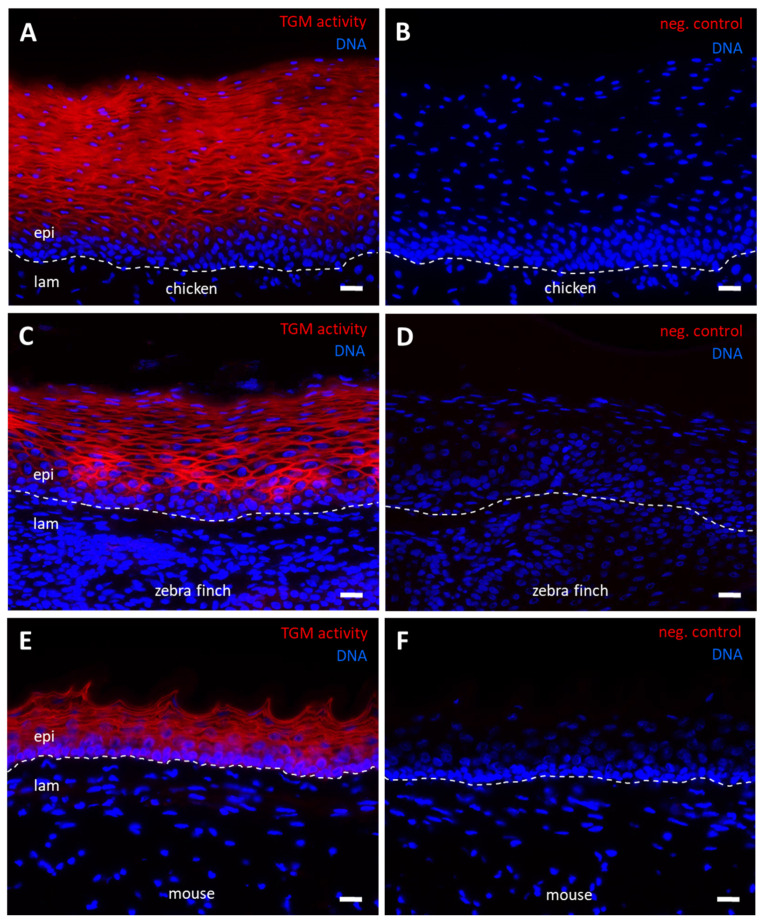
In situ labeling of transglutaminase (TGM) activity in the esophagus. The esophagus of the chicken (**A**,**B**), zebra finch (**C**,**D**) and mouse (**E**,**F**) were cryosectioned and incubated with Alexa-fluor 555-cadaverine (red) in buffer containing either calcium (**A**,**C**,**E**) or, as negative (neg.) control, EDTA (**B**,**D**,**F**). Nuclear DNA was labeled with Hoechst 33258 (blue). The images were recorded with an Olympus BX63 microscope. The brightness of the images was increased by 60% in panels (**C**,**D**) and by 30% in panels **E** and **F**. White dashed lines indicate the basement membrane of esophageal epithelia. Scale bars = 20 µm. epi, epithelium; lam, lamina propria.

TGM activity was also detected in a membrane-associated pattern in the suprabasal layers of the chicken esophageal epithelium, whereas no activity was present in the three most basal layers and in the mesenchymal compartments of the esophagus (Figure 5A,B). This pattern was similar to the patterns of TGM activity in the esophagus of the zebra finch (Figure 5C,D) and the mouse (Figure 5E,F).

## 4. Discussion

Our identification of the complete coding sequence of chicken *TGM1* demonstrates that this enzyme is conserved in the main model species of birds [56,57,58]. Together with the determination of the TGM activity patterns in chicken skin and esophagus, the results of this study close a significant gap in knowledge about the biology of the avian integument. Previous reports showed that epidermal TGM activity can be induced by hydrocortisone in chicken skin [25,26], chicken keratinocytes undergo cornification [27,29] and apparent substrates of transglutamination, such as loricrin and involucrin-like proteins [7,8], are conserved in the chicken. Our results strongly suggest that a major step in epidermal cornification, i.e., TGM1-dependent protein cross-linking on the inner face of the epithelial cell, is active in the chicken.

The sequence conservation of chicken TGM1 includes not only the domain and residues required for catalytic activity, but also the cysteine-rich sequence motif that is palmitoylated for membrane anchorage of the enzyme [59]. Among human and chicken TGMs, TGM1 is the only protein containing such a motif, indicating that it is also the only membrane-bound enzyme capable of protein transglutamination. The TGM activity of terminally differentiated epidermal keratinocytes of the chicken is associated with the cell membranes and thereby resembles the distribution pattern of TGM activity in human skin [41,42]. Even more clearly, TGM activity is membrane-associated in the esophageal epithelium of the chicken. There is no obvious difference in the TGM activity pattern between the chicken and the zebra finch, a representative of songbirds. Therefore, the evolutionary loss of the esophageal transglutamination substrate cornulin in songbirds [38] does not correspond to a loss of transglutamination in general, but rather suggests a change in the composition of the cross-linked proteome of the esophagus of passerines.

In conclusion, terminal differentiation of epidermal keratinocytes and esophageal epithelial cells of the chicken involves expression of *TGM1* and membrane-associated TGM activity, suggesting that transglutamination substrates encoded by chicken EDC genes are cross-linked by mechanisms similar to those of transglutamination in mammalian epidermis and esophagus.

## Data Availability

Data are contained within the article or supplementary material.

## Supplementary Materials

The following are available online at https://www.mdpi.com/article/10.3390/genes12101565/s1, Table S1. Transglutaminases used for phylogenetic analysis. Table S2. Nucleotide sequences used for the assembly of the complete coding sequence of chicken TGM1. Figure S1. Chicken TGM1 sequence assembly. Figure S2. Alignment of trimmed amino acid sequence of transglutaminases used for the phylogenetic analysis. Figure S3. RT-PCR analysis of TGM1 in embryonic skin of the chicken. Figure S4. RT-PCR analysis of zebra finch TGM1 in selected tissues.
