# Supplementary material for: Identification of Chicken Transglutaminase 1 and In Situ Localization of Transglutaminase Activity in Avian Skin and Esophagus
Source: Genes (Basel). 2021 Sep 30;12(10):1565. doi: 10.3390/genes12101565 (PMC8535770; doi:10.3390/genes12101565)
Supplement: Supplementary file 1 [file genes-12-01565-s001.zip › Supplement.pdf]

Table S1. Transglutaminases used for phylogenetic analysis

Species	Gene	NCBI GenBank accession number
Human	TGM1	NP_000350
Human	TGM2	NP_004604
Human	TGM3	NP_003236
Human	TGM4	NP_003232
Human	TGM5	NP_963925
Human	TGM6	NP_945345
Human	TGM7	NP_443187
Human	F13A1	NP_000120
Human	EPB42	NP_000110
Mouse	TGM1	NP_001155186
Mouse	TGM2	NP_033399
Mouse	TGM3	NP_033400
Mouse	TGM4	NP_808579
Mouse	TGM5	NP_083075
Mouse	TGM6	NP_808394
Mouse	TGM7	NP_001153896
Mouse	F13A1	NP_001159863
Mouse	EPB42	NP_038541
Zebra finch	TGM1	XP_041568788
Zebra finch	TGM2	XP_002195997
Zebra finch	TGM3	XP_030144662
Zebra finch	TGM4	XP_041570454
Zebra finch	TGM6	XP_041576223
Zebra finch	TGM5L	XP_030121789
Zebra finch	F13A1	XP_002199785
Zebra finch	EPB42	XP_030144378
Chicken	TGM1	n.a.
Chicken	TGM2	NP_001378925.1
Chicken	TGM3	XP_040544622
Chicken	TGM4	NP_001006368
Chicken	TGM5L	XP_015137927
Chicken	TGM6	XP_040544619
Chicken	TGM6L	XP_015152172
Chicken	F13A1	NP_990016
Chicken	EPB42	NP_001308488
Fruit fly	Tg	NP_609174.1

n.a., not applicable (sequence determined in this study)

Table S2. Nucleotide sequences used for the assembly of the complete coding sequence of chicken TGM1

Sequence type	Accession number	Nucleotide sequence	Translation
RNA-seq read	SRA:SRR13171916.38844678.2	ATGCCGGACGCGGACCCCCGGCTGGACGCGGGCCGTTGGGCTGCTGCCAGCTTCCGCCTGCGGGGGGCGCCGTCC	MPDADPRLDAGRWAAASFRLRGAPS
RNA-seq read	SRA:SRR13171916.4105304.1	GCGCCGTCCTCCGACCCCCCCCCGAGGTCGCGGGGCTCCTTCTGGCGCCGCCTGGGTGGCTGCTGCGGGTGCTGC	APSSDPPPRSRGSFWRRLGGCCGCC
RNA-seq read	SRA:SRR13171916.22961509.1	GCGGGTGCTGCGGGTGCTGCCGGCGCGGCAAGGAGGATTGGGACCCCCCCCCAGGAGAGGTGCCGGGACGGAGC	GCCGCCRRGKEDWDPPPGEVPGRS
RNA-seq read	SRA:SRR13171916.35134332.1	CAAGGAGGATTGGGACCCCCCCCCAGGAGAGGTGCCGGGACGGAGACCCCCCAGGCTTTGAAACCCGGCCTGCTG	KEDWDPPPGEVPGRRPPQALKPGLL
RNA-seq read	SRA:SRR13171916.37813454.2	GCCGACCGCATCGCCCACCACACGGCCGAGTTCTGCTCCCCACAGCTGGTGGTCCGCCGGGGGCAGCCCTTCCAC	ADRIAHHTAEFCSPQLVVRRGQPFH
RNA-seq read	SRA:SRR13171916.34310259.2	GGTTCGAGGGCCGACCGCATCGCCCACCACACGGCCGAGTTCTGCTCCCCACAGCTGGTGGTCCGCCGGGGGCAG	GSRADRIAHHTAEFCSPQLVVRRGQ
RNA-seq read	SRA:SRR13171916.40142191.1	CGCCCACCACACGGCCGAGTTCTGCTCCCCACAGCTGGTGGTCCGCCGGGGGCAGCCCTTCCACCTCCGGGTCCTC	AHHTAEFCSPQLVVRRGQPFHLRVL
RNA-seq read	SRA:SRR13171916.32182126.1	CCCCGAGGACGACAGCCTCTGCGTGGAGCTGCTGCTGGGCCCCACCCCCCAGGTGGCCAAAGGCACTCACGTGCTG	PEDDSLCVELLLGPTPQVAKGTHVL
RNA-seq read	SRA:SRR13171916.34579967.1	CTCGGCCGTGTGGTGGGCGATGCGGTCGGCCCTCGAACCCACAACCAGACCCCGGGGGACCAGCAGGCCGGGTTTC	KPGLLVPRGLVVGSRADRIAHHTAE
RNA-seq read	SRA:SRR13171916.36470078.1	GGGCTCAGCAGCAGCTCCACGCAGAGGCTGTCGTCCTCGGGGTCGAAGGGCCGTGGGAGGAGGACCCGGAGGTGG	HLRVLLPRPFDPEDDSLCVELLLS
RNA-seq read	SRA:SRR13171916.39039682.2	CGGACGGGAGCGGGGGAGTTCGGGGCGCCCTTTGATGACCGCAATGACGTCATCGTGCTGTTCAACCCCTGGTGT	RTGAGEFGAPFDDRNDVIVLFNPWC
RNA-seq read	SRA:SRR13171916.38368388.1	ACGGGAGCGGGGGAGTTCGGGGCGCCCTTTGATGACCGCAATGACGTCATCGTGCTGTTCAACCCCTGGTGTGAG	TGAGEFGAPFDDRNDVIVLFNPWCE
RNA-seq read	SRA:SRR13171916.32667632.1	CATTGGTCGGTACCGCCTGAGCGTCAAAACCCGGACGGGAGCGGGGGAGTTCGGGGCGCCCTTTGATGACCGCAAT	IGRYRLSVKTRTGAGEFGAPFDDRN
RNA-seq read	SRA:SRR13171916.42307715.2	CCGCAATGACGTCATCGTGCTGTTCAACCCCTGGTGTGAGGAGGATGGGGTGTACATGGAGCAAACTAACGACCTT	RNDVIVLFNPWCEEDGVYMEQTNDL
RNA-seq read	SRA:SRR13171916.5625680.2	CAGGTGGCCAAAGGCACTCACGTGCTGATCCCATTGGGCGAAACGTCGGCCACCGGATGGACGGCGGAGCAGGCGG	QVAKGTHVLIPLGETSATGWTAEQA
RNA-seq read	SRA:SRR13171916.29113231.2	ACCGGATGGACGGCGGAGGAGGCGGGAGAGGAAGCGGAAGGGGAGGAGGCGAGCGGAAGTCCCGCCCTCCGGCTCC	TGWTAEEAGEEAEGEEASGSPALRL
RNA-seq read	SRA:SRR13171916.41732328.2	GGGAGGAGGCGAGCGGAAGTCCCGCCCTCCGGCTCCGCCTCTCGGCCCCGGCGGACGCTCCCATTGGTCGGTACCG	EEASGSPALRLRLSAPADAPIGRY
RNA-seq read	SRA:SRR13171916.38559026.1	GTACGTCCTTAACGAGACGGGCCGCATCTTCTATGGCACGGAGGAACAGATCGCAGAGCGCTCCTGGAACTACGGG	YVLNETGRIFYGTEEQIAERSWNYG
RNA-seq read	SRA:SRR13171916.26942286.1	CGTCCTTAACGAGACGGGCCGCATCTTCTATGGCACGGAGGAACAGATCGCAGAGCGCTCCTGGAACTACGGGCAG	VLNETGRIFYGTEEQIAERSWNYGQ
RNA-seq read	SRA:SRR13171916.39820236.1	GGATGGGGTGTACATGGAGCAAACTAACGACCTTAATGAGTACGTCCTTAACGAGACGGGCCGCATCTTCTATGGC	DGVYMEQTNDLNEYVLNETGRIFYG
RNA-seq read	SRA:SRR13171916.27909714.2	AGATCGCAGAGCGCTCCTGGAACTACGGGCAGTTTGACGCAGGGGTCCTGGATGCCTGCTTGGCCATATTGGTC	IAERSWNYGQFDAGVLDACLAILV
RNA-seq read	SRA:SRR13171916.34827418.1	AGGGGTCCTGGATGCCTGCTTGGCCATATTGGACCGGCGCCGGATGCCCCACAGCGCCAGGGGGGACCCCGTGATG	GVLDACLAILDRRRMPHSARGDPVM
RNA-seq read	SRA:SRR13171916.41211803.2	GTTTGACGCAGGGGTCCTGGATGCCTGCTTGGCCATATTGGACCGGCGCCGGATGCCCCACAGCGCCAGGGGGGAC	FDAGVLDACLAILDRRRMPHSARGD
RNA-seq read	SRA:SRR13171916.31179310.1	CCTGGTCACCATCACGGGGTCCCCCCTGGCGCTGTGGGGCATCCGGCGCCGGTCCAATATGGCCAAGCAGGCATCC	DACLAILDRRRMPHSARGDPVMVTR
RNA-seq read	SRA:SRR13171916.25778807.1	CCTCGGTCCAGTTCCCAACCAGAACCCCGTTGTCATCCAGGGAGTTTACCATGGCCGACACCACTCTGGTCACCAT	MVTRVVSAMVNSLDDNGVLVGNWTE
RNA-seq read	SRA:SRR13171916.40268579.1	CCCAGCACTGCCCGTAGCGCACCGGCGCCCCCCCGCGGTGGTAGGAGCGCAGGATGTCCACCGAGCCGGCCCAGGC	AWAGSVDILRSYHRGGAPVRYGQCW
RNA-seq read	SRA:SRR13171916.42053964.2	CTGCGCTCCTACCACCGCGGGGGGGCGCCGGTGCGCTACGGGCAGTGCTGGGTGTTCGCCGGCGTCGTCACCACCG	LRSYHRGGAPVRYGQCWVFAGVVTT
RNA-seq read	SRA:SRR13171916.40167136.1	GTTCGTCACCGTCCGCGTCGGCACCCCCAGGCAGCGCAGCACGGTGGTGACAACGCCGGCGAACACCCAGCACTGC	QCWVFAGVVTTVLRCLGVPTRTVTN
RNA-seq read	SRA:SRR13171916.13320237.2	CTGGTTGGGAACTGGACCGGGGATTACACGCAGGGCACCAACCCGTCGGCCTGGGCCGGCTCGGTGGACATCATGC	LVGNWTGDYTQGTNPSAWAGSVDIM
RNA-seq read	SRA:SRR13171916.37216926.2	CGTGCTGCGCTGCCTGGGGGTGCCGACGCGGACGGTGACGAACTACAACTCGGCGCACGACACCGACGTGTCGCTG	VLRCLGVPTRTVTNYNSAHDTDVSL
RNA-seq read	SRA:SRR13171916.38999933.1	TCCAGACGGAGTCGGTGTTGAGGCGCTCGAGGGGCTTCATGTTCTCGTCGAAGTAGATGTCGGTGGTCAGCGACAC	VSLTTDIYFDENMKPLERLNTDSVW
RNA-seq read	SRA:SRR13171916.40911907.2	GCCCCTCGAGCGCCTCAACACCGACTCCGTCTGGAACTTCCACGTGTGGAACGACTGCTGGATGCGCCGCCCGGCC	PLERLNTDSVWNFHVWNDCWMRRPA
RNA-seq read	SRA:SRR13171916.41474269.1	CCACCACCTGCCAGCCGTCGTATCCCGCAGGGAGGTCCGGGCGGCGCATCCAGCAGTCGTTCCACACGTGGAAGTT	NFHVWNDCWMRRPDLPAGYDGWQVV
RNA-seq read	SRA:SRR13171916.39029076.2	CTGCTGGATGCGCCGCCCGGACCTCCCTGCGGGATACGACGGCTGGCAGGTGGTGGACGCCACCCCGCAGGAGACC	CWMRRPDLPAGYDGWQVVDATPQET
RNA-seq read	SRA:SRR13171916.37760965.2	GTGGACGCCACCCCGCAGGAGACCAGCAGCGGGCTGTTCTGCTGTGGGCCGTGCTCCGTGGCGGCCGTGAAGAACG	VDATPQETSSGLFCCGPCSVAAVKN
RNA-seq read	SRA:SRR13171916.39575733.2	GGGCCGTGCTCCGTGACGGCCGTGAAGAACGGGGAGGTCTTCCTCAAATACGACACTGCCTTCGTCTTCGCCGAGG	GPCSVTAVKNGEVFLKYDTAFVFAE
RNA-seq read	SRA:SRR13171916.38474934.1	GATGGCGAAGGCGCCGTTGCCCTTCCGCTGCCAGTACACCTTGTCGCTGTTCACCTCGGCGAAGACGAAGGCAGTG	TAFVFAEVNSDKVYWQRKGNGAFAI
RNA-seq read	SRA:SRR13171916.2755541.2	GTGAACAGCGACAAGGTGTACTGGCAGCGGAAGGGCAACGGCGCCTTCGCCATCGTCCACGTGGAGGAAGGCGCC	VNSDKVYWQRKGNGAFAIVHVEEGA
RNA-seq read	SRA:SRR13171916.38203596.2	CGACAAGGTGTACTGGCAGCGGAAGGGCAACGGCGCCTTCGCCATCGTCCACGTGGAGGAAGGCGCCATCGGGCGC	DKVYWQRKGNGAFAIVHVEEGAIGR
RNA-seq read	SRA:SRR13171916.35142.1	CTCGGGGTGTTTGTAGAGGTGGGTGATGTCGATGCGGGCGGCCGACTGCGGCCCCACGGTGCTGATGCGGCGCCCG	GRRISTVGPQSAARIDITHLYKHPE
RNA-seq read	SRA:SRR13171916.36952746.2	ATCGACATCACCCACCTCTACAAACACCCCGAGGGCTCGGAGGCGGAGCGTCGCGCGGTGTCCACGGCGACATCTC	IDITHLYKHPEGSEAERRAVSTATS
RNA-seq read	SRA:SRR13171916.42145422.2	CTGCGCGCTGCGGTACACCGGAGTGGCGGCGCCGCCCTTCCGCCACGAGCAGCACAGGAGGGTCGTGGCGCCGGGG	CALRYTGVAAPPFRHEQHRRVVAPG
RNA-seq read	SRA:SRR13171916.40967521.1	CCTGTGCTGCTCGTGGCGGAAGGGCGGCGCCGCCACTCCGGTGTACCGCAGCGCGCAGACGGACAGCCGCACCCGG	RVRLSVCALRYTGVAAPPFRHEQHR
RNA-seq read	SRA:SRR13171916.37909467.2	CACAGGAGGGTCGTGGCGCCGGGGGGGGAGGAGCAGCTGTGTGTGGCGGTGCCGTTCTCGGAGTACAGCCCCCACG	HRRVVAPGGEEQLCVAVPFSEYSPH
RNA-seq read	SRA:SRR13171916.40442065.1	CCTGGGACGGCGCCGCGCCGCTGCGAGGCCGGGAGCCGTGAGATGTCGCCGTGGACACCGCGCGACGCTCCGCCTC	EAERRAVSTATSHGSRPRSGAAPSQ
RNA-seq read	SRA:SRR13171916.36501675.1	CTTTCAGCTCCAACTCCGCCCCCGCCACCGCCGGCCCGGAGCTCAGCGACAGCCGCACCTCCCCCCGGGACGGCGC	APSRGEVRLSLSSGPAVAGAELELK
RNA-seq read	SRA:SRR13171916.42107183.1	GCCGCACCCGGACGGTCCGCGGCTGCGGAGCCGCGTTGTGAGCCGTCACTTTCAGCTCCAACTCCGCCCCCGCCAC	VAGAELELKVTAHNAAPQPRTVRVR
RNA-seq read	SRA:SRR13171916.38874248.2	GGAGCAGCTGTGTGTGGCGGTGCCGTTCTCGGAGTACAGCCCCCACGTGGGCTCTCAGGACGCGCTCCGTCTGACG	EQLCVAVPFSEYSPHVGSQDALRLT
RNA-seq read	SRA:SRR13171916.34832449.1	CGCCGCCGCCGTCAGACGGAGCGCGTCCTGAGAGCCCACGTGGGGGCTGTACTCCGAGAACGGCACCGCCACACAC	CVAVPFSEYSPHVGSQDALRLTAAA
RNA-seq read	SRA:SRR13171916.19625563.2	AAAGAGCTGCGCGTCCGCCTGGCGGCCCCCGACCTCAGCATGACGCTGCTGGGCCCCCCGGTTGTAGGGCAGGAGG	KELRVRLAAPDLSMTLLGPPVVGQE
RNA-seq read	SRA:SRR13171916.29430382.2	TCTCAGGACGCGCTCCGTCTGACGGCGGCGGCGGCGGTGGAGGAGACGGGGGAGGTGGTGGCCAAAGAGCTGCGCG	SQDALRLTAAAAVEETGEVVAKELR
RNA-seq read	SRA:SRR13171916.41578201.2	GGGCAGGAGGTTTCCGTTCAGGTCCTCTTCCGGAATCCGCTGCCCCAGAAGCTCACGGGGGCGGAGCTTCGCATGG	GQEVSVQVLFRNPLPQKLTGAELRM
RNA-seq read	SRA:SRR13171916.17212336.1	TGATGGATGCGGGGCAGGACAGCCCCGCCCCCTCCATGCGAAGCTCCGCCCCCGTGAGCTTCTGGGGCAGCGGATT	NPLPQKLTGAELRMEGAGLSCPASI
RNA-seq read	SRA:SRR13171916.34935769.1	CTGACGGAGCCGCAGGGTCTGCTCCGGGGCCACCGTGCCGACGCTGATGGATGCGGGGCAGGACAGCCCCGCCCCC	GAGLSCPASISVGTVAPEQTLRLRQ
RNA-seq read	SRA:SRR13171916.38756346.1	GTCGGGTCCCGGGACGGCGTCGAATTGCAGCTCTCCGTGGACGGGGCCCAACTGGGCGCTTTCCATGGCGGCCACC	VAAMESAQLGPVHGELQFDAVPGPD
RNA-seq read	SRA:SRR13171916.41251451.1	CGTTGGTTGCCGCGCTGCCCTCCACGCTGTCCCCGTCGGGTCCCGGGACGGCGTCGAATTGCAGCTCTCCGTGGAC	VHGELQFDAVPGPDGDSVEGSAATN
RNA-seq read	SRA:SRR13171916.37698911.1	GGGCGCTTTCCATGGCGGCCACCAACCGACGGCGCCCGGCCCGCAGAGGCACCACGGGCTGACGGAGCCGCAGGGT	TLRLRQPVVPLRAGRRRLVAAMESA
RNA-seq read	SRA:SRR13171916.26215572.1	GCCTCCTCCTCCTCGTGTTGCCGTTGGTTGCCGCGCTGCCCTCCACGCTGTCCCCGTCGGGTCCCGGGACGGCGTC	DAVPGPDGDSVEGSAATNGNTRRRR
Whole genome shotgun sequence	JAENSL010000336.1:1785-2039	ACGGTGGCCCCGGAGCAGACCCTGCGGCTCCGTCAGCCCGTGGTGCCTCTGCGGGCCGGGCGCCGTCGGTTGGTGGCCGCCAT<br>GGAAAGCGCCCAGTTGGGCCCCGTCCACGGAGAGCTGCAATTCGACGCCGTCCCGGGACCCGACGGGGACAGCGTGGAGGGC<br>AGCGCGGCAACCAACGGCAACACGAGGAGGAGGAGGAGGGGGGGAGGGGGGCGGCGCCGGGGGGGGAGGGCGGGGGGG<br>AGCACCGGGGGG	TVAPEQTLRLRQPVVPLRAGRRRLVAAMESA<br>QLGPVHGELQFDAVPGPDGDSVEGSAATNG<br>NTRRRRRGGGGRRRGGRAGGSTGG

Abbreviations: RNA-seq, RNA-sequencing; SRA, sequence read archive

A

```
                        1                                                                                              100
TGM1 human              MMDG-PRSDVGRWGGNPLQPPTTPSPEPEPEPD----GRSRRGGGRSFWARCCGCCSCRNAADDDWGPEPSDSRGRGSSSGTRRPGSRGSDSRRPVSRGS
TGM1 zebra finch        MPD--PRLDRGRFP--SLRLTGAPRAPPPSGRRAGLWRRLAAAGGN---CGCCGCCGRSGGGGGEWEPIPGETPGRRTAQ-------------------
TGM1 chicken            MPDADPRLDAGRWAAASFRLRGAPSSDPPPRSRGSFWRRL---GGC---CGCCGCCRR---GKEDWDPPPGEVPGRRPPQ-------------------
SRR13171916.38844678.2  MPDADPRLDAGRWAAASFRLRGAPS
SRR13171916.4105304.1                         APSSDPPPRSRGSFWRRL---GGC---CGCC
SRR13171916.22961509.1                                                    GCCGCCRR---GKEDWDPPPGEVPGRS
SRR13171916.35134332.1                                                                KEDWDPPPGEVPGRRPPQ-------------------

                        101                                                                                            200
TGM1 human              GVNAAGDGTIREGMLVVNGVDLLSSRSDQNRREHHTDEYEYDELIVRRGQPFHMLLLLSRTYE-SSDRITLELLIGNNPEVGKGTHVIIPVGKGGSGGWK
TGM1 zebra finch        --------PVRPGLLAPVW---LRPGSSTNRTAHHTQEFPARPLVVRRGQRFHVGMRLPRE-PRTGDGICLELTLGPNPQVAKGTHVLVPLGGSSPTGWS
TGM1 chicken            --------ALKPGLLVPRG---LVVGSRADRIAHHTAEFCSPQLVVRRGQPFHLRVLLPRPFDPEDDSLCVELLLGPTPQVAKGTHVLIPLGETSATGWT
SRR13171916.35134332.1  --------ALKP
SRR13171916.34579967.1  ----------KPGLLVPRG---LVVGSRADRIAHHTAE
SRR13171916.34310259.2                           GSRADRIAHHTAEFCSPQLVVRRGQ
SRR13171916.37813454.2                              ADRIAHHTAEFCSPQLVVRRGQPFH
SRR13171916.40142191.1                                  AHHTAEFCSPQLVVRRGQPFHLRVL
SRR13171916.36470078.1                                                      HLRVLLPRPFDPEDDSLCVELLLS
SRR13171916.32182126.1                                                                 PEDDSLCVELLLGPTPQVAKGTHVL
SRR13171916.5625680.2                                                                                  QVAKGTHVLIPLGETSATGWT
SRR13171916.29113231.2                                                                                                  TGWT

                        201                                                                                            300
TGM1 human              AQVVK----------ASGQNLNLRVHTSPNAIIGKFQFTVRTQSDAGEFQLPFDPRNEIYILFNPWCPEDIVYVDHEDWRQEYVLNESGRIYYGTEAQIG
TGM1 zebra finch        AEVDEGVAEPIGGGANSGHALWVGLTAPPTAPIGRYRLSARTRTEAGEFAAPFEPDNDLVLLFNPWCQEDSVYMEQTSDLSEYVLNESGRIFYGTEEQIA
TGM1 chicken            AE--EAGEEAEGEEASGSPALRLRLSAPADAPIGRYRLSVKTRTGAGEFGAPFDDRNDVIVLFNPWCEEDGVYMEQTNDLNEYVLNETGRIFYGTEEQIA
SRR13171916.5625680.2   AE--QA
SRR13171916.29113231.2  AE--EAGEEAEGEEASGSPALRL
SRR13171916.41732328.2              EEASGSPALRLRLSAPADAPIGRY
SRR13171916.32667632.1                                  IGRYRLSVKTRTGAGEFGAPFDDRN
SRR13171916.39039682.2                                            RTGAGEFGAPFDDRNDVIVLFNPWC
SRR13171916.38368388.1                                             TGAGEFGAPFDDRNDVIVLFNPWCE
SRR13171916.42307715.2                                                         RNDVIVLFNPWCEEDGVYMEQTNDL
SRR13171916.39820236.1                                                                       DGVYMEQTNDLNEYVLNETGRIFYG
SRR13171916.38559026.1                                                                                    YVLNETGRIFYGTEEQIA
SRR13171916.26942286.1                                                                                     VLNETGRIFYGTEEQIA
SRR13171916.27909714.2                                                                                                    IA

                        301                                                                                            400
TGM1 human              ERTWNYGQFDHGVLDACLYILDRRGMPYGGRGDPVNVSRVISAMVNSLDDNGVLIGNWSGDYSRGTNPSAWVGSVEILLSYLRTGYSVPYGQCWVFAGVT
TGM1 zebra finch        ERAWNYGQFEPGVLDACLFILDRRGMPHGARGDPVMVARVVSAMVNSLDDSGVLVGNWTGDYSQGTNPSAWAGSVGILRSFHGTGSPVRYGQCWVFAGVV
TGM1 chicken            ERSWNYGQFDAGVLDACLAILDRRRMPHSARGDPVMVTRVVSAMVNSLDDNGVLVGNWTGDYTQGTNPSAWAGSVDILRSYHRGGAPVRYGQCWVFAGVV
SRR13171916.38559026.1  ERSWNYG
SRR13171916.26942286.1  ERSWNYGQ
SRR13171916.27909714.2  ERSWNYGQFDAGVLDACLAILV
SRR13171916.41211803.2          FDAGVLDACLAILDRRRMPHSARGD
SRR13171916.34827418.1             GVLDACLAILDRRRMPHSARGDPVM
SRR13171916.31179310.1                DACLAILDRRRMPHSARGDPVMVTR
SRR13171916.25778807.1                                   MVTRVVSAMVNSLDDNGVLVGNWTE
SRR13171916.13320237.2                                                     LVGNWTGDYTQGTNPSAWAGSVDIM
SRR13171916.40268579.1                                                                     AWAGSVDILRSYHRGGAPVRYGQCW
SRR13171916.42053964.2                                                                             LRSYHRGGAPVRYGQCWVFAGVV
SRR13171916.40167136.1                                                                                         QCWVFAGVV

                        401                                                                                            500
TGM1 human              TTVLRCLGLATRTVTNFNSAHDTDTSLTMDIYFDENMKPLEHLNHDSVWNFHVWNDCWMKRPDLPSGFDGWQVVDATPQETSSGIFCCGPCSVESIKNGL
TGM1 zebra finch        TTVLRCLGLPTRTVTNYNSAHDTDTSLTTDIYLDEAMRPLERLNTDSVWNFHVWNDCWMKRPDLPEGYDGWQVVDATPQETSSGLFCCGPCSVKAVKNGD
TGM1 chicken            TTVLRCLGVPTRTVTNYNSAHDTDVSLTTDIYFDENMKPLERLNTDSVWNFHVWNDCWMRRPDLPAGYDGWQVVDATPQETSSGLFCCGPCSVTAVKNGE
SRR13171916.42053964.2  TT
SRR13171916.40167136.1  TTVLRCLGVPTRTVTN
SRR13171916.37216926.2    VLRCLGVPTRTVTNYNSAHDTDVSL
SRR13171916.38999933.1                          VSLTTDIYFDENMKPLERLNTDSVW
SRR13171916.40911907.2                                        PLERLNTDSVWNFHVWNDCWMRRPA
SRR13171916.41474269.1                                                   NFHVWNDCWMRRPDLPAGYDGWQVV
SRR13171916.39029076.2                                                          CWMRRPDLPAGYDGWQVVDATPQET
SRR13171916.37760965.2                                                                           VDATPQETSSGLFCCGPCSVAAVKN
SRR13171916.39575733.2                                                                                          GPCSVTAVKNGE

                        501                                                                                            600
TGM1 human              VYMKYDTPFIFAEVNSDKVYWQRQDDGSFKIVYVEEKAIGTLIVTKAISSNMREDITYLYKHPEGSDAERKAVETAAAHGSKPNVYANRGSAEDVAMQVE
TGM1 zebra finch        VFLKYDTPFVFAEVNSDKVYWQRQAHGGFAVVHVEEGAIGRRISTLGATGHARVDITDQYKHPEGSEEERRAVSAATSHGSRPRQRGPTSGGE-VTMTVG
TGM1 chicken            VFLKYDTAFVFAEVNSDKVYWQRKGNGAFAIVHVEEGAIGRRISTVGPQSAARIDITHLYKHPEGSEAERRAVSTATSHGSRPRSGAAPSRGE-VRLSLS
SRR13171916.39575733.2  VFLKYDTAFVFAE
SRR13171916.38474934.1        TAFVFAEVNSDKVYWQRKGNGAFAI
SRR13171916.2755541.2                VNSDKVYWQRKGNGAFAIVHVEEGA
SRR13171916.38203596.2                  DKVYWQRKGNGAFAIVHVEEGAIGR
SRR13171916.35142.1                                            GRRISTVGPQSAARIDITHLYKHPE
SRR13171916.36952746.2                                                       IDITHLYKHPEGSEAERRAVSTATS
SRR13171916.40442065.1                                                                    EAERRAVSTATSHGSRPRSGAAPSQ
SRR13171916.36501675.1                                                                                         APSRGE-VRLSLS

                        601                                                                                            700
TGM1 human              AQDAVMGQDLMVSVMLINHSSSRRTVKLHLYLSVTFYTGVSGTIFKETKKEVELAPGASDRVTMPVAYKEYRPHLVDQGAMLLNVSGHVKESGQVLAKQH
TGM1 zebra finch        SGPAVAGADLELRAVLKNGGLEPRTLRLRFSLCAVRYTGVAGAAFRQEQHRRTLPPGQEDTVTMTVTYTEYQPHVGDQDALKLTVAAAVQESGQVLAKEL
TGM1 chicken            SGPAVAGAELELKVTAHNAAPQPRTVRVRLSVCALRYTGVAAPPFRHEQHRRVVAPGGEEQLCVAVPFSEYSPHVGSQDALRLTAAAAVEETGEVVAKEL
SRR13171916.36501675.1  SGPAVAGAELELK
SRR13171916.42107183.1      VAGAELELKVTAHNAAPQPRTVRVR
SRR13171916.40967521.1                            RVRLSVCALRYTGVAAPPFRHEQHR
SRR13171916.42145422.2                                  CALRYTGVAAPPFRHEQHRRVVAPG
SRR13171916.37909467.2                                                   HRRVVAPGGEEQLCVAVPFSEYSPH
SRR13171916.38874248.2                                                             EQLCVAVPFSEYSPHVGSQDALRLT
SRR13171916.34832449.1                                                                CVAVPFSEYSPHVGSQDALRLTAAA
SRR13171916.29430382.2                                                                              SQDALRLTAAAAVEETGEVVAKEL
SRR13171916.19625563.2                                                                                                   KEL

                        701                                                                                            800
TGM1 human              TFRLRTPDLSLTLLGAAVVGQECEVQIVFKNPLPVTLTNVVFRLEGSGLQRPKILNVGDIGGNETVTLRQSFVPVRPGPRQLIASLDSPQLSQVHGVIQV
TGM1 zebra finch        LVRLHTPELTLTLLGPAVVGRPVSVQVVFQNPLPAPLPSASLRMEGAGIACPKPLALGSLGAGQTLRLSQSVTPLRAGQRRLVATLESAALPPLSGCVQF
TGM1 chicken            RVRLAAPDLSMTLLGPPVVGQEVSVQVLFRNPLPQKLTGAELRMEGAGLSCPASISVGTVAPEQTLRLRQPVVPLRAGRRRLVAAMESAQLGPVHGELQF
SRR13171916.29430382.2  R
SRR13171916.19625563.2  RVRLAAPDLSMTLLGPPVVGQE
SRR13171916.41578201.2                     GQEVSVQVLFRNPLPQKLTGAELRM
SRR13171916.17212336.1                                NPLPQKLTGAELRMEGAGLSCPASI
SRR13171916.34935769.1                                               GAGLSCPASISVGTVAPEQTLRLRQ
SRR13171916.37698911.1                                                                  TLRLRQPVVPLRAGRRRLVAAMESA
SRR13171916.38756346.1                                                                                    VAAMESAQLGPVHGELQF
SRR13171916.41251451.1                                                                                               VHGELQF
JAENSL010000336.1-part                                                            TVAPEQTLRLRQPVVPLRAGRRRLVAAMESAQLGPVHGELQF

                        801                                          849
TGM1 human              DVAPAPGDGGFFSDAGGDSHLGETIPMASRGGA----------------
TGM1 zebra finch        SAQT--GSTGSTGSLTGDTGREENHTGSTGNGGRRLRRRRGRPGGSTGG
TGM1 chicken            DAVP--GPDGD--SVEGSAATNGNTRRRRRGGGGR--RRGGRAGGSTGG
SRR13171916.38756346.1  DAVP--GPD
SRR13171916.41251451.1  DAVP--GPDGD--SVEGSAATN
SRR13171916.26215572.1  DAVP--GPDGD--SVEGSAATNGNTRRRR
JAENSL010000336.1-part  DAVP--GPDGD--SVEGSAATNGNTRRRRRGGGGR--RRGGRAGGSTGG
```

B

```
>Gallus gallus TGM1 mRNA (coding sequence)
atgccggacgcggacccccggctggacgcgggccgttgggctgctgccagcttccgcctgcgggggcgccgtcctccgaccc
cccccgaggtcgcggggctccttctggcgccgcctgggtggctgctgcgggtgctgcgggtgctgccggcgcggcaaggagg
attgggaccccccccaggagaggtgccgggacggagaccccccaggctttgaaacccggcctgctggtcccccggggtctg
gttgtgggttcgagggccgaccgcatcgcccaccacacggccgagttctgctccccacagctggtggtccgccgggggcagcc
cttccacctccgggtcctcctcccacggcccttcgaccccgaggacgacagcctctgcgtggagctgctgctgggccccaccc
cccaggtggccaaaggcactcacgtgctgatcccattgggcgaaacgtcggccaccggatggacggcggaggaggcgggagag
gaagcggaaggggaggaggcgagcggaagtcccgccctccggctccgcctctcggccccggcggacgctcccattggtcggta
ccgcctgagcgtcaaaacccggacgggagcgggggagttcggggcgccctttgatgaccgcaatgacgtcatcgtgctgttca
acccctggtgtgaggaggatggggtgtacatggagcaaactaacgaccttaatgagtacgtccttaacgagacgggccgcatc
ttctatggcacggaggaacagatcgcagagcgctcctggaactacgggcagtttgacgcaggggtcctggatgcctgcttggc
catattggaccggcgccggatgccccacagcgccagggggaccccgtgatggtgaccagagtggtgtcggccatggtaaact
ccctggatgacaacggggttctggttgggaactggaccggggattacacgcagggcaccaacccgtcggcctgggccggctcg
gtggacatcctgcgctcctaccaccgcgggggggcgccggtgcgctacgggcagtgctgggtgttcgccggcgttgtcaccac
cgtgctgcgctgcctgggggtgccgacgcggacggtgacgaactacaactcggcgcacgacaccgacgtgtcgctgaccaccg
acatctacttcgacgagaacatgaagcccctcgagcgcctcaacaccgactccgtctggaacttccacgtgtggaacgactgc
tggatgcgccgcccggacctccctgcgggatacgacggctggcaggtggtggacgccaccccgcaggagaccagcagcgggct
gttctgctgtgggccgtgctccgtgacggccgtgaagaacggggaggtcttcctcaaatacgacactgccttcgtcttcgccg
aggtgaacagcgacaaggtgtactggcagcggaagggcaacggcgccttcgccatcgtccacgtggaggaaggcgccatcggg
cgccgcatcagcaccgtggggccgcagtcggccgcccgcatcgacatcacccacctctacaaacaccccgagggctcggaggc
ggagcgtcgcgcggtgtccacggcgacatctcacggctcccggcctcgcagcggcgcggcgccgtcccgggggggaggtgcggc
tgtcgctgagctccgggccggcggtggcgggggcggagttggagctgaaagtgacggctcacaacgcggctccgcagccgcgg
accgtccgggtgcggctgtccgtctgcgcgctgcggtacaccggagtggcggcgccgcccttccgccacgagcagcacaggag
ggtcgtggcgccgggggggggaggagcagctgtgtgtggcggtgccgttctcggagtacagcccccacgtgggctctcaggacg
cgctccgtctgacggcggcggcggcggtggaggagacgggggaggtggtggccaaagagctgcgcgtccgcctggcggccccc
gacctcagcatgacgctgctgggcccccccggttgtagggcaggaggtttccgttcaggtcctcttccggaatccgctgcccca
gaagctcacgggggcggagcttcgcatggagggggcggggctgtcctgccccgcatccatcagcgtcggcacggtggccccgg
agcagaccctgcggctccgtcagcccgtggtgcctctgcgggccgggcgccgtcggttggtggccgccatggaaagcgcccag
ttgggccccgtccacggagagctgcaattcgacgccgtcccgggacccgacggggacagcgtggagggcagcgcggcaaccaa
cggcaacacgaggaggaggaggagggggggagggggggcggcgccgggggggggagggcggggggggagcaccggggggtga
```

C

```
>Gallus gallus TGM1
MPDADPRLDAGRWAAASFRLRGAPSSDPPPRSRGSFWRRLGGCCGCCGCCRRGKEDWDPPPGEVPGRRPPQALKPGLLVPRGL
VVGSRADRIAHHTAEFCSPQLVVRRGQPFHLRVLLPRPFDPEDDSLCVELLLGPTPQVAKGTHVLIPLGETSATGWTAEEAGE
EAEGEEASGSPALRLRLSAPADAPIGRYRLSVKTRTGAGEFGAPFDDRNDVIVLFNPWCEEDGVYMEQTNDLNEYVLNETGRI
FYGTEEQIAERSWNYGQFDAGVLDACLAILDRRRMPHSARGDPVMVTRVVSAMVNSLDDNGVLVGNWTGDYTQGTNPSAWAGS
VDILRSYHRGGAPVRYGQCWVFAGVVTTVLRCLGVPTRTVTNYNSAHDTDVSLTTDIYFDENMKPLERLNTDSVWNFHVWNDC
WMRRPDLPAGYDGWQVVDATPQETSSGLFCCGPCSVTAVKNGEVFLKYDTAFVFAEVNSDKVYWQRKGNGAFAIVHVEEGAIG
RRISTVGPQSAARIDITHLYKHPEGSEAERRAVSTATSHGSRPRSGAAPSRGEVRLSLSSGPAVAGAELELKVTAHNAAPQPR
TVRVRLSVCALRYTGVAAPPFRHEQHRRVVAPGGEEQLCVAVPFSEYSPHVGSQDALRLTAAAAVEETGEVVAKELRVRLAAP
DLSMTLLGPPVVGQEVSVQVLFRNPLPQKLTGAELRMEGAGLSCPASISVGTVAPEQTLRLRQPVVPLRAGRRRLVAAMESAQ
LGPVHGELQFDAVPGPDGDSVEGSAATNGNTRRRRRGGGGRRRGGRAGGSTGG
```

Figure S1. Chicken TGM1 sequence assembly. (A) Alignment of amino acid sequences of TGM1 proteins of human and zebra finch versus translated sequences of chicken TGM1 nucleotide sequence fragments. RNA-sequencing reads from chicken in vitro skin models were subjected to tBLASTn search for TGM1. The sequences of the RNA-seq reads are shown in Supplementary Table S1. **(B)** Nucleotide sequence of the coding region of chicken (*Gallus gallus*) TGM1 mRNA. **(C)** Amino acid sequence of chicken (*Gallus gallus*) TGM1 protein.

```
>mouse_TGM1
LVVTGVDLLCDQNRREHHTDEFEYDELIVRRGQPFHMILFLNREYESSDRIALELLIGSNP
EVGKGTHVIIPVGKGGSGGWKAQVTKNNGHNLNLRVHTSPNAIIGKFQFTVRTRSELPFDP
RNEIYILFNPWCPEDIVYVDHEDWRQEYVLNESGRIYYGTEAQIGERTWNYGQFDHGVLDA
CLYILDRRGMPYGGRGDPVSVSRVVSAMVNSLDDNGVLIGNWTGDYSRGTNPSAWVGSVEI
LLSYLRTGYSVPYGQCWVFAGVTTTVLRCLGFATRTVTNFNSAHDTDTSLTMDIYFDENMK
PLHLNHDSVWNFHVWNDCWMKRPDLPSGFDGWQVVDATPQETSSGIFCCGPCSVESVKNGL
VYMKYDTPFIFAEVNSDKVYWQRQDDGSFKIVYVEEKAIGTLIVTKAIHSNNREDITHIYK
HPEGSEAERRAVEKAAAHGSKPNVYATRDSAEDVAMQVEAQDAVMGQDLAVSVVLTNRGSS
RRTVKLHLYLCVTYYTGVSGPTFKEAKKEVTLAPGASDSVTMPVAYKEYKPHLVDQGAMLL
NVSGHVESGQVLAKQHTFRLRTPDLSLTLLGAAVVGQECGVQIVFKNPLPVTLTNVVFRLE
GSGLQRPKVLNVGDIGGNETVTLRQTFVPVRPGPRQLIASLDSPQLSQVHGVIQVDVAP
>mouse_TGM2
LLLERCDLEIQANGRDHHTADLCQEKLVLRRGQRFRLTLYFGRGYEASDSLTFGAVTGPDP
SEEAGTKARFSLSDVEEGSWSASVLDQQDNVLSLQLCTPANAPIGLYRLSLEASTGGSSFV
LGHFILLYNAWCPADDVYLDSEEERREYVLTQQGFIYQGSVKFIKSVPWNFGQFEDGILDT
CLMLLDMNSRDCSRRSSPIYVGRVVSAMVNCNDDQGVLLGRWDNNYGDGISPMAWIGSVDI
LRRWKEHGQQVKYGQCWVFAAVACTVLRCLGIPTRVVTNYNSAHDQNSNLLIEYFRNEFGE
LESNKSEMIWNFHCWVESWMTRPDLQPGYEGWQAIDPTPQEKSEGTYCCGPVSVRAIKEGD
LSTKYDAPFVFAEVNADVVDWIRQEDGSVLKSINRSLVVGQKISTKSVGRDDREDITHTYK
YPEGSPEEREVFTKANHLNKLAE-----
KEETGVAMRIRGDSMSMGNDFDVFAHIGNDTSETRECRLLLCARTVSYNGVLGPECTED-
INLTLDPYSENSIPLRILYEKYSGCLTESNLIKVRGLLIEAANSYLLAERDLYLENPEIKI
RVLGEPKQNRKLVAEVSLKNPLSDPLYDCIFTVEGAGLTKQKSVEVSDVPAGDLVKARVDL
FPTDIGLHKLVVNFQCDKLKSVKGYRNVIIGP
>mouse_TGM3
LQIQNVNWQVPMNRRAHHTDKFSSQDSIVRRGQPWEIILVCNRSLESGEDLNFIVSTGPQP
SESARTKAVFSISGRSTGGWNAALKANSGNNLAIAIASPVSAPIGLYTLSVEISSRASSLK
LGTFIMLFNPWLQADDVFMSNHAERQEYVEEDSGIIYVGSTNRIGMVGWNFGQFEEDILNI
SLSILDRSVTDVARRNDPKYVCRVLSAMINGNDDNGVISGNWSGNYTGGVDPRTWNGSVEI
LKNWKKSGRPVQFGQCWVFAGTLNTVLRCLGVPSRVITNFNSAHDTDRNLSVDVYYDAMGN
PLEKGSDSVWNFHVWNEGWFVRTDLGPTYNGWQVLDATPQERSQGVFQCGPASVNAIKAGD
VDRNFDMIFIFAEVNADRITWIYNNRNTQKQNSVDTHSIGKYISTKAVGSNSRMDVTDKYK
YPEGSSEERQVHQKALDKLKPNASFGAEDKEPSISGKFKTGILAVGKEVSLSLMLKNMTND
RKTVTMNMTAWTIVYNGTLVHEVWKDSATISLDPEEEIQYPVKIAYSQYERYLKADNMIRI
TAVCKVPDEAEVVVERDVILDNPALTLEVLEQAHVRKPVNVQMLFSNPLDQPVNNCVLLVE
GSGLLRSLKIDVPSLRPKEKSRIRFEIFPTRSGTKQLLADFSCNKFPAIKAMLPIDVSE
>mouse_TGM4
LIIYAVNVERKLNAAAHHTSEYQTKKLVLRRGQIFTLKVILNRPLQPQDELKVTFTSG---
QRDPPYMVELDPVTYRSKGWQVKIAKQSGVEVILNVISAADAVVGRYKMRVN------
EYKAGVFYLLFNPWCSDDSVFMASEEERAEYILNDTGYMYMGFAKQIKEKPWTFGQFEKHI
LSCCFNLLF--QLENNEMQNPVLVSRAICTMMCAA-
NGGVLMGNWTGDYADGTAPYVWTSSVPILQQHYVTRMPVRYGQCWVFSGILTTALRAVGIP
ARSVTNFESAHDTEKNLTVDIYLDESGKTIHLTKDSVWNFHVWTDAWMKRQDLPHGYDGWQ
VLDSTPQEISDGGFRTGPSPLTAIRQGLIQMKYDTTFVFTEVNGDKFIWLVKQNEKNILIA
VETASIGKKISTKMVGENRREDITLQYKFPEGSPEERKVMAKASGKPSDDKLNSR-
TLNNSLQISVLQNSLELGAPIYLTITLKRKTATPQNVNISCSLNLQTYTGNKKTNLGVIQK
TVQIHGQES-
RVFLTMDASYYIYKLGDEMVIGGFIIAEIDSGERVATDTTLCFLYSAFSVEMPSTGKVKQP
LVITSKFTNTLPIPLTNIKFSVESLGLANMKSWEQETVPPGKTITFQMECTPVKAGPQKFI
VKFISRQVKEVHAEKVVLISK
>mouse_TGM5
LEVALTDLQSSQNNVRHHTEEISVDRLVVRRGQAFSITLYFNRGFQPGDSIMFVAETGPLP
DLAKGTRAVFSFTGGGPSPWIASLEANRANSLEVSLCAPPIAAVGRYLLKIRIDSYVTAYQ
LGEFILLFNPWCPADSVYLESEPQRQEYVVNDYGFIYQGSKSWIRPCPWNYGQFEENIIDI
CLELLEKSSTDCALRGSPVYTSRVVCAMINSNDDNGVLNGNWSENYVDGINPAEWTGSVAI
LKQWHATGQPVRYGQCWVFAAVMCTVMRCLGIPTRVITNFDSGHDTDGNLIIDEYYDNTGR
ILNMKKDTVWNFHVWNECWMARKDLPPGYGGWQVLDATPQETSNGLYCCGPASVKAIKEGE
IDLNYDTRFAFSMVNADCMSWLVYG-
GKEQKLHQDTATVGNFISTKSIQSDERDDITESYKYEEGSLQEREVFLKALQKLQATRSQG
PPSDVLQVSLKFELDSPKMGQDINFVLLAVNMSPQFKDLKLNLSAQSLLHDGSPLVPFWQD
TAFITLFPEEEKSYPCKILYSQYSQYLSTDKLIRISALGEENSPEKILVNKIITLTFPGIM
```

INVLGAAFVNQPLTVQVVFSNPLSEPVEDCVLTLEGSGLFRQQRVLIGVLKPHHKASITLK
TVPFKSGQRQIQANLRSNRFKDIKGYKNVYVDI
>mouse_TGM6
VRITKVDWQHSKNGAAHHTQDYPCSELVVRRGQLFSLTLDLSRVLDSEEALIFTVETGPHA
SEALHTKAVFQTAEEIGDTWTAAKEEQTENTITVSLSSPSNAVIGRYLLSVRPSSRHSDRK
LGQFILLFNPWCPEDDVFLDSEEERQEYVLNDSGVIFRGVEKHIRAQGWNYGQFEEDILNI
CLSILDRSATDVSHRHDPVYVTRIISAMVNSNNDRGVVQGQWQGKYGGGTNPLNWRGSVAI
LQKWFKGRKPVKYGQCWVFAGVMCTVLRCLGIATRVVSNFNSAHDTDGNLSVDKYVDSYGR
TLDLTEDSMWNFHVWNESWFARQDLGPSYDGWQVLDATPQEESEGMFRCGPASVTAIREGD
VHLAHDGPFVFAEVNADYITWLWHEDKRRERVYSDTKKIGRCISTKAVGSDSRVDITGLYK
YPEGSRKERQVYSKAVKKLLSVEAWGRPVTKPSITGKFKLEPPVLGQDLKLALCLTNLTAR
AQRVRVNVSGATILYTRKPVAEILRESHTVKLGPLEEKKIPVTISYSQYKGDLTEDKKILL
AAMCLVSKGEKLLVEKDITLED-
FITIKVLGPAVVGVTVTVEVLVINPLSESVKDCVLMVEGSGLLQQLSIEVPSLQPQEKALI
QFNITPSKSGPRQLQVDLVSSQFPDIKGFVIIHVAT
>mouse_TGM7
LELRSVELQIPRNNKDHHTEDLGLSKLILRRGQIFILTIVFSRPFQPQDHLTFVAETGPEP
TELLGTRATFFLAQRPENVWSASDFMIDSNSLQVSIFSPASAVIGHYTLKLEISQGGVTYP
LGNFILLFNPWSTEDDVYLPSETLLREYIMSDYGFIYKGQTNSITSRPWNYGQFEEDIVDI
CFEILNKSSKDHSQRNNVVYVCRVVSAMINSNDDSGVLQGNWGEDYSQGTSPLEWNGSVAI
LRQWSAKGQPVKYGQCWVFASVMCTVMRCLGVPTRVVSNFHSAHNTDGNLTIDTYYDRTAE
MLAQRPDKIWNFHVWNECWMIRKDLPPGYNGWQVLDPTPQQTSSGLFCCGPASVKAIKEGD
VQLPYDTPFVYAEVNADEVVWLFEGGQVQEILAHNTSYIGKAISTKMVGSDERLDITSSYK
YPEGSSEERSAFIKASWKLLEPRRNASNLKAKPVRLQLHVRAPMWGQDLPLMLYVQRVPVR
DHGLTVRFCAQALLHGGGTRKPFWRHIVHLNVDFEKELQWPFLLPYSSYRDKLTDERLIRV
AGIAEVETGQFILVLKDFSLEPPHLSIEVFERAEVGKALDIHITLTNTLMVPLNNCTMVLE
GSGLINQMTKDLGTLMAGHTIRIHLELYPIKAGPHQLQVLISSSEVKEIKGYKDILVAA
>mouse_F13A1
LNVTAVHLFKDSNKIDHHTDKYDNNKLIVRRGQTFYIQIDFNRPYDPRDLFRVEYVIGRYP
QENKGTYIPVPVVKLQSGKWGAKVIMNEDRSVRLSVQSSPECIVGKFRMYVAVWTPTRRDP
ETDTYILFNPWCEEDAVYLDDEKEREEYVLNDIGVIFYGDFKDIKSRSWSYGQFEDGILDT
CLYVMDKAEMDLSGRGNPIKVSRVGSAMVNAKDDEGVLVGSWDNVYAYGIPPSAWTGSVDI
LLEYRSSETPVRYGQCWVFAGVFNTFLRCLGIPARVITNYFSAHDNDANLQMDIFLEEDGN
VSKLTKDSVWNYHCWNEAWMTRPDLPVGFGGWQAVDSTPQENSDGMYRCGPASVQAVKHGH
VCFQFDAPFVFAEVNSDLVYITAKQDGTHVVEAVDATHIGKLIVTKQIGGDGMQDITDTYK
FQEGQEEERLALETALMYGAKKTLNTEVKSRSDVTMNFDVENAVLGKDFKVTITFQNNSSN
LYTILAYLSGNITFYTGVSKKEFKKESFEETLDPFSSKKKEVLVRAGEYMSHLLEQGFLHF
FVTARIESRDVLAKQKSIILTIPKITIKVRGAAMVGSDMVVTVEFTNPLKETLQNVWIHLD
GPGVMRPKRKVFREIRPNTTVQWEEVCRPWVSGHRKLIASMTSDSLRHVYGELDLQIQR
>mouse_EPB42
LSIKSCDFHAAENNEEHYTKAISSQHLTLRRGQSFTITLNFRAPFLSAKKVALIAQTGEQP
SKINKTQAIFPISSGDQKGWSAAVEERDAQHWTVSVTTPVDAVIGHYSLLLQVSGK-
KQYPLGQFTLLFNPWNRDDAVFLQNEAERTEYVLNQNGFIYLGTADCIQEEPWDFGQFEKD
VMDLSLKLLSM-
DKQVKDWNQPAHVARVVGALLHALKKKSVLPISQTQAAQEGALLYKRRGSVPILRQWLTGQ
RAVYETQAWVSAAVACTVLRCLGIPARVVTTFDSAQGTVGSLLVDEYYNEEGLQNEGQRGH
IWVFQTSVECWMNRPDLSQGYGGWQILHPRAPNGAGVLGSCSLVPVRAVKEGELQLDPAVP
ELFAAVNASCVVWKCCEDGKLELTNSNRKDVGNCISTKVVGSDRCEDITQNYKYPAGSLQE
KEVLEKVQKERLKLGKDNGCEPWDPLHMFFESSSIPLSGDGQLSVTLINPTDEEKKVHLVI
GAQALYYNGVLAAGLWSKKQLFMLKPNQVMRLSTNLSFSCFEQTPPENSFLRVTAMARYHT
SLSCFAQENMAIGKPDLIIEMPKRAAQYRPLTVSVRMHNSLEAPMQNCIISIFGRGLIHEK
RYGLGSLWPGSSLHTQFQFTPTHLGLQRLTVEVDCDMFQNLTGYRSVLVVA
>chicken_TGM1
LVPRGLVVGSRADRIAHHTAEFCSPQLVVRRGQPFHLRVLLPRPFDPEDSLCVELLLGPTP
QVAKGTHVLIPLGETSATGWTAEEAGSGSPALRLRLSAPADAPIGRYRLSVKTRTGAPFDD
RNDVIVLFNPWCEEDGVYMEQTNDLNEYVLNETGRIFYGTEEQIAERSWNYGQFDAGVLDA
CLAILDRRRMPHSARGDPVMVTRVVSAMVNSLDDNGVLVGNWTGDYTQGTNPSAWAGSVDI
LRSYHRGGAPVRYGQCWVFAGVVTTVLRCLGVPTRTVTNYNSAHDTDVSLTTDIYFDENMK
PLRLNTDSVWNFHVWNDCWMRRPDLPAGYDGWQVVDATPQETSSGLFCCGPCSVTAVKNGE
VFLKYDTAFVFAEVNSDKVYWQRKGNGAFAIVHVEEGAIGRRISTVGPQSAARIDITHLYK
HPEGSEAERRAVSTATSHGSRPRSGAA-
PSRGEVRLSLSSGPAVAGAELELKVTAHNAAPQPRTVRVRLSVCALRYTGVAAPPFRHEQH
RRVVAPGGEEQLCVAVPFSEYSPHVGSQDALRLTAAAAVETGEVVAKELRVRLAAPDLSMT

LLGPPVVGQEVSVQVLFRNPLPQKLTGAELRMEGAGLSCPASISVGTVAPEQTLRLRQPVV
PLRAGRRRLVAAMESAQLGPVHGELQFDAVP
>chicken_TGM2
LVLETCDLQCERNGREHRTAEMGSQQLVVRRGQPFTITLNFGRGYEEGDKLAFDVETGPCP
VETSGTRSHFTLTDPEEGTWSAVLQQQDGATLCVSLCSPSSARVGRYRLTLEASTGGSSFH
LGDFILLFNAWHPEDAVYLKEEDERREYVLSQQGLIYMGSRDYITSTPWNFGQFEDEILAI
CLEMLDINNLDCSRRNDPVYIGRVVSAMVNCNDDHGVLLGRWDNHYEDGMSPMAWIGSVDI
LKRWRRLGQPVKYGQCWVFAAVACTVMRCLGVPSRVVTNYNSAHDTNGNLVIDRYLSETGM
EERRSTDMIWNFHCWVECWMTRPDLAPGYDGWQALDPTPQEKSEGVYCCGPAPVKAIKEGD
LQVQYDIPFVFAEVNADVVYWIVQSDGEKKKS-
THSSVVGKNISTKSVGRDSREDITHTYKYPEGSEKEREVFSKAEHEKSSLG-----
EQEEGLHMRIKSEGANNGSDFDVFAFISNDTDKERECRLRLCARTASYNGEVGPQCFKDLL
NLSLQPHMEQSVPLRILYEQYGPNLTQDNMIKVVALLTEETGDSVVAIRDVYIQNPEIKIR
ILGEPMQERKLVAEIRLVNPLAEPLNNCIFVVEGAGLTEQRIEELEDVEPQAEAKFRMEFV
PRQAGLHKLMVDFESDKLTGVKGYRNVIIAP
>chicken_TGM3
AAQPSTDWHVKENGRDHHTSKFSSKELIVRRGQAFVITF--
NGVEQPEQTLTFIVETGPKPSKQAKTQATFGISSVSKDSWSAVLQSTSSHSVSVSISSPPN
AVIGRYKLSVQSTSTSSPETLGTFVLLFNPWSSGDNVFMPNKAECEEYVLEEFGIIFAGNN
YHINSFGWNFGQFQADILNICLSMLDRSATDVSHRHDPKYLGRVLSAMVNANDDQGVVLGN
WSGNYDGGKSPSSWTGSGEILQSWKKSGKPVRYGQCWVFAAVLTTVLRCLGIPTRPITNFS
SAHDADGNLRVDEFYDASGNHLNEGADSVWNFHVWNESWFSRSDLGPSYSGWQILDATPQE
ESGGIYQCGPASRNAVKEGDVDLDYDCPFVFAEVNADCMYWSYDRARKKTLLFNKSTVIGQ
LISTKAVGRDDRIDITSDYKYEEGSKKERDIFKKARKKLGLEDKFDPIEQKPDISGKFKAG
PLEVGKDLNLILVLKNLQSEVKSVNVNMTAWSTVYTRRPVREIWKDSLSVSLSPEEEKHFP
IKISYAEYQQQLTTDNAIEVTALCHVEGGIQVLVQRHIALDNPTIDIQVLGEAKVNEEMDV
EVVFTNPIDIEVKDCVLQVEGSDLVRILKIDVPPLKAKDISSTKFKLTPFETGSKHLLVNF
SCDKFADIKAFKTVKVID
>chicken_TGM4
LKVTKVDFLKSQNSVQHHTDAYNTSNLVVRRGQPFLLQLTLSRELRAADKLSLHFSIGERP
MEPTGTLMSLNPRSRNVSGWQIAIIKSSGTECTLSVTSAPNAAVGIYGLMVKTGPNIYKPE
KNTVYLLFNPWGEGDIVFLSNEAERKEYVLNDTGYIYVGSAFNIHSKPWNFGQFEESILDA
CMYLLDKSKLKMSSRRDPVVVSRAMSALVNANDDNGVVLGNWSGKYENGTSPMAWIGSVAI
LQQYYKTKKPVSYGQCWVFSGVLTTVMRCLGIPARSVSNFNSAHDTDENLRVDVYLNEKGE
KLWMSSDSVWNFHVWNDVWMKRKDLPSGFDGWQAIDATPQEQSQGTFQCGPCPLKAVKEGD
VYLPYDSKFVYAEVNADKVYWRVKEENKYTKLGVESQSIGANISTKAVGQNRREDITWQYK
FPEGSAEERASMKRAVSYLQPSGLTPRVVPKSGVQLEITEKPLCPGNPIEVTITVKSTVAG
SWTVDLASSCQLQSYTGKVHANLGYIKQTVKVEGQSEVHVPLKIMPDAYMKALADEEHVHV
TAIAEIGTPEKLTKEASLSFEYPPIQVQMPETAKVNNDFTCAFIFKNKLNVPLDNCKLMVE
GLGIFKMATFDEGDIQPGRIIKSEVICTPTRVGEKKIVARLTSNQVKDISVEKAITVTH
>chicken_TGM6
QKITKVDWHSKLNKAAHHTSDYNSTQIILRRGQAFAITLNFQTTVQPENNFTFIASTGPSP
AESQQTKAIFHLSEDAANGWSATQERCEPGRMSLMIVSPANAIIGRYKLKLQIASGTSSAL
LGQFVLLFNPWCPNDDVYMANEKERQEYVLNDSGIIFQGKEKYIQQEAWNYGQFEEDILDI
SLSILDRSSTDVSNRNDPIYVCRVISAMVNSNDEKGVVEGKWSGSYRSGTNPLHWSGSVSI
LRKWYRGRRPIRYGQCWVFAGVMCTVLRSLGIPTRVITNFNSAHDSNTNLSIDKYIDVSGK
TLDLTEDSIWNFHVWNESWFTRRDLGSFYDGWQVLDATPQEKSKGIYRCGPASTRAIKEGD
VNLDYDSSFVFAAVNADCVTWIQHSNKRKERIYSNTRKIGKFISTKAVGTNTRVDVTNNYK
YPEGSSKERQVYKKALKLLAVRSTGRRTTQKPTVSGKMKDAPPVIGQDILLTLALQNLTTD
FKTVNVKLRASAVLYTRKPKAEILQWSRSVQLGAEEVKEISFKITYTQYKNALLDDRKILV
TAVCEARQGASLLVEKDIVLQDPFLTIEVLGPTVVHKATNVLVTFTNPLSEVVTDCVLRAE
GSGLIKQLNINVARMAPMETATAEFEIVPYKSGIRQLQVDLACIHFSNIKGFVMLDVAP
>chicken_TGM6L
LKPTHISWQPSVNASAHHTDRYANTELTVRRGQPFTITLYFNRQKYPGESLAFVTEIGPSP
SESHRTRAVFNLSEVGASGWSAAQGPSESGYMTFIISSPANAIIGRYNLILQVNSGIFSRF
LGQFVLLFNPWCPGDDVYIANENERQEYVLNENGIIFVGNAKYIEARGWYYGQFQDHLLNI
CLTMLDLSAVDVSRRGDPKYVGRVISSMINGNDDNGVLLGKWQGSFHSHENPSRWDGSVVI
LQKWRQDNKPVQYGQCWVFAGVMCTVLRCLGIPTRLVSNFNSAHDVDRNLSIDKYYDSSGK
SLNISKDSTWDYHVWNESWFIRPDLGPRYNGWQVLDATPQEQSRGLFQCGPASVVAIKEGD
VDLDYDTLFVYTEVNADCNRWIVYNDGTKKRVYCDTEIIGRFISTKAVGSNSRVDITCNYK
YPEGSPEERRVYKKALAKIFGSHITEGAMRNPGISGKFKAEPPVFGKDINLILILNNLSTD
HKTVKVDISASSVLYTRRAVAEILKANTSVDLGSKQGKHIRLKIPYAYYGKYLTTDKRIQV

TALCEVMHGVKLLVEKTIILEDTNIIIKIPRRVVVNKAATLEISYANPLPEPVDRCVLLVT
---LMNQVKIHLARLAPRERSRIYFEFTPRRTGPLQLQVDFSCDKFSHVKGFVTIAVQP
>chicken_TGM5L
IELGDVNLNCPSNCQIHNTHFFGTDRQIIRRGQAFNFYASFNREWDDSDQATFTVETGLRP
CESNETKCTFPMGRLDQTCWSASYKTHQPKCINISVFPPSNACIGRYILNMQITSCTYQRC
LGDFYVLFNPWCADDPVYLDNQAQREEYILNEHGILYEGVHKHITSRPWHFGQFEDGILDI
CLKILDMGDRDHCWRNDPVHVSMVVNHMISSHTTNSIMKIPENNDYLKGTKPFSWNGSVPI
LQQWYNGRRPVRYGYCGSLASVMCTVMRCLGIPSRVVTNFCFPCSNENPLGINEIFDCTGK
NL-
CGKDKLWRYHCWNESWMARRDLNQCCGDWQCLDPTPLETGRGSACSGPTWVRSIREGDLDL
DYDGHSMFSRLNSNYAGWLSQNNAKKTKVFCDAWPCGQQLITKSVGSEQFEDITGNYKYEL
GSVKSKEAYYRAYRRIHPGYCNASFLSDSGINTRLKANCPMYGEDVQLHWLLENLRSEPKN
LKFNLCAQIITYNGCPMDQFWKDSVTVALGPREVKKIPLCVSYSQYGPYLSDHNIMKVVAV
SDPECGEVLMVSRDIVINRPPVIVKLLSQPRLKVPCTAEISFCNPLQEDMKNCIMTLEGCG
LFKPMTIDLGTLASNQQARTIVEFTPYRLGSHRLLANLGCHKFAYCKGCAKAEVCN
>chicken_F13A1
LRVVDIHMFKEINKQQHHTDKYYNPKLIVRRGQPFQIQIDFSRPYDPEDQIWLEYLIGRYP
QPNKGTYIPILIGDLKPGEWGAKITHRENNSIRLSIMSSATCIIGKFRLYIAIWTPTHRNS
ATDTYILFNPWCQLDAVYLDDEKEREEYVLNDVGIVFHGDVNEVKLRSWSYGQFEENILDA
CLFLMDKAELELSGRGNPIKICRVASAMINSKDDNGVLAGSWDNLYDYGVPPSAWTGSVDI
LLEYYSSKQPVRYGQCWVFAGVFNTFLRCLGIPARLITNYSSAHDNNANLQLDFFLDDEGQ
VDRLTKDSVWNYHCWNEAWMTRPDLPVGFGGWQAVDGTPQETSDGMYRCGPASVQAIKHGH
VCFQFDAPFVYAEVNSDIIYSRMGKNGSQVIEKIDTTHIGKLIVTKGVGNDDMVDITENYK
FQEGSAEERLALETAVMYGVKKQTTQPYQPQKDIEMDLQVQKVVLGSDFKVTIILRNKSRN
SYTATTYLSGNIVFYTGVTKSEFKKHSFSAKLEPLLSNTFDVMITSAEYLNDLLDQASFHF
FVTARIETGKVLAMQKAVVLEIPTLKIKTKGQMVVDRETSVVVEFTNPLKQTLENATLRLE
GPGVLRTMKKEFRQIPAMSTLIWDVKCIPKRPGLRKLIASLNCDALRHVYGELNIQVQK
>chicken_EPB42
LSAKKCDLKIIMNNNNHHTEEISTERLIVRRGQPFVITVNFSSPYLKQKRIFLSVQTGPQR
SKADGTQVKFPISSGDQKQWSARLEEQDPYFWTISVNTPANASIGQYDLFLHASKA--
CRLLGKFILLFNPWCRDDEVFLPNEAQRQEYILNQDGVIYSGTENEILAQPWDFRQFDVGI
VDICFKLLDIGDQDHTQRKNPIYICRTVAAMMNCNL-
ARILSESGTRECYDGTPPSKWLGSNAILQQWAALQRPVRYGQCWVFAAVMCSVLRCLGIPT
RVVTGFTWAHNTNSNPNVNEYYEEDGTLLPDKSARVWTFHVWNECWMARTDLLPEYSGWQA
LDATCQKKSKGPSFCGPAPVQAIKEGDVEVDYDVCYFFAAMNAKCKVWIHMADDIFKPASI
CTKYMGNNISTKSVGSERCEDITHNYKYPEGSLQEKKVLDKVYRKTQTLQAISSLEEKVNL
FIDLQKSSLILGQDIPLSIEVFNYSDREKATDLVLGVQSLHYNGVPIMQLWKEKLNFIIKS
NEVRTLQVFVPYSQYGKELGKNRLLRLTATLRDEDSYIYFAQEEMSICDPPLTIEFPDNVL
LYQPATVKISLLNPLAEPLEKCVIVVGGQGLIYQRKYRLGTVQPKSTQDLNISFTPTESGP
RRLTAHLTCLQIQNLKSYKTINVAA
>human_TGM1
LVVNGVDLLSDQNRREHHTDEYEYDELIVRRGQPFHMLLLLSRTYESSDRITLELLIGNNP
EVGKGTHVIIPVGKGGSGGWKAQVVKASGQNLNLRVHTSPNAIIGKFQFTVRTQSDLPFDP
RNEIYILFNPWCPEDIVYVDHEDWRQEYVLNESGRIYYGTEAQIGERTWNYGQFDHGVLDA
CLYILDRRGMPYGGRGDPVNVSRVISAMVNSLDDNGVLIGNWSGDYSRGTNPSAWVGSVEI
LLSYLRTGYSVPYGQCWVFAGVTTTVLRCLGLATRTVTNFNSAHDTDTSLTMDIYFDENMK
PLHLNHDSVWNFHVWNDCWMKRPDLPSGFDGWQVVDATPQETSSGIFCCGPCSVESIKNGL
VYMKYDTPFIFAEVNSDKVYWQRQDDGSFKIVYVEEKAIGTLIVTKAISSNMREDITYLYK
HPEGSDAERKAVETAAAHGSKPNVYANRGSAEDVAMQVEAQDAVMGQDLMVSVMLINHSSS
RRTVKLHLYLSVTFYTGVSGTIFKETKKEVELAPGASDRVTMPVAYKEYRPHLVDQGAMLL
NVSGHVESGQVLAKQHTFRLRTPDLSLTLLGAAVVGQECEVQIVFKNPLPVTLTNVVFRLE
GSGLQRPKILNVGDIGGNETVTLRQSFVPVRPGPRQLIASLDSPQLSQVHGVIQVDVAP
>human_TGM2
LVLERCDLELETNGRDHHTADLCREKLVVRRGQPFWLTLHFGRNYEASDSLTFSVVTGPAP
SQEAGTKARFPLRDVEEGDWTATVVDQQDCTLSLQLTTPANAPIGLYRLSLEASTGGSSFV
LGHFILLFNAWCPADAVYLDSEEERQEYVLTQQGFIYQGSAKFIKNIPWNFGQFEDGILDI
CLILLDVNGRDCSRRSSPVYVGRVVSGMVNCNDDQGVLLGRWDNNYGDGVSPMSWIGSVDI
LRRWKNHGQRVKYGQCWVFAAVACTVLRCLGIPTRVVTNYNSAHDQNSNLLIEYFRNEFGE
IQGDKSEMIWNFHCWVESWMTRPDLQPGYEGWQALDPTPQEKSEGTYCCGPVPVRAIKEGD
LSTKYDAPFVFAEVNADVVDWIQQDDGSVHKSINRSLIVGLKISTKSVGRDEREDITHTYK
YPEGSSEEREAFTRANHLNKLAE-----
KEETGMAMRIRGQSMNMGSDFDVFAHITNNTAEEYVCRLLLCARTVSYNGILGPECTKYLL
NLNLEPFSEKSVPLCILYEKYRDCLTESNLIKVRALLVEVINSYLLAERDLYLENPEIKIR

ILGEPKQKRKLVAEVSLQNPLPVALEGCTFTVEGAGLTEQKTVEIPDVEAGEEVKVRMDLL
PLHMGLHKLVVNFESDKLKAVKGFRNVIIGP
>human_TGM3
LGVQSINWQKAFNRQAHHTDKFSSQELILRRGQNFQVLMIMNKGLGSNERLEFIVSTGPYP
SESAMTKAVFPLSNGSSGGWSAVLQASNGNTLTISISSPASAPIGRYTMALQIFSQISSVK
LGTFILLFNPWLNVDSVFMGNHAEREEYVQEDAGIIFVGSTNRIGMIGWNFGQFEEDILSI
CLSILDRSATDVASRNDPKYVGRVLSAMINSNDDNGVLAGNWSGTYTGGRDPRSWNGSVEI
LKNWKKSGSPVRYGQCWVFAGTLNTALRSLGIPSRVITNFNSAHDTDRNLSVDVYYDPMGN
PLDKGSDSVWNFHVWNEGWFVRSDLGPSYGGWQVLDATPQERSQGVFQCGPASVIGVREGD
VQLNFDMPFIFAEVNADRITWLYDNTGKQWKNSVNSHTIGRYISTKAVGSNARMDVTDKYK
YPEGSDQERQVFQKALGKLKPNTPFAAEEQEPSIIGKLKAGMLAVGKEVNLVLLLKNLSRD
TKTVTVNMTAWTIIYNGTLVHEVWKDSATMSLDPEEEAEHPIKISYAQYEKYLKSDNMIRI
TAVCKVPDESEVVVERDIILDNPTLTLEVLNEARVRKPVNVQMLFSNPLDEPVRDCVLMVE
GSGLLLNLKIDVPTLGPKERSRVRFDILPSRSGTKQLLADFSCNKFPAIKAMLSIDVAE
>human_TGM4
LQVLHIDFLNQDNAVSHHTWEFQTSSPVFRRGQVFHLRLVLNQPLQSYHQLKLEFSTGPNP
SIAKHTLVVLDPRTSDHYNWQATLQNESGKEVTVAVTSSPNAILGKYQLNVKTGNHILKSE
ENILYLLFNPWCKEDMVFMPDEDERKEYILNDTGCHYVGAARSIKCKPWNFGQFEKNVLDC
CISLLTESSLKPTDRRDPVLVCRAMCAMMSFEKGQGVLIGNWTGDYEGGTAPYKWTGSAPI
LQQYYNTKQAVCFGQCWVFAGILTTVLRALGIPARSVTGFDSAHDTERNLTVDTYVNENGK
KISMTHDSVWNFHVWTDAWMKRPDLPKGYDGWQAVDATPQERSQGVFCCGPSPLTAIRKGD
IFIVYDTRFVFSEVNGDRLIWLVKMVEELHVISMETTSIGKNISTKAVGQDRRRDITYEYK
YPEGSSEERQVMDHAFLLLSSEREHRRPVKENFLHMSVQSDDVLLGNSVNFTVILKRKTAA
LQNVNILGSFELQLYTGKKMAKLCDLNKTSQIQGQVS-
EVTLTLDSKTYINSLADEPVIRGFIIAEIESKEIMASEVFTSFQYPEFSIELPNTGRIGQL
LVCNCIFKNTLAIPLTDVKFSLESLGISSLQTSDHGTVQPGETIQSQIKCTPIKTGPKKFI
VKLSSKQVKEINAQKIVLITK
>human_TGM5
LEVALTDLQSSRNNVRHHTEEITVDHLLVRRGQAFNLTLYFNRSFQPGDNIIFVVETGPLS
DLALGTRAVFSLARHSPSPWIAWLETNGATSTEVSLCAPPTAAVGRYLLKIHIDSFVTAYQ
LGEFILLFNPWCPEDAVYLDSEPQRQEYVMNDYGFIYQGSKNWIRPCPWNYGQFEDKIIDI
CLKLLDKSATDCALRGSPVYVSRVVCAMINSNDDNGVLNGNWSENYTDGANPAEWTGSVAI
LKQWNATGQPVRYGQCWVFAAVMCTVMRCLGIPTRVITNFDSGHDTDGNLIIDEYYDNTGR
ILNKKKDTIWNFHVWNECWMARKDLPPAYGGWQVLDATPQEMSNGVYCCGPASVRAIKEGE
VDLNYDTPFVFSMVNADCMSWLVQG-
GKEQKLHQDTSSVGNFISTKSIQSDERDDITENYKYEEGSLQERQVFLKALQKLKARSFHG
SPSDVVQVSLKFKLDPPNMGQDICFVLLALNMSSQFKDLKVNLSAQSLLHDGSPLSPFWQD
TAFITLSPKEAKTYPCKISYSQYSQYLSTDKLIRISALGEESSPEKILVNKIITLSYPSIT
INVLGAAVVNQPLSIQVIFSNPLSEQVEDCVLTVEGSGLFKQQKVFLGVLKPQHQASIILE
TVPFKSGQRQIQANMRSNKFKDIKGYRNVYVDF
>human_TGM6
IRVTKVDWQRSRNGAAHHTQEYPCPELVVRRGQSFSLTLELSRALDCEEILIFTVETGPRA
SEALHTKAVFQTSEERGEGWTAAREAQMEKTLTVSLASPPSAVIGRYLLSIRLSSHHSNRR
LGEFVLLFNPWCAEDDVFLASEEERQEYVLSDSGIIFRGVEKHIRAQGWNYGQFEEDILNI
CLSILDRSATDVSCRHNPIYVTRVISAMVNSNNDRGVVQGQWQGKYGGGTSPLHWRGSVAI
LQKWLKGRKPVKYGQCWVFAGVLCTVLRCLGIATRVVSNFNSAHDTDQNLSVDKYVDSFGR
TLDLTEDSMWNFHVWNESWFARQDLGPSYNGWQVLDATPQEESEGVFRCGPASVTAIREGD
VHLAHDGPFVFAEVNADYITWLWHEDESRERVYSNTKKIGRCISTKAVGSDSRVDITDLYK
YPEGSRKERQVYSKAVNRLFGVEASGRPATKPSIAGKFKLEPPMLGHDLRLALCLANLTSR
AQRVRVNLSGATILYTRKPVAEILHESHAVRLGPQEEKRIPITISYSKYKEDLTEDKKILL
AAMCLVTKGEKLLVEKDITLED-
FITIKVLGPAMVGVAVTVEVTVVNPLIERVKDCALMVEGSGLLQQLSIDVPTLEPQERASV
QFDITPSKSGPRQLQVDLVSPHFPDIKGFVIVHVAT
>human_TGM7
LRLESVDLQSSRNNKEHHTQEMGVKRLTVRRGQPFYLRLSFSRPFQSQDHITFVAETGPKP
SELLGTRATFFLTRQPGNVWSASDFTIDSNSLQVSLFTPANAVIGHYTLKIEISQGSVTYP
LGTFILLFNPWSPEDDVYLPSEILLQEYIMRDYGFVYKGHERFITSWPWNYGQFEEDIIDI
CFEILNKSAKDCSQRNDVVYVCRVVSAMINSNDDNGVLQGNWGEDYSKGVSPLEWKGSVAI
LQQWSARGQPVKYGQCWVFASVMCTVMRCLGVPTRVVSNFRSAHNVDRNLTIDTYYDRNAE
MLTQKRDKIWNFHVWNECWMIRKDLPPGYNGWQVLDPTPQQTSSGLFCCGPASVKAIREGD
VHLAYDTPFVYAEVNADEVIWLLGDGQAQEILAHNTSSIGKEISTKMVGSDQRQSITSSYK
YPEGSPEERAVFMKASRKMLGPQRASLGLRDQPAQLQLHARIPEWGQDLQLLLRIQRVPDS
THGLVVRFCAQALLHGGGTQKPFWRHTVRMNLDFGKETQWPLLLPYSNYRNKLTDEKLIRV

SGIAEVETGRSMLVLKDICLEPPHLSIEVSERAEVGKALRVHVTLTNTLMVALSSCTMVLE
GSGLINQIAKDLGTLVAGHTLQIQLDLYPTKAGPRQLQVLISSNEVKEIKGYKDIFVTV
>human_F13A1
LNVTSVHLFKDTNKVDHHTDKYENNKLIVRRGQSFYVQIDFSRPYDPRDLFRVEYVIGRYP
QENKGTYIPVPIVSLQSGKWGAKIVMREDRSVRLSIQSSPKCIVGKFRMYVAVWTPTSRNP
ETDTYILFNPWCEDDAVYLDNEKEREEYVLNDIGVIFYGEVNDIKTRSWSYGQFEDGILDT
CLYVMDRAQMDLSGRGNPIKVSRVGSAMVNAKDDEGVLVGSWDNIYAYGVPPSAWTGSVDI
LLEYRSSENPVRYGQCWVFAGVFNTFLRCLGIPARIVTNYFSAHDNDANLQMDIFLEEDGN
VNKLTKDSVWNYHCWNEAWMTRPDLPVGFGGWQAVDSTPQENSDGMYRCGPASVQAIKHGH
VCFQFDAPFVFAEVNSDLIYITAKKDGTHVVENVDATHIGKLIVTKQIGGDGMMDITDTYK
FQEGQEEERLALETALMYGAKKPLNTEMKSRSNVDMDFEVENAVLGKDFKLSITFRNNSHN
RYTITAYLSANITFYTGVPKAEFKKETFDVTLEPLSFKKEAVLIQAGEYMGQLLEQASLHF
FVTARIETRDVLAKQKSTVLTIPEIIIKVRGTQVVGSDMTVTVEFTNPLKETLRNVWVHLD
GPGVTRPMKKMFREIRPNSTVQWEEVCRPWVSGHRKLIASMSSDSLRHVYGELDVQIQR
>human_EPB42
LGIKSCDFQAARNNEEHHTKALSSRRLFVRRGQPFTIILYFRAPFLPAKKVALTAQTGEQP
SKINRTQATFPISSGDRKWWSAVVEERDAQSWTISVTTPADAVIGHYSLLLQVSGR-
KQLLLGQFTLLFNPWNREDAVFLKNEAQRMEYLLNQNGLIYLGTADCIQAESWDFGQFEGD
VIDLSLRLLSK-
DKQVEKWSQPVHVARVLGALLHFLKEQRVLPTPQTQATQEGALLNKRRGSVPILRQWLTGR
RPVYDGQAWVLAAVACTVLRCLGIPARVVTTFASAQGTGGRLLIDEYYNEEGLQNEGQRGR
IWIFQTSTECWMTRPALPQGYDGWQILHPSAPNGGGVLGSCDLVPVRAVKEGTLGLTPAVS
DLFAAINASCVVWKCCEDGTLELTDSNTKYVGNNISTKGVGSDRCEDITQNYKYPEGSLQE
KEVLERVEKEKMEREKDNGLETASPLYLLLKPSSLPLRGDAQISVTLVNHSEQEKAVQLAI
GVQAVHYNGVLAAKLWRKKLHLTLSANLEKIITIGLFFSNFERNPPENTFLRLTAMATHES
NLSCFAQEDIAICRPHLAIKMPEKAEQYQPLTASVSLQNSLDAPMEDCVISILGRGLIHER
SYRFRSVWPENTMCAKFQFTPTHVGLQRLTVEVDCNMFQNLTNYKSVTVVA
>zebra_finch_TGM1
LAPVWLRPGSSTNRTAHHTQEFPARPLVVRRGQRFHVGMRLPREPRTGDGICLELTLGPNP
QVAKGTHVLVPLGGSSPTGWSAEVDENSGHALWVGLTAPPTAPIGRYRLSARTRTEAPFEP
DNDLVLLFNPWCQEDSVYMEQTSDLSEYVLNESGRIFYGTEEQIAERAWNYGQFEPGVLDA
CLFILDRRGMPHGARGDPVMVARVVSAMVNSLDDSGVLVGNWTGDYSQGTNPSAWAGSVGI
LRSFHGTGSPVRYGQCWVFAGVVTTVLRCLGLPTRTVTNYNSAHDTDTSLTTDIYLDEAMR
PLRLNTDSVWNFHVWNDCWMKRPDLPEGYDGWQVVDATPQETSSGLFCCGPCSVKAVKNGD
VFLKYDTPFVFAEVNSDKVYWQRQAHGGFAVVHVEEGAIGRRISTLGATGHARVDITDQYK
HPEGSEEERRAVSAATSHGSRPRQRGP-
TSGGEVTMTVGSGPAVAGADLELRAVLKNGGLEPRTLRLRFSLCAVRYTGVAGAAFRQEQH
RRTLPPGQEDTVTMTVTYTEYQPHVGDQDALKLTVAAAVESGQVLAKELLVRLHTPELTLT
LLGPAVVGRPVSVQVVFQNPLPAPLPSASLRMEGAGIACPKPLALGSLGAGQTLRLSQSVT
PLRAGQRRLVATLESAALPPLSGCVQFSAQT
>zebra_finch_TGM2
LVLETWDLHCDRNGREHRTAAMGCGQLVVRRGQPFAITLRFGRAYDEADKLSFNVETGPFP
SETSGTSSHFVMTDPEESCWSAVIQQQEGESLSVSLCSPANACIGRYRLTLETCTDGSSYQ
IGDFILLFNPWHPEDTVFMRDENERREYVLSQDGLIYQGTCDYIYSIPWNFGQFEDEVLAI
CLNMLDINNQDCSRRNDPVYIGRVVSAMVNCNDDRGVLFGRWDNCYDDGMSPMAWIGSVDI
LKRWKKFGQPVKYGQCWVFAAVACTVMRCLGIPSRVVTNYNSAHDTNANLTIDRYVNENGE
QERQSWDKIWNFHCWLESWMARPDLAAGYDGWQVLDPTPQEKSEGIYCCGPAPVKAIKEGD
MLLKYDIPFIFAEVNADVVYWVVQGDGMQKQS-
IRSSDVGKNISTKSIGRDSREDITHNYKYPEGSEEERAVFEKAEHQKKSIG-----
EEDEGLHLRIKSEGANKGSDFDVFAVVTNNTDEERVCRVTLCARAASHNGTLGPQCLTDQV
DMDIEPRAEKRVPLRILYEEYRDKLTQDNLIKVVALLKDQTGDIIVAVRDIYVENPPIKIR
ILGEPMQNRKLVAEISLVNPLTVPLNGCVFVVEGTGLTEQLVKELEEVEPQAEAKFRMDLM
PRLTGLQKLMVDFESDHLTGVKGYRNVIIAP
>zebra_finch_TGM3
ATQPRTDWHLKENAREHHTSKFSSEELIVRRGQAFTITF--
NGTERPEQNLIFIAETGPKPSKATKTLATFDISSVSKEGWSAVLQSSSSSSVSISISSPHN
AVIGRYRLSVQS---
SSPHNLGTFVLLFNPWSSGDDVFMPNKAECEEYVLEEFGIIFAGNKNHINSFGWNFGQFQG
DILNICLSIMDRSLTDVAHRHDPRYLGRVLSAMVNANDDQGVVLGNWSGKYEGGKNPTSWT
GSGEILQSWKKSGRPVKYGQCWVFAAVLTTVLRCLGIPTRTITNFSSAHDADGNLRVDEFY
DADGNHLERGADSVWNFHVWNESWFTRNDLGPSYSGWQVLDATPQEESGGIYQCGPTSRHA
VKEGEVELDYDGPFVFAEVNADCMYWNYDSAGKKTLIFSKSTEIGASISTKAVGRDDRVDV
TRDYKYEEGSAKEREIFKKARKKLGLEDKFDPVEQKPDISGKFKAGSLEVGKDVNLLLVLE

NLQSDAKTVHVNMTAWSTLYTRRRVNQIWKDSISVTLAPKEEKQFPIKIKYPEYQQQLTTD
KTIQVTALCHVEDGMQVLVKRNITLDNPAIDIQVLGEAKVNKEVFVEVTFTNPIDEEMKDC
MLQVEGSDLLQILELRIPPLKASEKSSTKFKLIPSEAGPKHLLVNFSCDKFADIKTFKMVN
VIN
>zebra_finch_TGM4
LKVTEIDFLKRQNTQQHHTDGYNIKNLVVRRGKPFQVQVSLSRELRAADKLSLSFSIGENP
MKNRGSLLSLKPEREDPNGWKVSVVKKNGNECLLSVTSSPNAPVGKYNLHVKTGTNIYTPE
NGVIYLLFNPWCEDDAVYMADEAEKEEYVLNDTGYIYVGSAYSIYDRPWNFGQFEEFVLDA
CMYLLDKSKLKLTERRDPAHVSRAMSALVNANDDSGVLLGNWSGNYANGTSPMDWIGSVTI
LQQYYKTKKPVRYGQCWVFAGVLNTVMRCLGVPCRCVSTFDSAHDTEENLTVDIYLNEKGE
KLSLSFDSVWNFHVWNDAWMKRTDMPNGFSGWQAIDSTPQEQSQGRFQCGPCPVKAVREGD
VYLPYDAKFVYAEVNADRVYWVVKKVDKYFKVGTETQAIGKNISTKAVGQNRREDITREYK
YPEGSKEERMSMQRAYSFIRPSGLMPRPVTETGLNLEITTEPLHPGNPLELAITVKISAPG
NWTVNLTGSCQLQYYTGNVQANLGTIKETINLEGPSEMQIPLKIAPDAYMKTLSDEVLILV
TAIAEVETNDKLTKETSMRFEYPPITVQMPETAKLYNDFNCAFIFKNKLNVTLENCKLLVE
GLGIFKMTTFDLGDIMPGKIVKSEITCTPTRLGEKKIVAKLISNQIKGISAEKGIFITE
>zebra_finch_TGM6
LKIGKVNWQSKLNKAAHHTSDYSSTELILRRGQPFNISLNLKTTAQSWDNLTFIASTGPSP
AESQQTKAIFALSEEGANGWSATQEPSEPRCLNFTILSPADAVIGRYKLQLQVLAGVSSKV
LGQFVLLFNPWCPDDDVYMADEKERQEYVLNDSGIIFQGLEKDIQEEAWNYGQFEEDILDI
SLAVLDRSAVDVSKRNSPVYVSRVISAMVNSNDEKGVVEGKWSGKFRSGTNPLRWSGSVAI
LRRWYRARRPVRYGQCWVFAGVTCTVLRCLGIPTRVITNFNSAHDKNLNLSVDKYIDVSGN
NLHLSEDSVWNFHVWNESWFIRRDLGSFYDGWQVLDATPQEKSKGIYQCGPASTRAIKEGD
VNLDYDSPFVFAAVNADCVTWIRYSKKRKERIYSDTRKIGKFISTKAVGTNSRVDVTANYK
YPEGSAKERQVYNKALKLLGVRRPGKRPAETPSVSGKLLDASPVVGQDIPLTLTLRNLTSD
FNTIKVKLKASAILYTRRPKAEILQLHRSVKLGSEEVKEISFKISYSQYKNSLMDDRKILV
TAVCQIKRGASLLVEKDIVLQDPFLTIKVLGPAVVHKPVSVEVTFTNPLCDEVTDCVLRAE
GSGLLKQLRIRVERMAPMETSTVKFEIIPYRSGTRQLQVDLVCTHFSDIKGFVMLQVAP
>zebra_finch_TGM5L
IELGEVDLNCPSNCQIHNTHFFGTDRQIIRRGQAFSFYARFNREWDDTDQAAFTVETGLRP
CESNETKCTFPMGRLDQTCWSASYKVHQPKCISISVFPPSNVCIGRYILNMQITSCTYQRC
LGDFYVLFNPWCADDPVYLDNQAQREEYVLNEHGILYEGVHKHITSRPWHFGQFEDGILDI
CLKILDMGDRDHCWRNDPVHVSMVVNHMISSHTTNSIMKIPENNDYLKGTKPFSWNGSVPI
LQQWYSGRRPVRYGYCGSLASVMCTVMRCLGIPSRVVTSFCFPCSMENPLGINEIFDSTGK
NL-
CGKDKLWRYHCWNESWMARRDINQCCGDWQCLDPTPLETGRGSACSGPTWVRSIREGELDL
DYDGHHMFSRVNSNYVGWLSQNNAKRTKFFCDTWPCGQRLITKSVGSEQFEDITGAYKYEL
GSVKNKEAYYRAYRRIHPGYCNASFLSDSGINMRLKANCPMYGEDVQLNWLLENLRNENKT
LKFNLCAQIITYSGCPMDQFWKDSVNVTLGPREVKKIPLCISYNQYGPYLCDHNIMKVVAV
SDPECGEILMVSRDIVVNRPPVIVKLLSQPRLKVPCTAEISFCNPLQEDMKNCVMTLEGCG
LFKPMTIDLGTLASNQQARTIVEFTPYRLGCHRLLANLGCHKFAYCKGCAKAEVCC
>zebra_finch_F13A1
LSVVDIYMFKEINKQEHHTDKYYNPKLIVRRGQAFQIQIDFNRPYKPEDQFWLEFLMGRYP
QQNKGTYIPILVGNLKPGQWGAKIIHRENNSIRLSIMSSTTCIIGKFRFYVAVMTPTRRNS
ATDTYILFNPWCQLDAVYLDDEKEREEYVLNDVGIVFHGNVEDVKSRSWSYGQFEENVLDA
CLFLMDKAELELSGRGNPIKICRVASAVINSRDDNGVIAGSWNNTYDYGVAPSAWTGSVDI
LLEYYSSKQPVRYGQCWVFAGVFNTFLRCLGIPARLITNYSSAHDNNANLRMDFFLDDEGK
VDRLTKDSVWNYHCWNEAWMTRPDLPVGFGGWQVVDGTPQETSDGMYRCGPASVQAIKHGH
VCFQFDAPFVYAEVNSDIFYTRMEKNGTQVVENIDTTHIGQLIVTKAVGGDGMVDITEQYK
FREGSDEERLALETAVMYGVKKQSIQDYQPQTDVDMDFQVQKAVLGNDFKVTITFRNKSRN
SYTATTYFSGNIVFYTGVTKTEFKKHSFSAKLEPSSSSSFDIVIKSSEYMSDLLEQASFHF
FVTARIETGKILAVQKAIMLEIPTLRIKTKGELVAGKETSVIVEFTNPLKQTLENVMLRLE
GPGVLRTIKKQFGRIPMNSTLTWEVKFTPMRPGLQKLIASLNSDALRHVYGELNIQIRK
>zebra_finch_EPB42
LSIQKCDFMIPENNKSHHTEEISTKRLIVRRGQPFTITVSFSVPFLKQKRTFLVVQTGPHS
SKADEIQAEFPISSGDQKQWSAAVEEQDPNFWTLCVNTPANAPIGQYTLLFRASKS--
PQLLGSFTLLFNPWCQDDEVFLANEAQRQEYILNQEGIIYQGTENAILAQPWEFSQFEEDM
LDICFILLALGEKDPAQRKNPVHVCRAVAAMVNCNE-
FRSLTEYEPGQHDNGTPPSTWLGSSPILQQWIASKQPVRYGRCWVFAAVLCSVLRCLGIPT
RVVTGFTWAHNTKCSLSVDEYYDEDGTLLQDKSAHVWTFHVWNECWMARSDLLPKYSGWQA
LDATCQEKSTGLSFCGPAPVHAIKEGDTQVDYDVCYFFAAINAKCHVWIHKADDTLKPAYG
GTKYTGNNISTKSVGSERCEDITQNYKYPEGSLQEKKVLDKAFRNMKELKTTSSLEEPLNL
FIHFQSSSLQLGQDITLSIEVFNNSDREKATHLVAGIQALHYNGVPIAQLWKEEFHFNLHS

```
NSVNNLRVSVPYTWFGKELGENHLLRLTAVLRDDSSYMYLAQEEISLCDSPLTIEFPENMV
QYEPSTAKISLQNPLMEPLEQCVIAVAGRGLIYQRNYRLGSVQAKSTQELQIPFTPTRAGP
RRLTARLTCLQLQNLKSYRTTNIAA
>fruit_fly_TGM
LGVLKVDLCLEDNHEEHHTSHFAKEALVVRRGEPFRLKIHFNRDYSPSDAISFIFTVATKP
SPGHGTLNALVPHDGDTLEWGAGIESHEGQTLTVLIKPPSTCPVTEWKLDIDTKLLRSYPL
PLPIYVLFNPWCPDDQVYLEDRDQRKEYVMHDTTLIWRGSYNRLRPSVWKIGQFERHVLEC
SLKVLGTVRIPPAYRGDPVRVARALSALVNSVDDDGVLLGNWSEDFSGGVAPTKWTGSVEI
LQQFYKTQKSVKFAQCWNFSGVLTTIARSLGIPSRIITCYSSAHDTQASLTVDVFIDANNK
KLAETTDSIWNYHVWNELWMQRPDLGVGFDGWQVVDATPQEASDNMYRVGPASVAAVKNGD
ILRPFDGGFVFAEVNADKLYWRYNGPQPLKLLRKDTLAIGHLISTKAVLKWEREDITDTYK
HAERSEEERSTMLKALKQSRHAFSRYYNDNFNDIEFDMEKDDIKIGQSFSVVLKVSNKSES
RTMATGQISCDAVLYTGVGAVEVKTLGFELELEPKSSDYVRMEVIFEEYYDKLSSQAAFQI
SAAAKVDTDYDYYAQDDFRVRKPDIKFQLGEAAIVQKELDVILRLENPLPIPLHKGVFTVE
GPGIEQPLKFKIAEIPVGGTAAATFKYTPPYAGRGTMLAKFTSKELDDVDGYRHYEIEP
```

Figure S2. Alignment of trimmed amino acid sequence of transglutaminases used for the phylogenetic analysis. The multiple sequence alignment is shown in fasta_aln format. Sequence regions that could not be aligned unambiguously were removed. GenBank accession numbers are listed in Table S1.

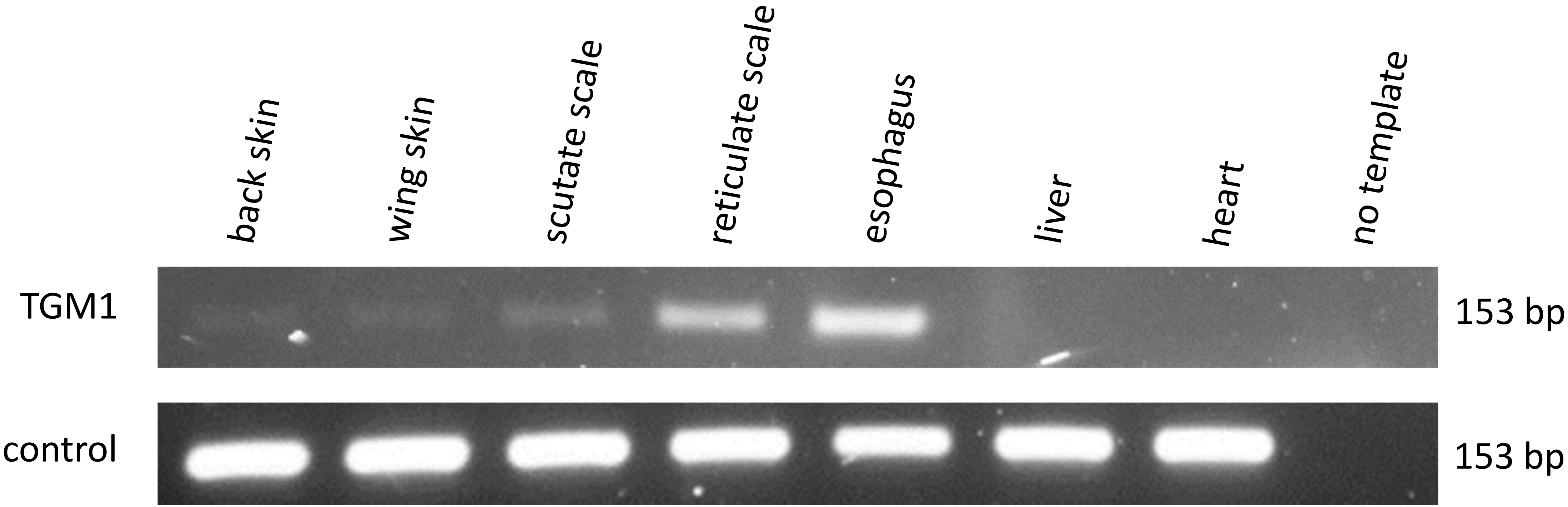

Figure S3. Expression of zebra finch *TGM1* in selected tissues. RNA from the indicated tissues and cultured cells was subjected to RT-PCR with primers specific for *TGM1* and the house-keeping gene *EEF1A1* (control). PCR products were analyzed by agarose gel electrophoresis, revealing bands that correspond to the predicted size of the PCR products. *TGM1* is expressed, to a small extent, in the skin (back skin and wing skin), and skin appendages (scutate scale and reticulate scale) of the zebra finch. The strongest expression of TGM1 was detected in the esophagus. *TGM1* is not expressed in the liver or the heart.

A

Chicken *TGM1*, last but one intron (partial), GenBank accession number JAENSL010000336.1, nucleotides 1-811

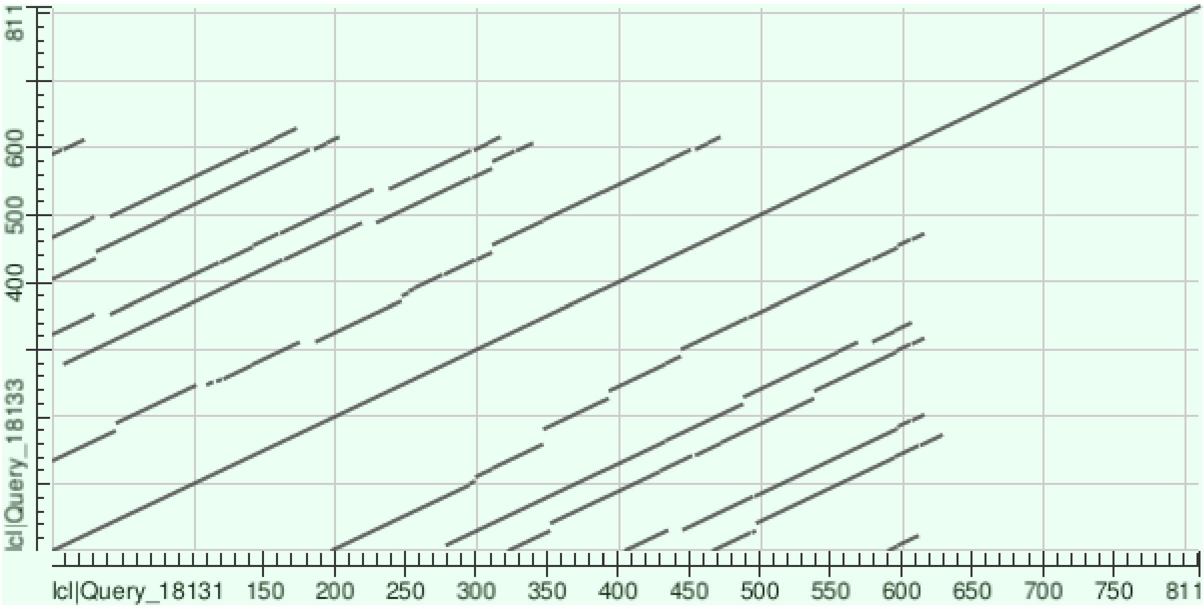

B

Chicken *TGM1*, last intron, GenBank accession number JAENSL010000336.1, nucleotides 958-1783

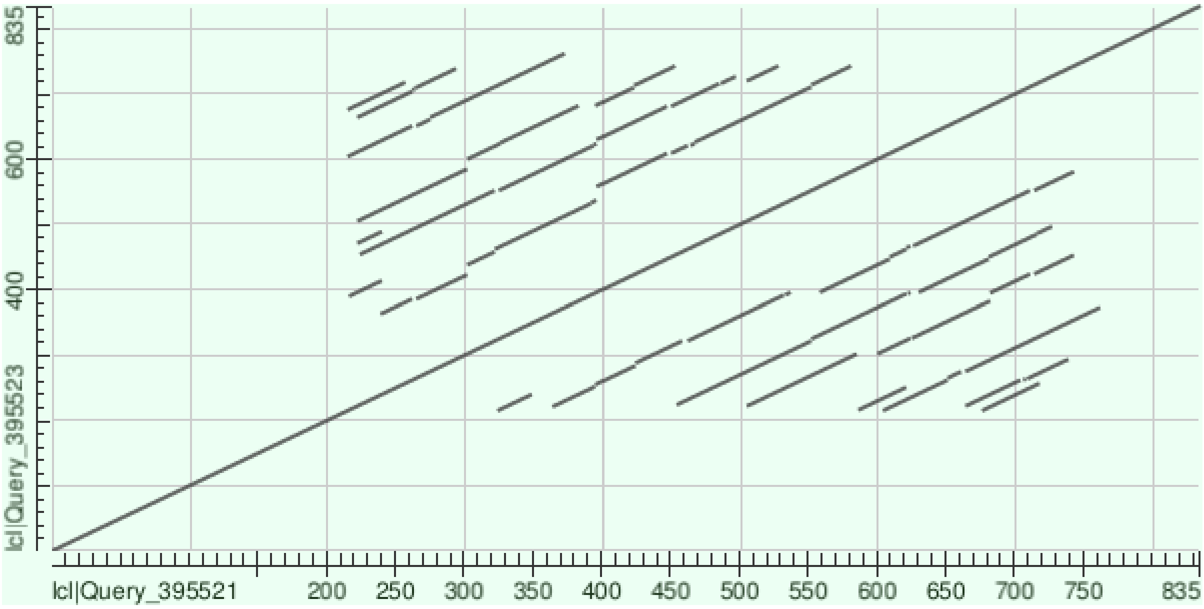

C

Zebra finch *TGM1* nucleotide sequence

```
>NC_054768.1:c998338-940558 Taeniopygia guttata isolate Blue55 chromosome 36, bTaeGut1.4.pri, whole genome
shotgun sequence
TTACGCTCCGCCGGCACTGGGAGTTACTGGGAGTTACTGGGAGCTACTGGGAGCCCCCGAGGAGGTACTGGGAGCACTGGGAGGGCGAACTGGGCAAACTGGGAGCATTGG
GAAGGGGTTACTGGGCAAACTGGGAGCACTGGGGGGTTACTGGGAGCGCTGGGAGGGTGAACTGGGCAAACTGGGAGCACTGGGAGGGGCAAATGGGGTAACTGGGAGCAC
TGGGGGGTTACTGGGAGCACTGGGAGGGGGTTACTGGGCAAACTGGGAGCACTGGGAGCACTGGGAGGGGGTTACTGGGCAAACTGGGCAAACTGGGAGCACTGGGAGGGG
GTTACTGGGCAAACTGGGAGCACTGGGAGGGGGTTACTGGGCAAACTGGGAGCACTGGGAGCACTGGGAGGGGTTACTGGGCATACTGGGAGCACTGGGAGGGGGTTACTG
GGCAAACTGGGAGTACTGGGAGCACTGGGAGGGGGTTACTGGGCATACTGGGAGCACTGGGAGGGGGTTACTGGGCATACTGGGAGCACTGGGAGGGGGTTACTGGGCAAA
CTGGGAGTACTGGGAGCACTGGGAGGGGGTTACTGGGCATACTGGGAGCACTGGGAGGGGGTTACTGGGCAAACTGGGAGCACTGGGAGGGGGTTACTGGGTAAACTGGGA
GCACTGGGAGCAATGGGAGGGGGTTACTGGTCATACTGGGAGCACTGGGAGGGGTGACTGGGGCTGCCGGAAGCGGGAACTGGGAGCACTGGGAGCACTGGGAGGGGTCAC
TGGGCAAACTGGGAGCACTGGGAGGGGTTACTGGGCAAACTGGGAGCACTGGGAGGGGTCACTGGGCAAACTGGGAGCACTGGGAGGCGTTACTGGGCAAACTGGGAGGGG
TTACTGGGAGAACTGGGAGAACTGGTCTGGCGGGGGAGGAGCTGCTGGGACAGGCCCGGCCCGGGGGGACTGGGAGGGACTGGGATGAACTGGGAGGGACTGGGAGGGACT
GGGAGGGACTGGGATCAAACTGGGAGGGACTGGGATCAAACTGGGAGGGACTGGGAGGGACTGGGATCAAACTGGGAGGGGTTACTGGGTAGGATTGGGGGTTACTGGTTT
ATACTGGGAGCACTGGGAGCCAAACTGGGTCATACTGGGACCACTGGGATCTATACTGGGAGCACTGGGAGCCATACTGGGAGCACTGGGAGCCAAACTGGGAATACTGGG
AGAGGGTTTGGGTCATACTGGTCCATACTGGGAGCACTGGGAGGGGTCACTGGTTCATACTGGGAGCACTGGGAGCCATACTGGGAATACTGGGAGAGGGTTGGGGCCATA
CTGGTCCATACTGGGAACACTGGGAGGGGTCACTGGTTCATACTGGGAGCACTGGGAGCCAAACTGGGAGCACTGGGAGGGGTCACTGGTTTATACTGGGAGCACTTAGAG
CCAAACTGGGAATACTGGTTTGTACTGGGAGCACTGGGAGGGGGTTTGGGGTCAAACTGGGAGCACTGGGAGCGGTTACTGGTTCATACTGGGAGCACTGGGAGCCATACT
GGGAATGCTGGGAGAGGGTTGGGGCCATACTGGTCCATACTGGGAGCACTGGGAGGGGTCACTGGTCCATACTGGGAGCACTGGGATGGGGTTTGGGTCATACTGGTCCAT
ACTGGGAGCACTGGGAGGGGTCACTGGTCCATACTGGGAGCACTGGGAGCCATACTGGGAGCGGTTACTGGTTCATACTGGGAGCACTGGGAGCGGTTACTGGTCCATACT
GGGAGCACTGGGAGCCATACTGGGAGCACTGGGAGAGGGTTTGGGTCATACTGGTCCATACTGGGAGCACTGGGATGGGGTTTGGGGTCATACTGGGAGCACTGGTTTGTA
CTGGGAGAGGGTTTGGGCCATACTGGTCCATACTGGGAGCACTGGGAGCCAAACTGGGAATACTGGGAGAGGGTTGGGGCCATACTGGGAGCACTGGTTTGTACTGGGAGA
GGGTTTGGGTCATACTGGTCCATACTGGGAGCACTGGGAGGGGTCACTGGTCCATACTGGGAGCACTGGGAACCATACTGGGAATACTGGGAGCACTGGGAGGGGTCACTG
GTTCATACTGGGAACACTGGGAGCCATACTGGGATTACTGGTTTGTACTGGGAGCACTGGGAGGGGGTTTGGGGTCAAACTGGGAGCGGTTACTGGTCCATACTGGGAGCG
CTGGGAGCCAAACTGGGAATACTGGGAGAGGGTTGGGGTCATACTGGTCCATACTGGGAGCACTGGGAGCCAAACTGGTCCATACTGGGAGCACTGGGAGGGGTCACTGGT
CCATACTGGGAGCACTGGGAGCCATACTGGTCCATACTGGGAGCACTGGGAGCTGTACTGGGAATACTGGTTTGTACTGGGAGCACTGGGAGGGGGTTTGGGGTCAAACTG
GGAGCACTGGGAGCGGTTACTGGTCCATACTGGGAGCACTGGGAGGGGTCACTGGACCATACTGGGAGCACTGGGAGCCATACTGGGAGCACTGGGAGAGGGTTTGGGTCA
TACTGGTCCATACTGGGAGCACTGGGAGCCATACTGGGAGCACTGGGAGCCATACTGGTCCATACTGGGAGCACTGGGTGGGGTCACTGGTTTATACTGGGAGCACTGGGA
GCCATACTGGGAATACTGGTTTGTACTGGGAGCACTGGGAGGGGGTTTGGGGTCAAACTGGGAGCACTGGGAGCAGTTACTGGTTCATACTGGGAGCACTGGGAGCGGTTA
CTGGTTCATACTGGGAGCACTGGGAGCCAAACTGGTCCATACTGGGAGCACTGGGAGGGGTCACTGGTCCATACTGGGAGCACTGGGAGGGGTCACTGGTCCATACTGGGA
ACACTGGGATGGGGTTTGGGGTCATACTGGGAGCACTGGTTTGTACTGGGAGAGGGTTTGGGCCATACTGGTCCATACTGGGAACACTGAGAGGGGTCACTGGTCCATACT
GGGAGCACTGGGAGCCAAACTGGTCCATACTGGGAGCACTGGGAGCCAAACTGGTCCATACTGGGAACACTGGGATGGGGTTTGGGGCCATACTGGGAGCACTGGTTTGTA
CTGGGAGAGGGTTTGGGCCATACTGGTCCATACTGGGAGCACTGGGAGGGGTTGTTGGGATATACTGGGAGATTTTGTACTGGTCTGTACTGGTTTATACTGGTTTATACT
GGTCTGTACTGGTGTGAACTGGTTTATACTGGTCTATACTGGTTTATACTGGTTTATACCAGTTCATACTGGTCTGTACTGGTTTATACTGGTTTATACTGGTCCATACTG
GTTTATACTGGTGTGAACTGGTTTATACTGGTGTGAACTGGTTTATACTGGTTTATACTGGTCCATACTGGTTTATACCAGTTCATACTGGTTTATACTGGTCCATACTGG
TCCATACTGGTCCATACCGGTCCATACTGGTTTATACTGGTTCATACTGGTCCGTACTGGTCCATACTGGTCCATACTGGTTTATACTGGTCCATACTGGTTTAAACTGGT
CCATACTGGTCCATACTGGTTTATACTGGTCCATACTGGTCCATACCAGTCCATACTGGTTTATACTGGTTCATACTGGTCCGTACTGGTCCATACTGGTCCATACTGGTT
TATACCGGTCCATACTGGTCCATACTGGTTTATCCTGGTCCATACTGGTCCATACTGGTTTATACCGGTCCATACTGGTCCATACTGGTTTATCCTGGTCCATACTGGTCC
ATACTGGTCCATACTGGTTTATACCAGCTCATACTGGTCCATACTGGTTTATACTGGTCCATACTGGTTTATACTGGTTTAAACTGGTCCATACTGGTCCATACTGGTTTA
TCCTGGTCCATACTGGTCCATACTGGTCCATACTGGTCCGTACTGGTCCATACTGGTCCATACTGGTCCATACTGGTTTATACTGGTCCATACTGGTCCATACTGGTCCAT
ACTGGTTTAAACTGGTCCGTACTGGTCCCCCAGGCCATGCCCGACCCCCGCCTGGACCGGGGCCGCTTTCCCTCCCTCCGCCTCACGGGGGCGCCGCGGGCCCCGCCCCCC
TCGGGCCGCCGCGCGGGGCTGTGGCGCCGCCTGGCGGCCGCGGGCGGGAACTGCGGCTGCTGCGGCTGCTGCGGGCGGAGCGGCGGCGGCGGCGGAGAGTGGGAACCGATC
CCGGGAGAGACGCCGGGACGGAGAACGGCGCAGCCAGTGCGGCCAGGTAAGGGTTAAACACCGGGGGGATTTTGGGGGGGCTCCGGTAACGGAGAGGTGCGGCCATGTTTG
GCTAAATGGGGTGGGGGGGAACGGGGAAACCGTGAGGGGGTTTGGGCGGGAAAATCGTGAGGGGAAATCGTGAGGGGATTTGGGCGGGAAAATCGTGAGGGGGTTTTAGAC
CCAAAAATGGGGATTTTAGAACCAAAAAAAAGGGGTTTTAAAGGGGGAAAATGGGGGTTTTTGGGTTTTTTTTTGGGGGGGGTTTGGGTTTTTTTTTTTGGGGTTTTTGG
TTGGGGGAAAAAAGGGGATTTCTGGGAAAAAAAATTGGGGTTTTTAGGCAAAAATTTGGGGATTATTGAGGGAAAAATGGGGTTTTAAAGGGGGAAAAAGGGGATTTTGGG
GGGTTTTTGGAGGGGGGAGGTTTGGGGTTTTCTTTTGGGGGGAAAAGGGGATTTGGGGAAGAAATTTGGGGGTTTGGGGGGAAAATGGGGATTTTAGAACCAAAAAAATGG
GGTTTTAAAGGGGGAAAATGGGGATGGGGGAGAACCGGGATGGGAAAACCGTGAGGGGATTTGGGCGGGAAAATCGTGAGGGGAAACCGTGAGGGGATTTGGGCGGGAAAC
CGTGAGGGGGTTTTAGAACCAAAAATGGGGATTTTTGAGGTAAAAAAATGAGGTTTTAAAGGGGGGAAAATGGGGATTTTTGGGGGGTTGTTTGGGGTTTTTGGTTGGGGA
TTTCTGGGAAAAAAATTGGGTTTTTTGGGCAAAAATTTGGGGATTTTTGAGGGAAAAATGGGGATTTTTGAGGGGAAAATGGGGGTTTTAGAGGGTTTTTTTAGGGAGGTT
TTGGGGTTTTCTTTTTTGGGGAAGAAATTCGGGGATTTTGGGAAGATATTTGGGGGTTTGGGGGGAAAATGGGGATTTTAGAGCCCAAAATGGGGATTTTAGAACCAAAAA
AATGGGGGTTTAAAGGGGGAAAATGGGGATGGGGGAGAACCGGGATGGGAAAACCGTGAGGGGATTTGGGCGGGAAAATCGTGAGGGGATTTCGGCGGGAAAATCGTGAGG
GGGATTTAGAGCCCAAAATGGGGATTTTTGAGGCAAAAAAATGGGGTTTTAAAGGGGGAAAATGGGGATATTTGGGGTTTTTTTGGGGGGTTGTTTGGGGGTTTTGGTTG
GGGGAGAAATGGGGGAAAAATTGGGGGGTTTTGGGCAAAAATTTGGGGATTTTTGAGGGAAAAATGGGGGTTTTTGAGGGAAAAATGGGGGTTTTAGAGGGGATTTTTTGG
GGTTTTATTTTTGGGGGAAAAAGGGGATTTGGGGAAGAAATTCGGGGGTTTGGGGGGAAAATGGGGATTTTAGAACTAAAAAGTGGGGTTTTAAAGGGGAAAAATAGGGAT
TTTTGCGGGGGGGATTTTAGGGTGGTTCTGGGGTTTTTTTGGGGTTTTTGGTTGGGGGGAAAAATGGGGATTTCTGGGAAAAAAATTGGGGTTTTTAGGCAAAAATTTGGG
GATTTTTGAGGGAAAATGGGGATTTTTGAGGGAAATATGGGGGTTTTAGAGGGGATTTTTTGGGGTTTTATTTTGGGGGGAAAAAGGGGATTTGGGGAAGAAATTTGGGGG
TTTGGGAGGAAAATGGGGATTTTAGAACCAAAAATATGGGGTTTTAAAAGGGGAAAATGGGGATTTTTGGGGTTTTTTGGGGGGTTGTTTGGGGTTTTTGGTTGGGAGAAA
AATGGGGATTTCTGGGAAAAAAATTTGGGGGTTTTGGGAAAAATTTGGGGATTTTTGAGGGAAAAATGGGGGTTTTAGAGGGGATTTTTTGGGAGGTTTTGGGGTTTTCTT
TTTTGGGGAAGAAATTCGGGGATTTTGGGAAGAAATTTGGGGGTTTGGGGGGAAAATGGGGATTTTAGAGCCCAAAATGGGGATTTTAGAACCCAAAAAATGGGGGTTTAA
AGGGGGAAAATGGGGATGGGGGAGAACCGGGATGGGGAAACCGTGAGGGGATTTGGGCGGGAAAATCGTGAGGGGGATTTGGGCGGGAAAACCGTGAGGGGATCTGGGAGA
TTTTTTTGGGGTTTTTTTTTGAAGATTTTTGGGTATTTTATGGGGATTTTTGGGGTGATTTGAGGGGATTTTTTGGTGAAGATTTTGGGGATTTTGAGAGGGATTTTTTT
GGGGATATTGGGAATTTTTGTGGGGATTTTTTTGGGAATTTTGGGGGGAATTTTCAGGATTTTTGTGGGATTTTTGGGCTTTATTTTTTTGGGAAATCTCAGGGGATTTTG
GGGTGATTTTGGGGGATTTTGGTGGGAATTTTTGAGGGGATTTTTTAGGATTTTTGGGGGAGTTTTATGGGGATTTTGGGCTGATTTCTTGGGATTTTGAGGGGGATTTGG
GGGATTTTTGGGGACATTTTTTGGGGTTTTTTTTGGAAGATTTTTTGGGGATTTATGGGGATTTTTGGGGTGATTTGAGGGGATTTTTTGGTGAAGATTTTGGGGATTT
TGAGAGGGATTTTTTTGGGGATATTGGGAATTTTTGTGGGGATTTTTGGGGGAATTTTCAGGATTTTTGTGGGATATTTGGGCGCTTTTTTTGGGAAAATCTCGGGATTTT
GGGGGGGGATTTTTTTGGGGGATTTGGGGGTGATTTTGAGGGGGATTTGGGTGATTTTTGGGGAGATTTTTTTTTGGTTTTTTTTGAAGATTTTTGGGGATTTTATGGGGA
TTTTTGGGGTGATTTGAGGGGATTTTTTGGTGAAGATTTGAGGATTTTTAGGGGGATTTTGAGGGGGATATTGGGAATTTTTGGGGGGATTTTGGGGGAATTTTTTTGGGG
GATTTGGGGGTGATTTTGGTGGGAATTTTTGAGGGGAATTTTTAGGATTTTGGGGGGAGTTTTATGGGGATTTTGGGCTGATTTCTTGGGATTTTGAGGGGGATTAAGTGA
TTTTTGGGGAGATTTTTTGGGGGGTTTTTTGGAAGATTTTTTGGGATTTTGGGGGGGATTTTATGAGGATTTTTTGGTGAAGATTTGGGGATTTTGAGGGGGATTCGGGTG
ATTTTTGGGGGGATTTTTTTGGGGATTTTTGGGGGAATTTTCAGGATTTTTTGTGGGATTTTTGGGCTTTTTTTTGGAAATCTCAGGGGATTTTGGGGGGGATTTTTTGGT
GTATTTGGGGGTGATTTTGAGGGGGGTTTGGGTGATTTTTGGGGAGATTTTTTTGGGTTTTTTTGGAAGATTTTTGGGGATTTTTGGGGTGATTTTATGGGGGTTTTTTGG
TGAAGATTTGGCGATTTTTTGGGGATTTTGAGGGGGATATTGGGAATTTTTGTGGGGATTTTTTTGGGAATTTTGGGGGAATTTTCATGATTTTTGGGGGATTTTTGGGCT
TTTTTTTGGGGAAAATCTCGGGATTTTGGGGGGGATTTTTTGGAGGGATTTGGGGGTGATTTTAGGGGGATTTTGGTGGGAATTTTTGAGGGGACATTTTAGGATTTTTGG
GGGAGTTTTATGGGGATTTTGGGCTGATTTCTTGGGGATTTTGAGGGGGATTTGGGGGATTTTTGGGGAGATTTTTTGGGGTTTTTTGTGAAGATTTTTGGGGATTTTGGG
GTGATTTGAGGAGATTTTTTTGTGAAGATTTGGGGATTTTTAAGGTGATTTTGAGGGGGATATTGGGAATTTTTGGGGGGATATTTGGGGAATTTTTGGGGGAATTTTCA
GGATTTTTTGTGGGAATTTTGGGCTTTTTTTTGGGGGGGGAATTTTGGGATTTTGGGGTGATTTTGGGGTGATTTTGGTGGGAATTTTTGAGGGGACTTTTTAGGATTTTT
```

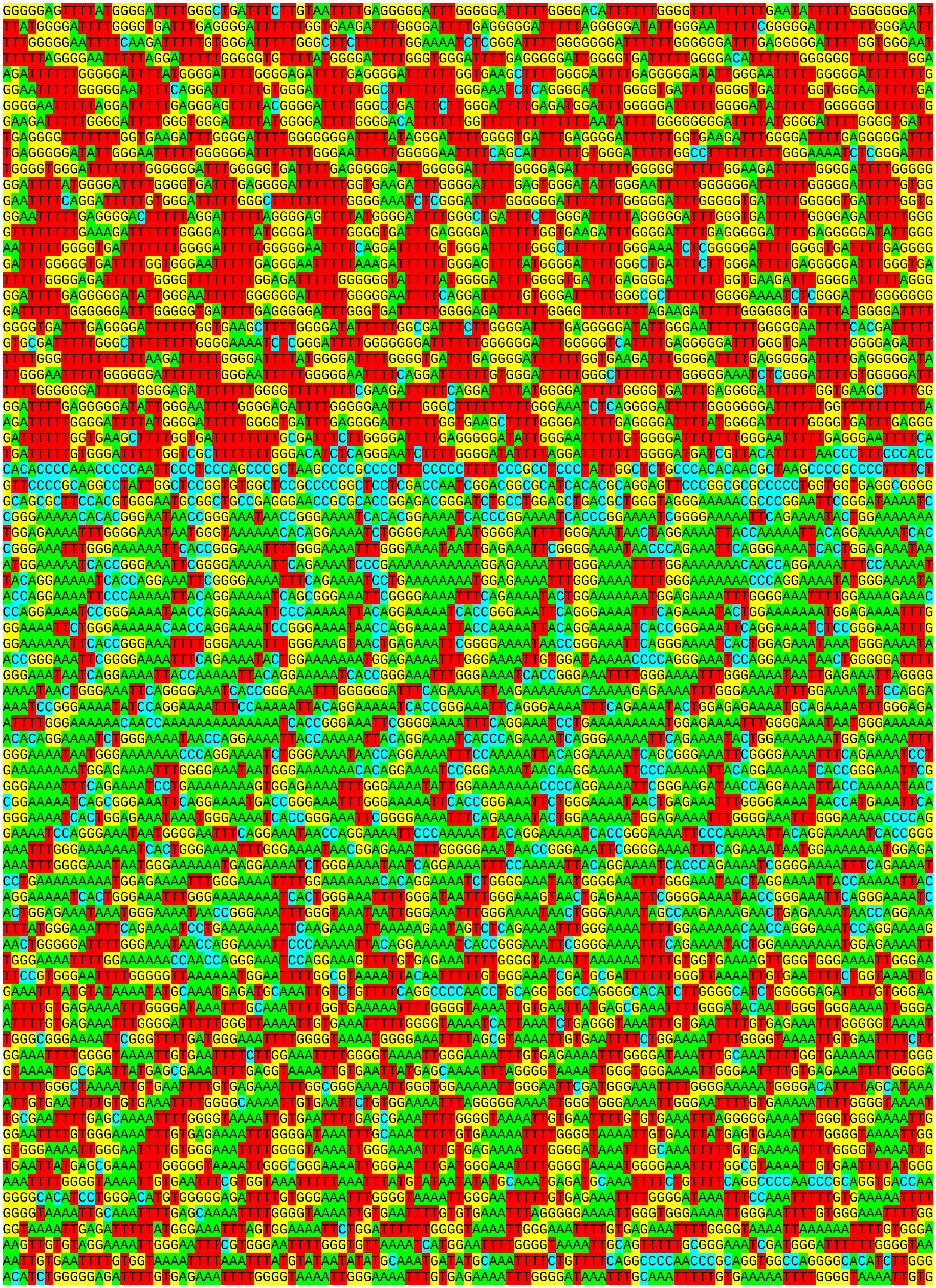

Figure S4, page 3

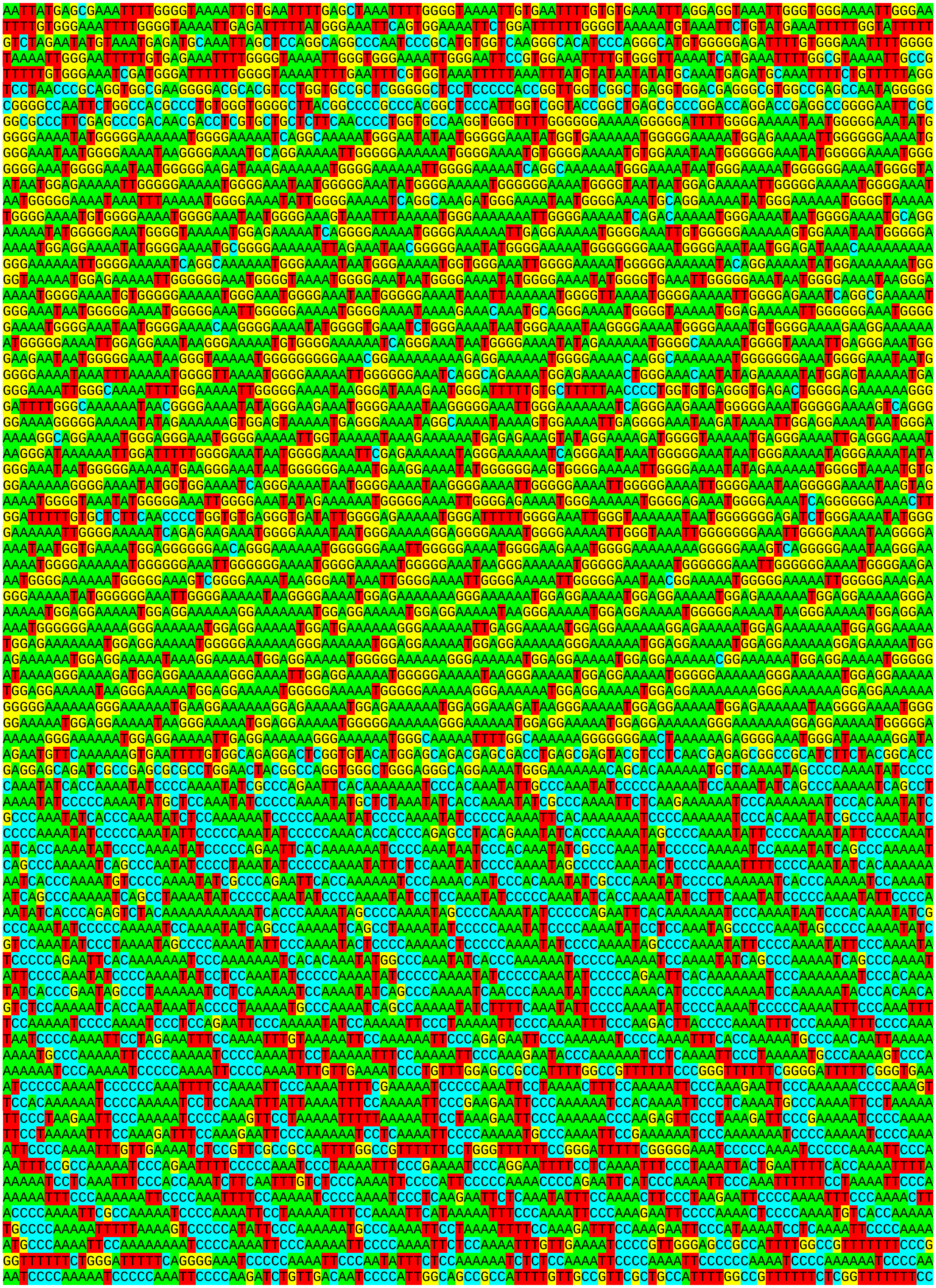

Figure S4, page 4

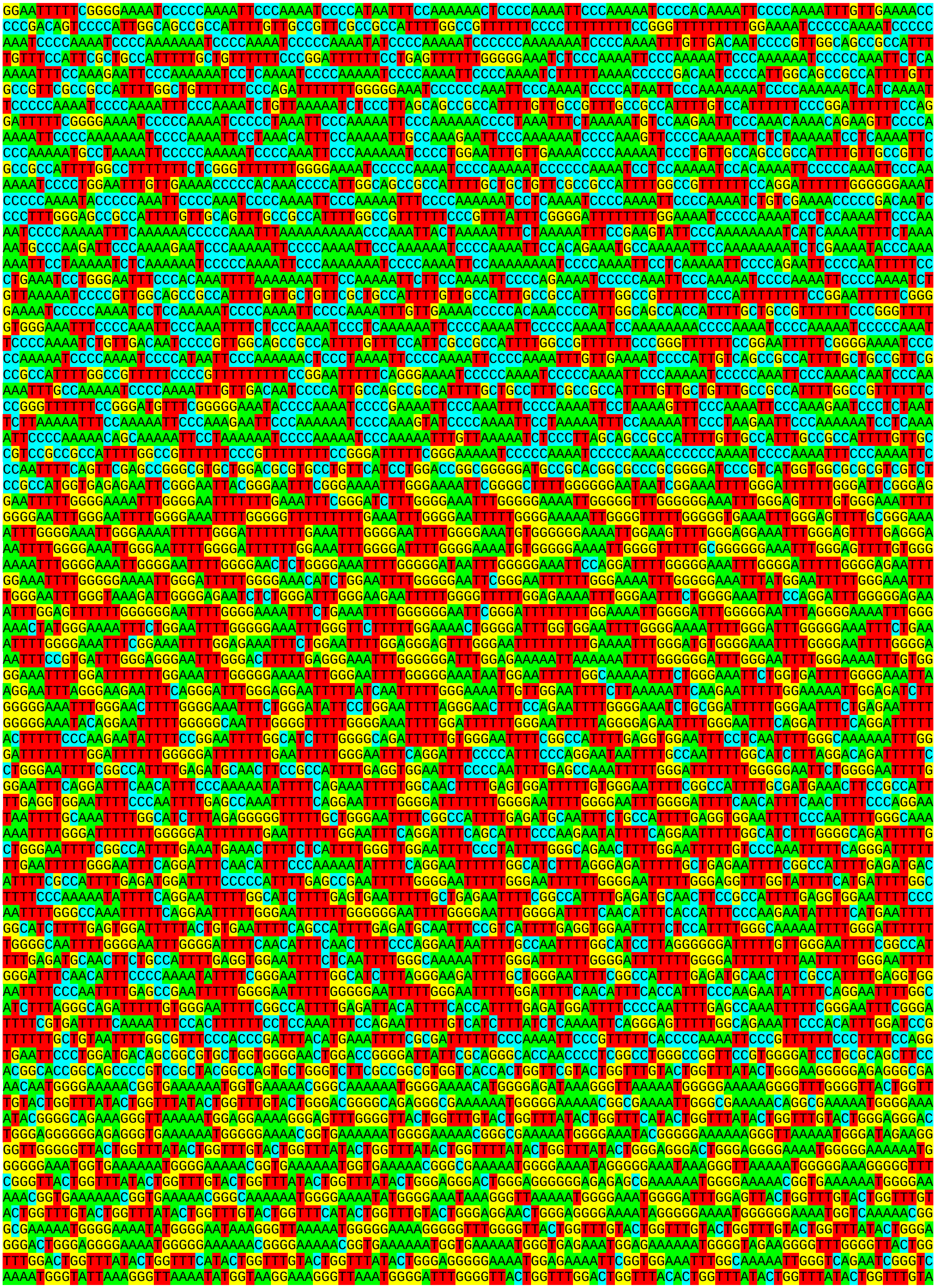

Figure S4, page 5

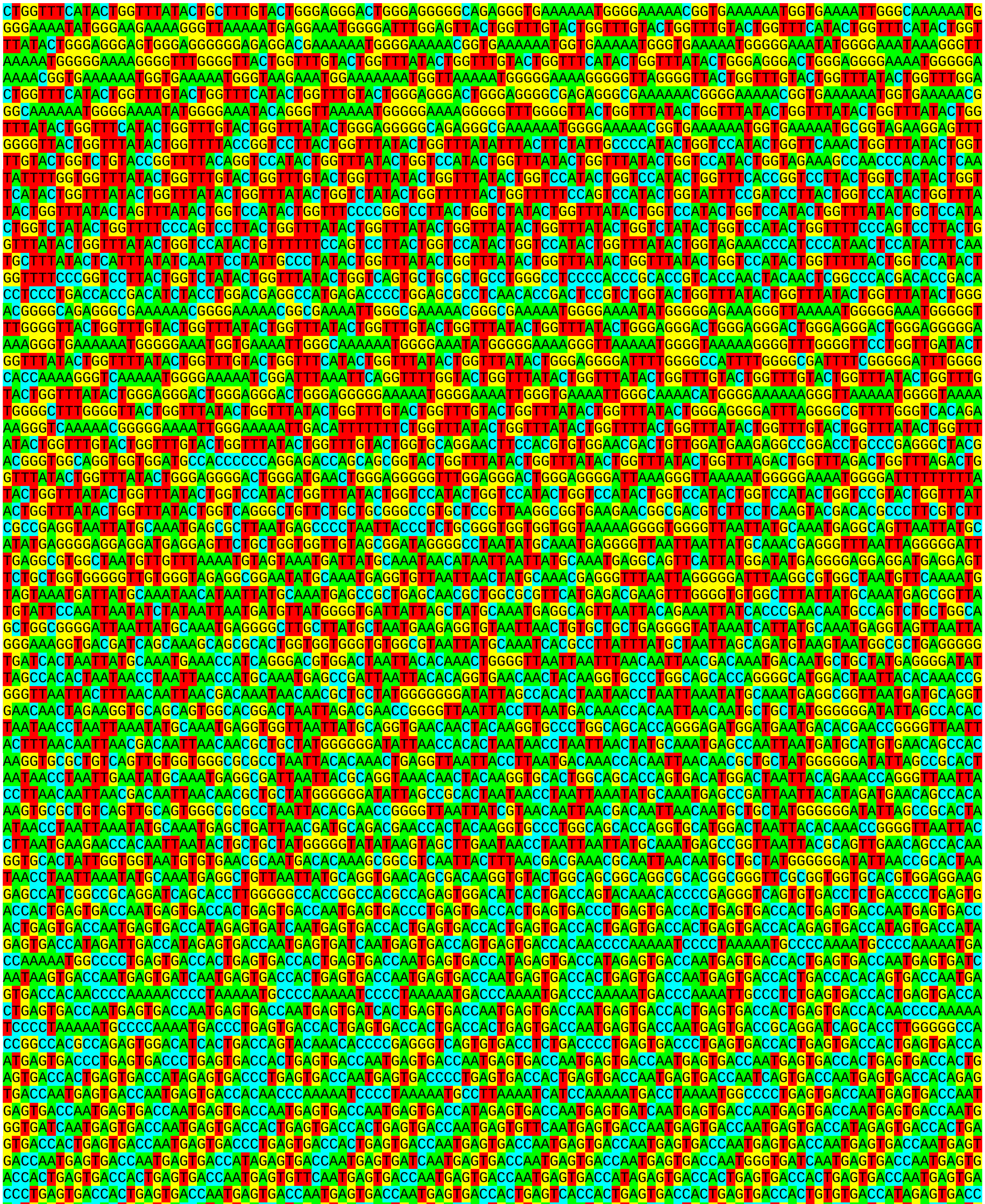

Figure S4, page 6

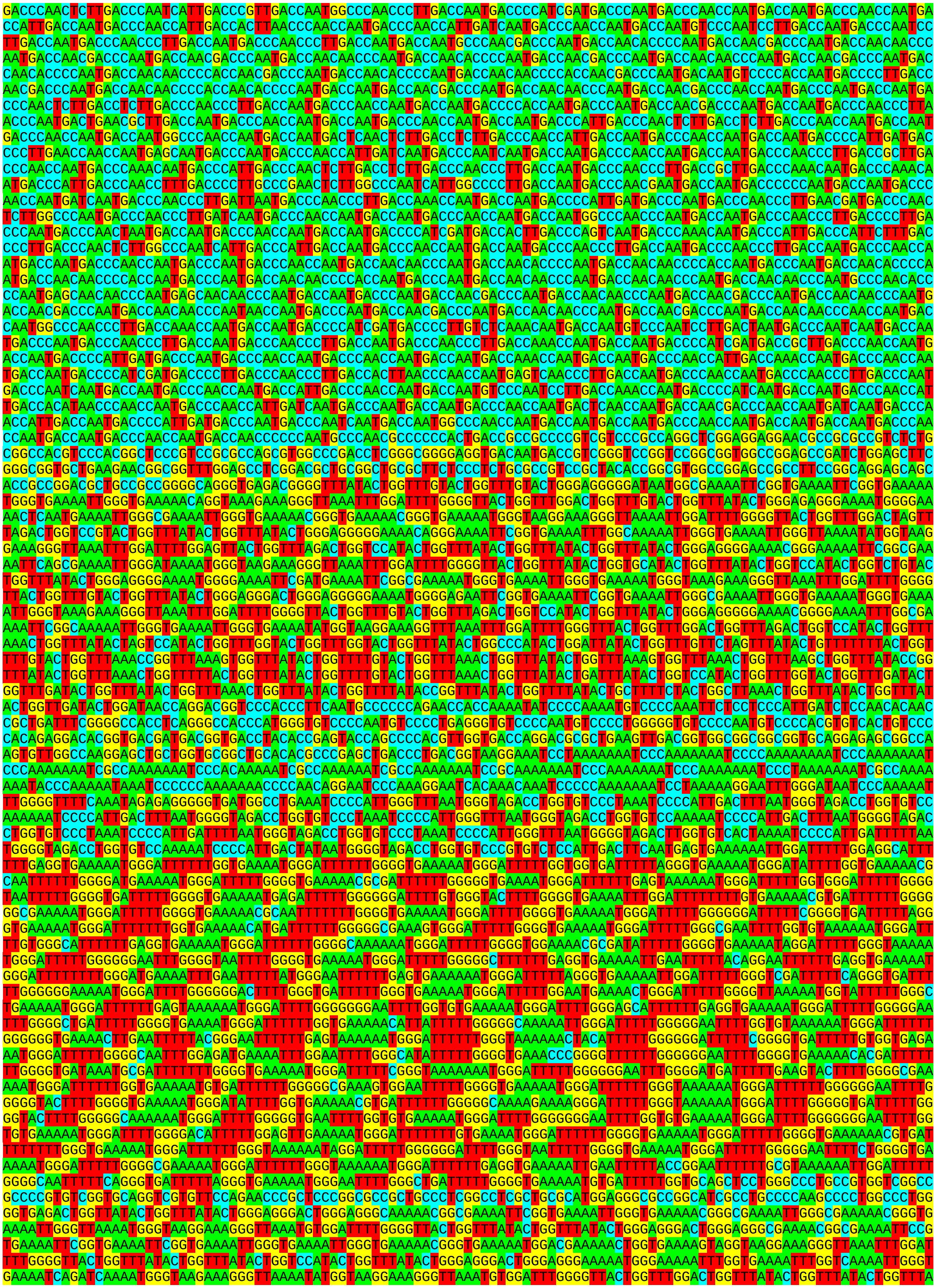

Figure S4, page 7

TACTGGTTTATACTGGTTTATACTGGGAGGGGAAAAACGGAGAAAATTCGGTGAAAATTCGGCAAAAATTGGGTGAAAATTGGGTTAAAAATGGGTAAAGAAAGGGTTAAA
TTTGGATTAGGGGTTACTGGTTTGGACTGGTTTATACTGGTCCATACTGGTCCATACTGGTTTATACTGGGAGAGACTGGGGGGGAAAATGGGGAAAATTTGGTGAAAATT
GGGTGAAAAATGGACAAAAAATGGGCAAAAAATGGGTAAGGAAAGGGTTAAATTTGGATTTTGGGGTTACTGGTTTGGACTGGTTTGGACTGGTTTATACTGGTCCATACT
GGTTTATACTGGTTTATACTGGGAGGGACTGGGAGGGGAAAAATGGGGAAAATTCGGTGAAAATTCGGCAAAAAACGGGCGAAAATTGGGTGAAAAATGGACGAAAAACGG
GTGAAAAATGGGTAAGGAAAGGGTTAAATTTCGATTTTGGGGTTACTGGTTTATACTGGTTTATACTGGTTTATACTGGTTTATACTGGGAGGGACTGGGAGGGGAAAATG
GGGAAAATTTGGTGAAAATTGGGTGAAAATTGGGTGAAAAACGGGTGAAAAATGGGTTAAAAATGGGTAAGGAAAGGGTTAAAAGTGGATTTGAAGTTACTGGTTTATACT
GGTTTGGACTGGTCCATACTGGTTTATACTGGGAGAGACTGGGAGGGGAAATTCTGTGAAAATTGGGTGAAAATTGGGTGAAAATTGGGTGAAAAATGGATGAAAAACGGG
TGAAAAATGGGTAAAGAAAGGGTTAAATTTGGATTTTGGGGTTACTGGTTTGGACTGGTTTATACTGGTTTATACTGGTTTATACTGGGAGGGGAAAAACGGCGAAAATTC
GGTGAAAATTGGGTGAAAAACGGGTGAAAAATGGCTGAAAAATGGACGAAAAAACGGTGAAAAGTAGGTAAGGAAAGGGTTAAATTTGGATTTTGGGGTTACTGGTTTATA
CTGGTCCATACTGGTTTATACTGGGAGAGACTGGGAGGGAAAAATGGGGGAAATTTGGTCAAAATTTGGCAAAAATTGGGTGAAAATCAGATCAAAATGGGTAAGAAAGGG
TTAAAATATGGTAAGGAAAGGGTTAAAAGTGGATTTGAAGTTACTGGTTTGTACTGGTTTATACTGGTCCATACTGGTTTATACTGGTTTATACTGGGAGGGCAAAAACGG
CGAAAATTCGGTGAAAATTGGGTGAAAATTGGGTGAAAATTGGGTGAAAAATGGATGAAAAACGGGTGAAAAATGGGTAAAGAAAGGGTTAAATTTGGATTTTGGGGTTAC
TGGTTTGGACTGGTTTATACTGGTTTATACTGGTTTATACTGGGAGGGGAAAAATGGGGAAAATTCGGTGAAAATTGGGTGAAAATTGGGTGAAAAACGGGTGAAAAATGG
GTAAAGAAAGGGTTAAATTTGGATTTTGTGGTTACTGGTTTATACTGGTTTATACTGGGAGAGACTGGGAGCGAAAAATGGGGAAAATTTGGTCAAAATTTGGCAAAAATT
GGGTGAAAATCAGATCAAAATGGGTAAGAAAGGGTTAAAATATGGTAAGGAAAGGGTTAAAATTGGATTTTTGGGTTACTGGTTTGTACTGGTTTGGACTGGTCCATACTG
GTTTATACTGGTTCGTACTGGTTTATACTGGGAGGGAAAAATGGGGAATATTTGGTGAAAATTTGGTAAAAAATGGATGAAAAACCGGTGAAAAGTAGGTAAGGAAAGGGT
TAAATTTGGATTTTGGGTTACTGGTTCTTACTGGTTTATACTGGTTCATACTGGTCCATACTGGTTTATACTGGTTTATACTGGGAGGGGAAAAATGGGAAAATTGGGCAA
AAATTGGGTCAAAATGGGTAAGAAAGGGTTAAAGTATGGTAATGAAAGGGTTAAATGTGGATTTGGGGTTACTGGTTTATACTGGTTTATACTGGTCCATACTGGTTTATA
CTGGTTTATACTGGTCCATACTGGTTTATACTAGTTTATACTGGGAGGGACTGGGAGAGGGAAAATAGGGGAAAATGGGAGAAAATTGAGTCAAAATTGGCTAAGAAAGGG
TTAAAATATGGTAAGGAAAGGGTTAAATTTGGATTTTGGGGTTACTGGTTTGGACTGGTTTAGACTGGTCCATACTGGTCCATACTGGTTTGTACTGGTTTATACTGGGAA
GGGGGAAATGAGGAAAATTGGTGAAAATTTGGCAAAAATTGGGTGAAAATTGGGTTAAAAATGGGTAAAGAAAGGGTTAAATTTGGATTACTGGTTTGTACTGGTTTATAC
TGCTCCATACTGGTTTATACTGGTTTATACTGGTTTGTACTGGTCCATACTGGTTTATACTGGGAGGGACTGGGAGGGGGAAAAAGGCGAAAATTCGGTGAAAATTCGGTG
AAAATTGGGTGAAAAACGGGTGAAAAATGGGTTAAAAACGGGTTAAAAATGGTTAAAAATGGGTAAGGAAAGGGTTAAAAGTGGATTTTGGGGTTACTGGTTTGTACTGGT
CCATACTGGTTTATACTGGTCCGTACTGGTCCATACTGGTCCGTACTGGTCCATACTGGTTTATACTGGGACGGAAATATGGGAAAAATTCGGTGAAAATTTGGTGGAAAA
CGGGTGAAAAACGGGTAAAGAAAGGGTTAAAAGTGGATTTGAAGTTAGTGGTTTATACTGGTTTATACTGGTTTATACTGGGAGGGGGAAAATGGGGGGAAAATGGGAAAA
AATGGGGAAAATTGGGTGAGAAATGGACAAAAATTGGGGCAAAAATGGGAAAAAAAATGGAAAAAAATGGGGAGAAAATGGTTAAACTGGTTTGTACTGGTTTATACTGGTTT
GGACTGGTTTATACTGGTTTATACTGGGATTTTGTGCGGGGGAGAGCACCAAAATTGGGAAAAAATGGGAAAAATTGGGAAAAAATTGGAAAAAAATGGGAAAAAATTGGGA
AAAAAAGAGGAAAATTTGGGTTAAACTGGTTTATACTGGTTTATACTGGTTTATACTGGTTTATACTGGATTTTGTGTTTGGGAGAGCACCAGAAATGGGAAAAAATGGGG
GAAAATGGGAAAAAATGTGAAAAATTGGGGAAAAAATGGGAAAAATTGGGAAAAAAAAGGGAAAAATTGGGTTAAACTGGTTTATACTGCTTTGTACTGGTTTGTACTGGT
TTATACTGGTTTATACTGGTTTTTACTGGGATTTTGTGCACCAAAAATGGGAAAAAATGGGGGAAAATAGGAAAAAATTGGGAAAAAATGGGGAAAAAATGGGAAAAAAAG
GGGAAAAAAGTGGTTAAACTGGTTCATACTGGTCTATACTGGTTTATACTGGTTTATACTGGGATTTTGTGTGGGGGAGAGTAACTGGGAGCACCAAAAATTGGGCAAAAA
TGGGGAAAAATGGGAAAAAATTGGGAAAAAATTGGGAAAAAATTGAGAAAAAATTGGGAAAAAAAATGGGTTAAACTGGTCCATACTGGTCCATACTGGTCTATACTGGTTTAT
ACTGGTTTATACTGGTTTATACTGGTTTATACTGGGATTTTGAGCGGAGGAGAGCACCAAAAATGGGAAAAAATGGGAAAAAAAATGGGAAAAAAATGTGAAAAAATGGGG
AAAAAATGGGAAAAAGTGGGGAAAAAAGTGGTTAAACTGGTTTATACTGGTTCATACTGGTTTATACTGGTTTAAACTGGGATTTTGTGCGGGGAAGAGCACCAAAAACGG
GGCAAAAATGGGAAAAATTGGGAAAAAATGGGAAAAAAAGGGGAAAAAAGGGGCAAAAGTGGCTTATACTGGTTTATACTGGTTCTTACTGGTTTTTACTGGTTTATACTG
GTTCATACTGGTTTTTACTGGTTTATACTGGTTTATACTGGGATTTTGTGCGGGGGAGAGCACCAAAAATGGGAAAAAATGGGGGAAAATGGGAAAAAATGGGGAAAAATT
GGGAAAAAAGGGGAAAAAAAGGGTTATACTGGTTTATACTGGTCCATACTGGTTCATACTGGTTCATACTGGTTTATACTGGTTTATACTGGTTTATACTGGTTTATACTG
GGATTTTGTGCGGGGGAGAGCACCAAAAATGGGAAAAAATGGGAAAAAATTGGGCAAAAATGGGAAAAAGTGGGGAAAAAATGGGTTATACTGGTTCATACTGGTTTATAC
TGGTTTATACTGGTCTATACTGGTTTATACTGGTTTATACTGGGATTTTGTGCACCAAAAATGGGTCAAAAATGGTGAAAAATGGGAAAAAATGGGAAAAATTGGGGAAAA
AATTGGGGAAAAAAGGGCAAAAATGGGAAAAAAAATGGGCAAAACTGGTCCATACTGGTCCATACTGGTCTATACTGGTTCATACTGGTTTATACTGGTTTATACTGGTTTT
TCCCCCCCACAGCTCCCTGGGGGCGGGGCAGACGCTCCGGCTCAGCCAATCAGTGACGCCGCTCCGGGCCGGGCAGCGCCGCCTCGTGGCCACCTTGGAAAGCGCCGCCCT
CCCCCCCCTGAGCGGCTGCGTCCAGTTCAGCGCCCAAACTGGGAGCACTGGGAGCACTGGGAGCCTTACTGGGGATACTGGGAGGGAGGAGAATCATACTGGGAGCACTGG
GAATGGCGGCAGGCGGCTGCGCAGGAGGAGGGGGAGGCCCGGCGGGAGTACGGGGGGTTAA

D

Dot plot: Zebrafinch *TGM1* vs. zebrafinch *TGM1*

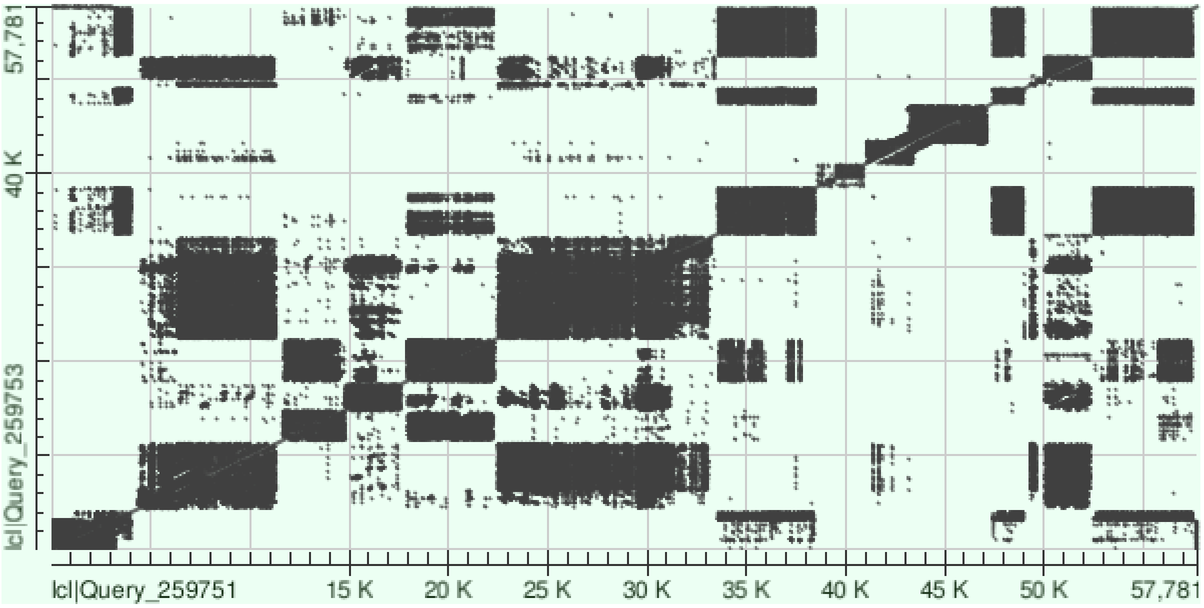

E

Dot plot: Zebrafinch *TGM1* intron 1 vs. zebrafinch *TGM1* intron 1

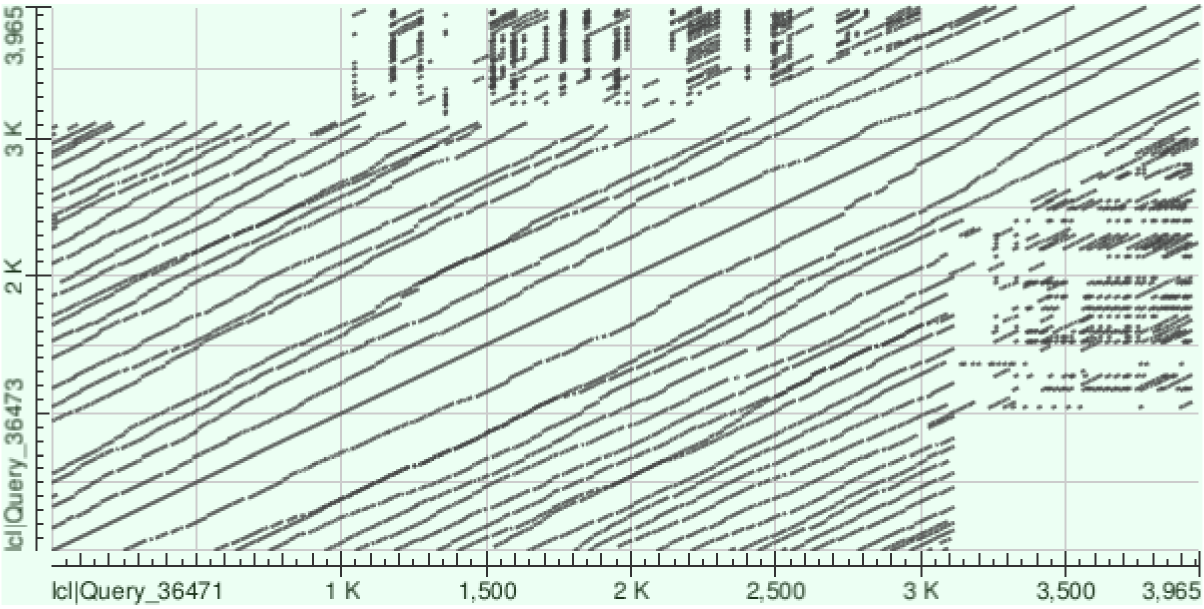

F

Dot plot: Zebrafinch *TGM1* intron 2 vs. zebrafinch *TGM1* intron 2

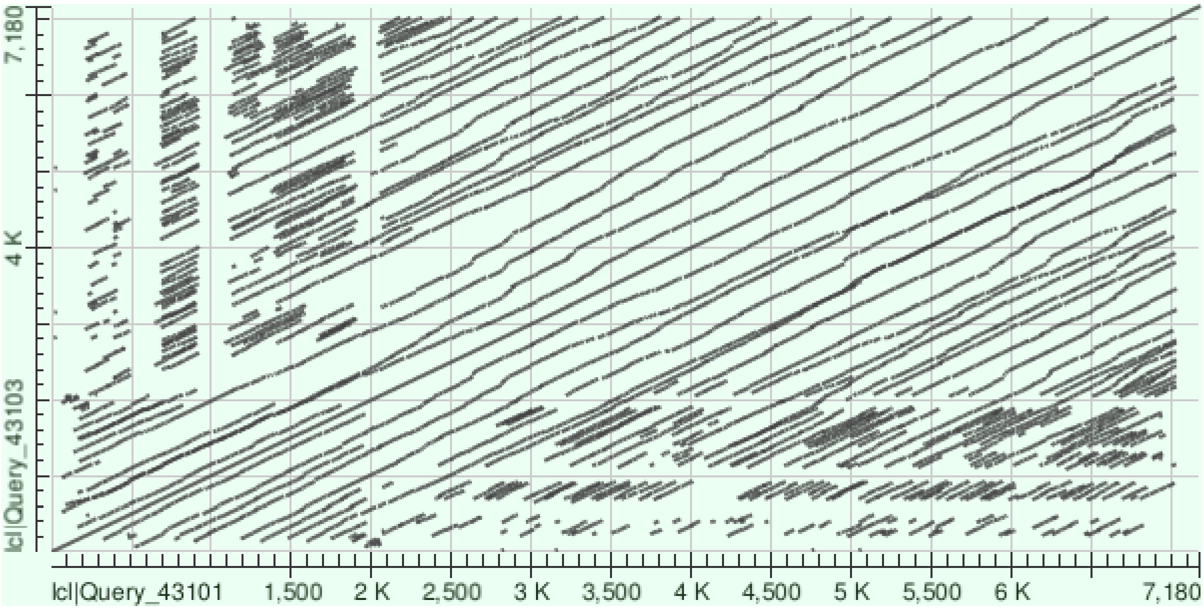

G

Dot plot: Zebrafinch *TGM1* intron 3 vs. zebrafinch *TGM1* intron 3

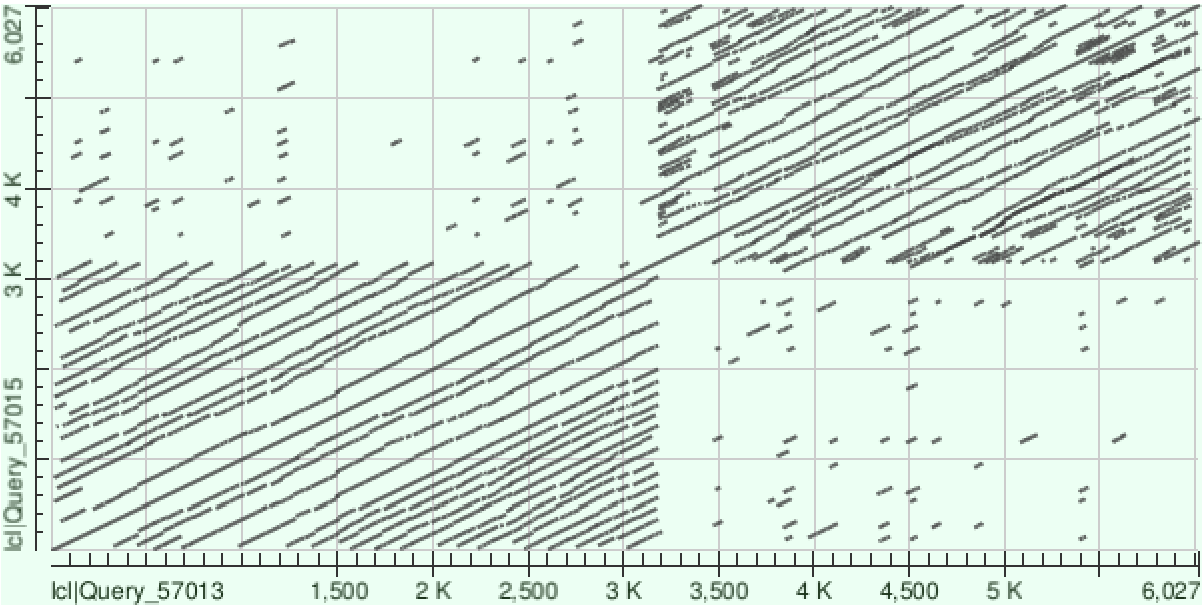

H

Dot plot: Zebrafinch *TGM1* intron 4 vs. zebrafinch *TGM1* intron 4

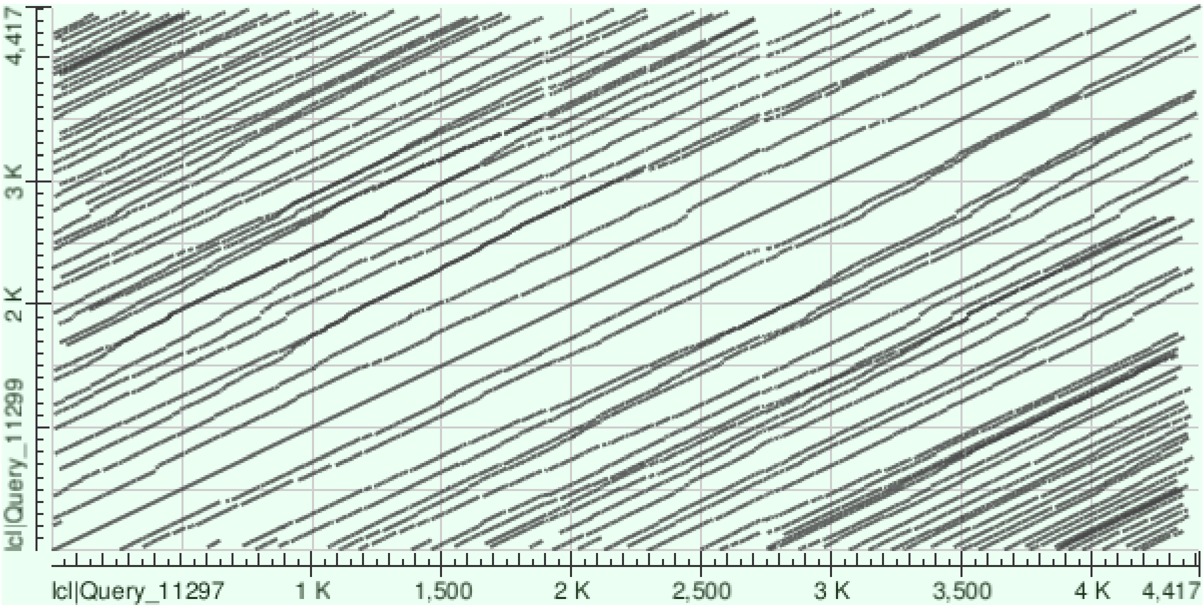

I

Zebrafinch *TGM2* nucleotide sequence

>NC_044232.2:c7145810-7132694 Taeniopygia guttata isolate Blue55 chromosome 20, bTaeGut1.4.pri, whole genome shotgun sequence

```
GGCGGTGGGGGCGGACAGCGGGCGGTGAGGGGCAGCTCCCGCGGCGTCCGGACAGCGACAGGGACCGACATCGGCACTGACAACACCGGCAGAGGAGCGGGGCAGGCACCG
GACGGGCACCGGCGGTACCGGCAGAGGAGCGGGGCAGGCACCGGACGGGCACCGGCGGCACTGGCAGCTCCCGAGCCGCAGCGCCCCGCACCGGCAGCATGGCCGAAGGTA
GGAGGAGCGCGGTGCGAACCCGCAGGTGCCGGGCTCGGTCCCGTCCGCGGCGGGTCCGGTCCGCCGCAACCCGCCCCGGCCGCCGCTCGCCGCCTCCGCCGAAGTCGAGGG
CGGTGGGGAACGGGAAAGACTGAAGCGATCATAAGCCTGGCAGGGTCCGTGCGGGATCCAGCCATTCCCGCCCTCCCGGCTCTCCTCCGGCCCGCAGGATGAAGTTTGCTG
CTATTTAAAGCTTTCCCGGCAGAGCAGGGCGGACAGCCGGGCCGGGAGCGTGTGCCTGGCGGGGTGAGGGGATTTCAGGAGGCTCAGGCAGGAAAAGAGCTTCTGGGCGTG
GGGAATCCTGCATGCTTGGCTCTTCAGGGTAGATAGAGCCCCCGGCTCCCACACAGCTGCCCCCAGAGTGCCAGGAAATGGGTCTGTGTGGGTCCCACGGCACACAGCCC
TCTGGGTGAGACGGCATCACTCTGCTCTGCTGGGCCACAGTGCCGCAGGCTGTCCTGGCTCCCAGCACTGCTGTTGCAATACCAACGCAGGTCCCTCGAGTCAGACACTCA
CACAGACAGATAGCTGGGCAGCTACAGAATCACAGAATTAACTAGGTTGGAAAAGAGCTTTGAGAGGCTCGAGTCCAACCTATGACCTAATACCACCTTATCAACAAGACC
CTGGCACTAAATGCCACATCCAGTCTTTTTTTAAATACCTCCAGGGACAGAAGCTCCACCATCTCCCCGGGCAGCACATTTTAAAGCCCAATCACTCTTTCTGTGAAAAAT
TTTTTCCTAATGTCCAGCCTAAATTTCCCCTGGCGTAGCTTAACACTGTGTCCTCCTGAACACACTGGGCAGCTCCTGCTCCTGAGTGCCCAGGGTGCTGCTACCACTTGC
AGCACCGCAGCCCCCTTCCCACCCATCTCTTGGCACTCATTGGTTTGGATAGATGCACCATCTATCAAATAGATGCACATCTACCAAAGCATATGTGCTTTGAGAAATCTG
CTTTTTGCTCAATCTGCTGAGGATGAAAACGCCAGGGTGACTGGGACAAAGAGGAGCCCTGATGCTGTTCTCTGAACACACTTACTGTTTGGTTTTTTTATTGCCAGCAAG
TTTAACCATGATATTCCACTTGTACAGTCCGTGTTCTGTACGGGGCTGTGCTGCTCACAGAGGGGGAGGCTGGGAGGGAAAGGGGGTGTGTGTGCTCTGTCCCCACTTCAC
AGTTCCCATGGACTGGCTGTGGGGACCCTGCCCTTAGTTACGGACTGTTCAAGCGGCTCTCACATTGTGGTGGGGACAGTGACAAGCCCACTGCCCTCCACTGGGCCCATC
CTGCAGAGTCACCTTGCCCAGAGAGGCAGGAGCAGTGTTTTCTTTTAGCTCTGGGAATGGGGAGCTCGTGGGCTGCTGTTTCCATTTGGCACTGGTCTGAGGAGGGCTTGG
TGGCTACCAGAGCTGGCACAAGGAAGGGCATGGCCGAAGAGAGCCATGGCAGGGGGCAGCAAGCCCCAGCTTGCCCCCTGATTGCCCGCTCATCTGCCCAGTCCCTCGCTG
GCACTGAGCTTGCTGTGCTCCCTGCACAGAATGCTTTTGCTTTTTCTGGTGATTAATACGTCACTGCAGAGCAGTCAGGGGTCAGTGGCCTCAAGTGATGATGAACGATAT
CGCTCCAGCATAGTGCCCAAGGTGGCAGGGACGTGGCTGCTGGTGTTCTGACACACAGCCCTGCCTGGGCTGCGAGCAGAGCAGTCTGCATGAGTCTGGGGCATCAGACAC
CCTCTGTCACCAAATCTACACTTGCTCTCCCCAGTTTGTCAGCAGGTGCAGCACAGTAAAGCTGCTGGAGGCCTGGCTGAGGCACAGGAACATCCTGGGCATTCCTGGGCT
CACTCAGCAGCATGTGCTTGGGATGGGGGCACACAGACAGCAGCCCCAGTGAAAGGGCCCCTGAAAGCAGAGCTGAGCCCTGGGCTTTGCCCCAGTGTTGGGTGCTGTGAG
ACACAGGCCTGGCACAGCTCTGGCAGCACAGTGGTACCGCAGAGGTGCTCCATGGCACAGCACAGCATAGAGCTGGATCAGGAGGGCAAAACGTGAGCTTGGCTGCCAGAG
CTGTGCCCCTCTTTCAGCACACAACGGGAGCCCAGCCCTCAGAGCACTCCTGGAAGGATGCTCAGAGTCTGGCACGGTCACAGCGCCCTCCTGGAGAGATGCCCCGGCACA
TCTGGGAGCACAGGCTGGGTGGGTGGGAGGTTCAGACCCCTGGCCAAGGTCCCAGCCCAGGGCTCAGGGTGGGGACTGCGTGACACGTCCCAGGCTCGGAGCTGTACCTGC
CTCTGCCTTTGCAGATCTGGTGCTGGAGACGTGGGACCTGCACTGCGACCGCAACGGGCGGGAGCACCGCACGGCGGCCATGGGCTGCGGGCAGCTGGTGGTGCGGCGGGG
ACAGCCCTTCGCCATCACCCTGCGCTTCTCGGGCCGTGCCTACGACGAGGCCGTGGACAAACTCTCCTTCAACGTGGAGACCGGTGAGTGCCGCCACATCTGTGTCGCGAA
TGAGCGCTGTCACCACCCCCTGCCCCAAAGCTCGCCTGTGCTCCCACGGGGGCTGTGTCACGGCACCTGCCCCATGTCACACAGTCCCTGTTCCTCACCTGGCAGAGGCTG
GTGCTGTGCACAGCTTTTATATCACAAGAAAAAAAGGTAGAAACTCATCTCCTTAATGAGACTGGAAGAGAAGTGATAAAGAAATACAACAAAAAGCCACTGGCGTCATCA
CCTCCTCCTCCTCCTCCTCCTCCTCCTCCTCCTCCTCCTCCTCCTCCTCCTCCTCCTCCTAGGGCCAAGCAAGACTAAGGGAGCATGACAGAGCTGCCTGCTCACCTTGGC
TGAGGCCAGGATCCTGCTCAACCCTCCTGCCCCTCTGAGAAACATCAGCACAGTGTTGTTTCAGCCAGAGCTATCACAGCATTTCTGCTGATGGGCTTTACACCCAGCCCT
GCTGCCTCTCAGGCACCCAGGACGGGGTTTGTTCAGGAAACACAAACACTGGAGAAAGGAAATGTTCCCACGGAGCCCCCCAGTGCAGCCCCCGGGCCGGGGCTGGGCTGG
GCTGTTGCTCTCACAGCCAGGGCTGGGTGCAGCCACCGGGCAGGGGCTCAGCCCTGCCAGCTCAGAGCAAAGGGAGAGGAAACTATTTCTCAGGCAGGAGTTATTTTGGGG
CTGAAGCACCATGCTGAGTCAACCCAGCCAGAATTCAAAGTAAAACTGAGCCCAAGTTTCCTCACATAGTAAAACTCTCCTTTTTCCATCCCAAAAGCTCCGGTGACAAAA
CAAAACCCAGCACCTCCCAGCTTCACCCCTCACCAGAAGCTCCCTCGGTGCCCTAGCAGCCCCATGGGTTTCTCCAGACCATGCTACAGCCAGGCAGCCCCGGGCTGGTTT
TGCAGGGGAGTAGCCACAGGCACAGCACAGGCACAGCCTCCCAGGCCAAGGCACAGGCTGGCAGAGGCTCCTCTGGGCTGGAGCCCAGCCCCAGCGCTGCAGAAAGAGGGG
AAAGGTGCTTTGGTGCTCGCTGAGGCAGCAGCCCCAGCACGTTCCTCACTGGGGCCAGACCTGTCAAGGCAAGGGCCGCCAGAGCTGTCCCTCCATCCTGGGACAGCAAAG
CTACCGAGCTCATGCGCTGCCTCCCACAAGAAAGGGGGAAGGAAGAGTAGAGTGAAAACAGAGATGTATTTTTGCACTAACCATGTTGGCAGAGCCCCGTTCAGGAGTCCA
GGGGGATGTTGTGGCAGCAAGAGGAGGAGGAGGAGGAAGAGGAGGAGGCAGGCTCTGGGAGTCTGCCAGCTCACGGCAGCGCCGGGCTGGCTCTTGCCCAAAGGACAC
CGTGAACCCAGCCCAGCCTCCCCGAGAGGAGAAACTGTGTCACTACCATGAGTTCACGAGTATTTTTGGTGACTGGCCTGTGGGCGAGGGCTGGCAGGGGACCAGTCGAGG
CTGTGCTCCGGCAGGGAGAGGGGACAGCAGTGACACCGCTGCCTGGGCTGTCTGCCAGGCTCTGCATGGCCAGGGAGCCGCAGGGCGTCACCGCTCCGGGAGCGGCTCTGG
AGCTGGCTCCGTGCAGGTGGCTTGGCCATGGCTGCAGCTCGGAGGGGCCAGCGAGGATCACACTGTCCCAGGCTCGGCGCTTTGGCCACTGAGGTCATCCCTCAGCCCGGG
ATGTTTGCAGCAACGCCTGAGGCTGCAGACACGTACTGGGGCACCCAGTGCCAGGGGTCTGTGGCTCATGGCCGGGCCCTGCCAGCCCCCATGCCGTGGCTGCAGGGACCC
AACAGTGCTGCTCGGAGGAGAGGGAGCTGGTGCATCTGAGAACAGAGACCTGGCAAGCACCCCTGTGCTCTGCTGGCCCCCCCAGGACGAGTATGTCCCTTCCCTGCGCCC
TCAGTACCTGACAAACTACACAGAGAATGTTCCCAGACTGTGACAGCCCAGATCCAGGCAGCTGCTGCAGGGCAGGGGGGTTTGGAGCAGCTGCCTTTGGTGCACCCTCCA
TGGCAGGGGGCTCCCAGGGGTTCTGGAGCACTGGAGAACTGACCTACTCCTTCTTGACAGGACCCTTCCCCAGTGAGACATCAGGAACCAGCTCCCACTTTGTCATGACTG
ACTGCCCAGAGGAGTCGTGCTGGAGTGCTGTGATCCAGCAGCAGGAGGGGGAGTCCCTTTCTGTGTCACTCTGCTCCCCAGCCAACGCTTGCATCGGCCGCTACAGGCTGA
CCCTGGAGACCTGCACCGACTACCAGGGCTCCAGCTACCAAATCGGGGACTTCATCCTGCTCTTCAACCCCTGGCACCCAGGTGAGGCTTTCACCTCTCTGACTCCAGGGA
TTCCAAGGCTCAGGCAAACACAGGATGCTCCCCAGGCATCTCCCCCACAGGTCTCATCCCAGTGCAGATCCCAGGCATCCCAAACCCTGGTCTGAATGCAGGTTTAAGCAG
AAAGCTTCACTCTGCTTCCATCTCCACACAATCCCTGCAGGAGGTTGGGGTCAGGAGCACCGTCCCCACCCTACAACACACACAGCCCCTTTGCCACCACCCCAAACACCC
AACGGCCTGTCCAGGCTGCCCCCACCACACAGTGGGAGATCACCACTGTGCTGCTGGTGTGAAGCCCTGGGCAGTCCCTGGCACCAGCTCCTGGCCAGGCAGGGGACAGGG
GAAAAGCCTCTAAATGTCCCTAATTGCCCCCAGTTTTCCTCACTTGCCCCCAGTTGTCCCTAATTGCCTCCACCAGGCCCATTCTCACCCATCCTCACCCAGTCACCCAGC
TGTGGACTCAGTGACATGGATATCACTGTCCTGGTGAGATTTGGGCAGCCAGACCCAGAGGAGAGATTCGATTCAGTGCTGTAAGGTTCAACAGCCCTGGGATTTTCCTCT
CTCCATGTCCTTCCCTTGGACTGGCTGATTTCCTGGATCTCCAACGTCCCACGCTGGATCTGCAGAGATAAGGGGCCATAGGAAGCTCTGCTCAGCCAGGGCTGTGCTGAG
CACTGGGGGTGGATGTGTGGGGTTTCCTTCTGCAAACATTTCCAGTAAGTGTTGATTTCCACAGCAGTAGCTGCTGTGAGTGAAATGGCTTTGCCTGTGCTGAGCCAGCAC
ACCCACTCCCACTCCTCACTGCACCCCCAGGGAGGTCTCCCCTGCTGAGAAGAGATCCATCTCCATTAAATAAAAGGGAGGAAAGACAGAGACATGAGAAAGAATGAAAAG
CAAACCACAAATCCGCTGAAATTTTCCCATCACTTTAAATCAACCTTGGTTGAAATTCCCCAGCAGCTGTTTAAGGCATGAGCAGTTCCAGCCCCTGGTGCCTCATTTCCC
ACTGGATCCATCCACAGGGAGGGGTCAGGAAGCTGGTTGTGGAGGGCAGAGCCCTGTCCCCATCACTTGCTGTCCCCACAGAGGACACAGTTTTCATGCGTGACGAGAACG
AGCGACGTGAGTACGTCCTGTCCCAGGATGGGCTTATCTACCAGGGCACCTGTGATTACATCTACTCCATCCCCTGGAACTTCGGCCAGGTGAGCGGGCAAGGCCCCCTGG
GACCCTTGGGACCCCTGGCTGGCCCAGGCCATGCAGCTCCCAGATGCAGGGACTGGTGCCATGCATGTGTCCCTCACAAACGTACACCTCAAATGCTGTCAGCACCTTGGA
GTCCAGCCAAGGGTCAAAGAAACCGGAATCTCATTTCCAAGAGGGAATGCGCTGACTTCAAAGCATGGCAAGGCAAGGGGAGTGTTTCCCCAGGGCTCCTGGGGGCACCAT
GTGTCCCTAATCTGGGTGGCCCTGCTGTGCTGGGAGCTGGTGCCTGATAATTCCTGTGCCAGGGGTGCTGATAAGGCAGTGCTCACACCCTGACCTGCACAGAGCACAGGG
CTTGTTCAGCCACCCTCCCTGAGCAAAGGGCTGGGGCCCCACAGCAGTGGGGGTGCCCATGGCCTCCACAGGATCCGTGCCTCAATTCAAGGAAGGGCCGTGACTCAGCCT
CGATGAGAAATAGATTTTCCTGGAGGTCAAGTCCAAGCCCTGACCCCTGTGAGGTTGCAGGAAACCAGATCCAGCCCCAGTACGTAGGCAGGAGGTCACCCCCAGGCTTCA
CAGGCTGGCTGGGGACAGTGGGAACACCACTCAGCGGATGCTGGCCGGGGAGGGACCCTGAGGAGGTGTGACCCCTGTGCCCAGCAGTTCTGCTGGCAGGGGCATCACAGT
GAGCCCGAGGGGGAGGACAGAGGTGGCAGAGCCCCTTTGGGGACCTCACAGAACCTTTTTCCCCTGCTCTCCACCAGTTTGAGGATGAGGTCTTAGCCATCTGCCTCAACA
TGCTGGACATAAACCCCAAGCACCTGCGGGATCAGAACCAGGACTGCTCGCGCCGCAATGACCCCGTGTACATCGGCAGGGTGGTGAGCGCCATGGTGAGTCCGGGCTGCA
GCCAGCAGTGTCCCCAGCACCCCACATTCCCTGGGCTGCAAACACCCCCAGTACAGCTGTGGTAAACAGCATTTATCATTCCCTGGGCTGCAAACACCCCCAGTACAGCTG
TGGTAAACAGCATTTGCCATCCCCCCCCCCAGTTACACCATTTTCCTTCTCTGCGGTGTTTCCTCTCTCTTTTACATCCTTTCTCTGTTACTGTTCCTATATGTATAAGAG
GTTTTTTAGAGGAAACAAAAAGCAGACATGGGCACAGATCATTCCAGGGATAATCACTCCCAAATTCCCAGGCAGGAGGATATGTTTCTAAAATTCATCCCTATTAGCACT
GTGTCCACCTGCATCCCATTGAGCTTTTGCATGGATACAGGATATTAAAGTGGGTGACAGTGCGTGTGAATGGGGCAAAGGCTCTTTCCCTCCCTCCCTCCCTCCCCAGCT
TGTTCCTGCCCCTCCATCCTTCCCCTTTGCCCCTGCCACAGGTGAACTGCAATGACGAGGACCGAGGAGTGCTGTTTGGGCGGTGGGACAACTGCTACGACGATGGGATGA
GCCCCATGGCTTGGATTGGGAGTGTGGATATTCTCAAGAGGTGGAAGAAGTTTGGATGCCAGCCAGTCAAGTACGGCCAGTGCTGGGTCTTTGCTGCTGTGGCGTGCACTG
GTGAGAGCAGATTCCTCTGGTCTCCCCTTCTCCACAGGACAGGGTTGTCCCATCTGCCCCCATCTCCCACCCCAGACCCTGCCCACATCCACCCCTGGTGCTGCATGCAGA
GGGGAGAGCTGCAATCAGCCTGCAATGATTCCTTTGCAATTAGCTCTCCATTACTCTAGTTTGTCAGGAAAAGAGGGAAAAAAATGGATAGAGGGAAAATAGAGGGAAAAA
```

ATGATACAAAAGGGCTCTGTTTTTTCAATCAGCGCTGCCTCCCAGGCCAGGCAGCAAGGGAGGAACTGAGGCAGGCCAGGGTGAGGGAAGTGGCTTTAAGGCCAAGTTTGC
AATAAAGTCACTAATCCACTGAGATCAAAATAATGTCGTGGCCTCCGGGGCAATCAGACTGGGGAAAAAGAAGACAACAATTCCAGAATTGCTGCTGCTGGGAAACACTCA
CATATGCTCGTGCAGCTTTGCCCCAAGAAAAACGCTGCCTGTGAGAGCGTTGATGTGTCACACACAGACGAGACTCTTTTTAGAGGAGAGAAATAGCAGAAAACTAAAGTG
GATTTTCAGCGACTCACAGAGGTTTATAGCCTTCCTTTGAAGCACTGTAACTGCCTCCCTGCTCCCTGGGGAGCTCCCTGTGCAGGGAAAGACAGCTGGGAGATGAAATCC
GGGGTTCAGCACGGCTTCCCACACTCTACCTCCAAAGCTCTCACAGACACTTTTTGTGCCTTTTTTTGCTGCAATATCAAACCTGTCGGGCTGTCAGAGCCAGGTGCCACC
CAGCCAGCTTGCCTGTGTGCTGTGGCTGTGGGAACTGCTTCTCCTGGGGCTCAGTCCCCAGGCAGCCCTTTCACAGGCAAAGGGAAGGGCAGGCAGGAGTTGTTCAAGCCT
GGGGCTCCATGGGACACGAGCTGTGCAAACCTGGGGCTCTGTGGGGTGGGGGTGGTGCAAACCTGGGCCCTGTCTCACCCTGTCCTGCTCCCTGCAGTCATGAGGTGCCTG
GGAATCCCCAGCCGGGTGGTGACCAACTACAACTCAGCCCACGACACAAATGCCAACCTGACCATTGACCGCTACGTCAACGAGAATGGGGAGCAGGAGAGGCAGTCCTGG
GACAAGATCTGGTGAGCAGCACAGCAGGACCTCAGCCTGCCCACCCCTGGGCTGCTTCTGCCCCTTCCCCAGAGCCTGGCAGGTGCTGGGGGAGGCTGAGGCTCCTACATG
CTGGTTTCCCTCACAGGAACTTCCACTGCTGGCTGGAGTCGTGGATGGCCCGTCCGGACCTGGCAGCTGGCTACGATGGGTGGCAGGTGCTGGACCCGACGCCTCAGGAGA
AGAGTGAAGGTACAGCGTGCTCCACACGGCAGCTCCATCCCCCTCACACCCTGACATGGCTGGACCGCCCCACCACAGCCAGCCCTTGTGGCTGAGCCCTGGAGGGGTGAC
AGTGGCTCTACTCCGTGCAGGAATTTACTGCTGTGGGCCTGCCCCCGTCAAAGCCATCAAAGAGGGTGACATGCTGCTCAAGTACGACATCCCCTTCATCTTCGCGGAGGT
GAACGCTGACGTGGTGTACTGGGTGGTGCAGGGCGACGGGATGCAGAAGCAGAGCATCCGATCCTCAGACGTGGGGAAGAACATCAGCACCAAGAGCATTGGCAGGGACAG
CCGGGAGGACATCACCCACAACTACAAGTACCCAGAGGGTATGGGGGATTTCAGGGGCAGAGACATCCAGGGAGGGATGTTGCTGCTGGGAAACAGCTGCAGCAGGAGGGC
TGCAGGGCTCAGGGCAGAACAGCCCAGGCCAGGCCCTCCCGTACTCCAGGGACTCACAGGAATTTTGTGCACTGCAGGGTCTGAGGAGGAGCGGGCAGTGTTTGAGAAGGC
AGAGCATCAGAAGAAGTCCATCGGGGAGGAGGATGAGGGGCTGCACCTCAGGATCAAGGTCTCAGAGGGCGCAAACAAGGGCAGCGACTTCGACGTCTTTGCTGTCGTCAC
CAACAACACAGACGAGGAGCGCGTCTGCAGGGTGACGCTGTGCGCCCGCGCCGCCAGCCACAACGGCACCCTCGGGCCCCAGTGCGGCCTCACGGACCAGGTGGACATGGA
CATTGAGCCCAGGGCAGGTGAGAGACCCACTGGTGGGGAAGGAGATGCTTCACCGGGGCTGGGGTCAGGTCAGCCTGAGTTCAGCTGCTCAGTAGGCAATGCCACTCTGGG
GCATCCTCCTCAGTCAACTACCAGTGTGGATTTTTAATCTTTGTGAGAGGGTGAGGAAATTGCTCAGGCGGAGGCTGGAAATGCATTTGGAGCTGGACAAACCGCTGCTGC
TGTAGCTGTTATGGCAAGGAAGCAGGCTGGGCACACCCAGGCTGTCATCCCTAGGCACTCTGTGGCACTTGTCTGCGTCCCTCCTGCTTTCCCTCAGACTTTGGGCTAGGC
TGGGCTCTCTGGTGGGATCTTCCCACACCAGGGAGAGGGGAACTGGCCCAGCTCAGCCCTGAGTGCGTGGTCCCAGGCTGGAGGAAGTGAGAAGCGCTGGGTTTCCTGGCC
AGCAGCGCTGCAGCCTCTGTGTCTCTGTGGGTGGGCGTTCGGCTGTAGCGCTGTCCTGGCACGGCCCCGAACCTCCCTCGCCCTCATCACACGTGAACATCACCATCTGGT
CATTAAACACGAGTTTGGGCTTACGTGAGTTTGCAGAGACCCTTCAGGGCTTGGAAAGGCCACACATCTGCTGTCCTGGAACAGCCAAATGTTTACCCTGAGCCGAGCCCT
GTTCCTCACCAGCCCTCCTGCAAACGCAGCGCTCGGCGCTCTGCCAGCTGTTGAGCTGCTTTGCAGGAATGAGGAATGTGCTGGCTCTTGCCACCATGGAGGGGAGAGGGA
TGTGCCTCTGCTCATGCTGGCAGAAGCACCAGCTGGTACTTGAAGCCTTTTGCAGGGCTGGATGAGAGAGTGGGGATATGTGCTGTGGCCTTTAGGGTACTTAACATTCCT
GTGCAAAAGTTAACCCAACACCATTTTTTCTGAAACATTAATTATGAGCTCCTGGAACTTCTACCCTCAGCTCCTGCCTCAGAATATAGATCCCAGGACTTCCCAGACTGC
ATTCTTTAGGGCACCCCAGTACTTTGGGAAGCCAGGCTTGTTCCTCTCCCCACAGTCTCAGCTGATGCTATGGCACTTTAAACCTTGCCTCATGAATGTCAGGCTCCATTT
CTCATGGTCACAGCCACTCTGGGCTCCTTGTTCCAGGCAGTTCTGCAGCTGGTTGCTTTCAGCCTCCTTCCCTGTCCTTCCCCCAGCAGCCCGATCGCTGAAGGACTTTCA
GATGAACAAGGCTGAGGGCTGAGTGCGGGCAGCTGCCAGGGACCAGGCTGCCTCTGGACCCCTGGTGCAGGCATCACCCTGGAGTCTCGCCCTAGGACATGGCTCCTTCTC
ATCCCCACACCCTGAACATCCCTCCTGGCACTCAGTATTGGAGCCCCAGCCCTGCAGGAACCACAGCTCACCCCCATCCTTGCAAGGCACCTGGAGAAGCTCCTGGGGAGC
CCAGACCGTGCCAGCAGCAGCTGGGTGCCAGTGCAGACTGTGGGTTCTGGGCTGGAGATGCCATGGGTGCTGACCCCTCTCTGCTGCTCTTCCAGAGAAGAGGGTGCCCCT
GCGCATCCTGTACGAAGAGTACAGGGACAAACTGACCCAGGACAACCTCATCAAGGTGGTCGCTCTCCTGAAGGATCACCAGACTGGCGACATCATCGTAGCTGTCAGGGA
CATTTATGTTGAGAATCCACCGATCAAGATCAGGGTAAGGGTCTAATGTCTCATCACAGGCAGGTTTGAGGGGAATGAGTTGCTGTAATCATCTTGGGGTTACCCCTGGGG
CTTGGGCTACAGGTCCTGCTTCACTGAGAAGGGCTGCATCCCCTGGGATGCATCCCTTGGGTCCTGCCTCAGCCAGGGGGCAGGGGTAGCACCATGGCTCCCAGGAGTTGT
GGTGCTTGGGGTTCAACCTCTCCTACTTGCAGATTTTAGGAGAGCCAATGCAGAATCGGAAGCTGGTGGCAGAGATCAGCCTGGTGAACCCTCTCACAGTGCCCCTGAACG
GCTGCGTGTTCGTGGTGGAGGGCACCGGCCTCACCGAGGGGCAGCTGGTCAAGGAGCTGTAAGAGCTTTTCCCTCTTCACCGTGCTGGGATGTTGGGAGTGGTGTGGGGGT
GGATTTCTGTGTGTGGAGCCCCCAGGGAGGGCAGTGATGGGGCACAGCAGGAGCCAGGGCTCTGCTGGTCCCTGTGGACTCAGAGTATCCCTACCCTGCACCTTGCTGGGG
AAGGGGTTTGGCCACCCTACAGAGTCTTTGCTTGCCAAAGTCCCAGCACTGTCCTGCAGCACCACGGGCCCTGGGAGGGTGACAAGGGGCTCTGAGGATGACAGGCCACAA
TGACTCCCTGCCCTCCCACAGAGAGGAGCCGGTGGAGCCGCAGGCGGAGGCCAAATTCCGGATGGATCTGATGCCGCGGCTGACGGGGCTACAGAAGCTGATGGTGGACTT
TGAGAGTGACCATCTGACGGGGGTCAAGGGCTACCGCAACGTCATCATCGCACCCCAGCCCATGTGAGCAGCTGCCAGGACCCCTCAGCTGCAGAACCCTGCCTCGCTGGC
CCCACCCGCTTTAGCCCACGATGGTCCCAGAGGGGCGACTGCCAGAGCCCGGCCTGACCCCCACACGGCGGCAGCGATGTCAGCCACGCTCCTCCCCGGGGCACAGGCGGG
ATAAAGGGGCTGGAGAGGTCTGGGGGCTGCTGTGGCAGTCACTCCCTTCACTGCTGTTCTCCCAGTCTGTGGAGTGGAAACATCCATGTGCTGTCTGTCCCCCAGCACTGG
GCACACCGGGACTGCCGTGCCAGCCCCTCTTTGCAGTGCTGGCAGCCCCAGCCCACAGAGCCATTTCCATAGAGGCAGCAGCACAACAACCCCTCCAGCCCCTGCCATCAT
CATCCTCTGACTGCAGCTCCATAGAGAGAGATTTTATACTGTGAGCACAAAGCTGTTTATGCACCATATACCTATAAAACAAATCTATAAATACTTACTGCAAATAACCTG
CTTGGGTAGAGCCAGGCATTGCCTCAATAACCATCCTAGCACTGCAGGCACTGCAGGGAGGGATGGGACATACCTTGATATGAATCAATTACACAAACGCTGAATAAAAAC
TCTATTTTTAACTTTAAAA

J

Dot plot: Zebrafinch *TGM2* vs. zebrafinch *TGM2*

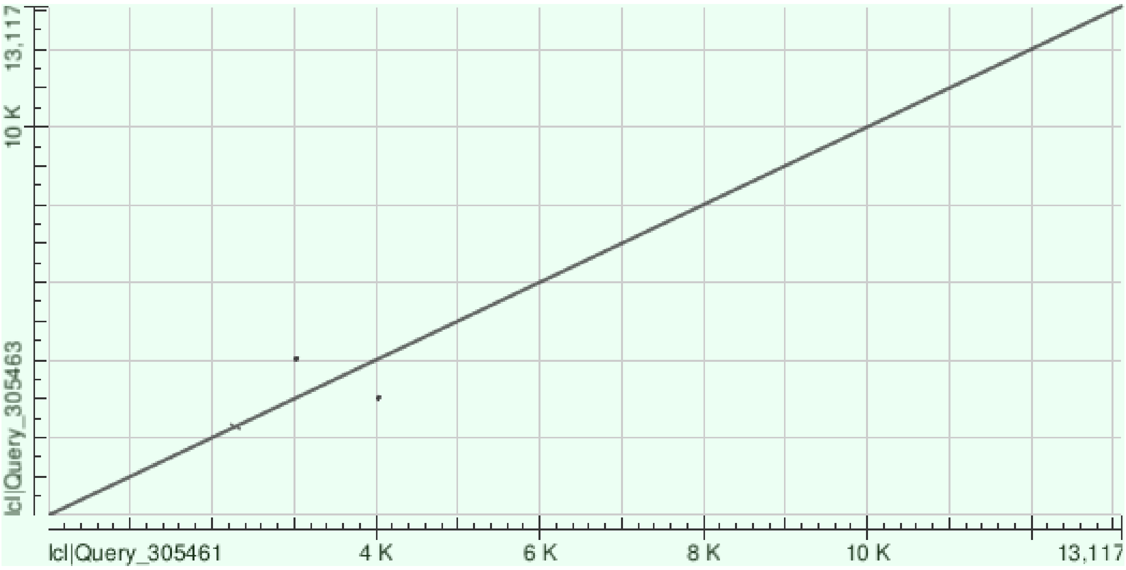

K

Human *TGM1* nucleotide sequence

>NC_000014.9:c24263177-24249114 Homo sapiens chromosome 14, GRCh38.p13 Primary Assembly

```
ATCCAGTCTGTGGGTCCTGTCCCATCCATCCTGACCTGTTCCATCTCAGCCCCAGGACTCAGTACTGCGGTTGCCAACACTGCTGCCAGGTGAGGGGCTCCCACGGGTACT
GTGGTGCCGAGTCCAGGCGGCCCACACTATCAGAGGCCGTGCCTGGATCCAGCAAGGTGGGGTGTGGGCCAGCTGTGTACCTGTCAGCCCCAGCTAGGCTGTTCCCAACAC
CAAGACTCTGCTTTCCCTGTGCACAGGCTCCGGGCACCTGCCATGCCCTACCCCTCCTGGCAGCCCCAAGTGGGGTCTTCCCTGACTTGGGAATGCCAAGGACCACAGGCC
CCGGGGTCACTTGTCTGTCTTGTGAGGAACCTTGAGTTGGGGGATTTCTGCTAAGAAATGAGTTCTAGAAGCTGTCAGTGTTGTGCACCTCTAGACTGCAGAGCTAGCAGG
TGGACGGACCAGGCCCAGGGATGCTGGAGCACTCTGATGTGTGTGCAGCTGGGTCTTGAGGCTGGGACAAGTGTCCATGCAGGGAACTATGTGGATTTCCTGGGATGGATC
GTTGAGTGGGTTTCTTCATTGGGCAGTTTTTCGGATGTTGTTTGGTGGGGGTGAGGGGAAGGCTTTTCTCTGCAGCATGAGAAGCTTCTCTGGGTGAGTCTGAATGGTCTT
CGCGGAAGGTCTCTGGATGTGTCTGGAGAATCTCTGGGCCAAGGTGGGATTGTTTCGGTCATCGGGTGGGACTGAATCAGCTGTCTGGATGGAGGGTTTCTGGGTCAACTG
GCTGGGACTACCTGGGTTAAGGAGCCACCCTGCCTCTTCCTAACAGGCATGATGGATGGGCCACGTTCCGATGTGGGCCGTTGGGGTGGCAACCCCTTGCAGCCCCCTACC
ACGCCATCTCCAGAGCCAGAGCCAGAGCCAGACGGACGCTCTCGCAGAGGAGGAGGCCGTTCCTTCTGGGCTCGCTGCTGTGGCTGCTGTTCATGCCGAAATGCGGCAGAT
GACGACTGGGGACCTGAACCCTCTGACTCCAGGGGTCGAGGGTCCAGCTCTGGCACTCGAAGACCTGGCTCCCGGGGCTCAGACTCCCGCCGGCCTGTATCCCGGGGCAGC
GGTGTCAATGCAGCTGGAGATGGCACCATCCGAGGTGAGGCCGGTCTGTCCTCCTCAGCTGAGAGCTAAAAGGCTTTAATGGGACCAGAGACTCAGCCTGGCATTTCTGGG
GGGGATAGGAAGCTAATGATAAGGGCCTGGGCTCCTCATGGGGCTTCACTGACACGGAGCTGTGTCCTTGCAGAGGGCATGCTAGTAGTGAACGGTGTGGACTTGCTGAGC
TCGCGCTCGGACCAGAACCGCCGAGAGCACCACACAGACGAGTATGAGTACGACGAGCTGATAGTGCGCCGCGGGCAGCCTTTCCATATGCTCCTCCTCCTGTCCCGGACC
TATGAATCCTCTGATCGCATCACCCTTGAGTTACTCATCGGTCAGTGGGGCTTGGATTGGGACGGGTGAAGGCCCTATGTTTGGTGGGGAGAGGCTGGCATTAGGGTCAGA
GAGGTCTTTAGGCTCCTCCCTGCCCCACTCCTCACCTCTGCCAGATAAGGCAGGTGCAAGTGTGTGGGTGTGCCCCTGGGGTTGTGTCTCAGAGAGGGTCTTGGTGTTGCA
GTTTTTCCCAAGCCCATCCTCCTCAAGCCTCCTGGGGCATCGGTCTCAGAGTACTCCTCTTCAGGGCCTGCCTCGGTGCCTCCTTCGGCATTGCTGCTCTGTCCTTTGTGC
ATCTGTTCTCATTCCCCAGGATGTGGCTGGTTCTCTCTTCTATCTCTCTCTCTCCCTCTTGCCTTCCATGTCCCCCATCTCTGCCAATTCTGTCTCTCTCTGTCTCTGTCC
CTCTGTCCCCATGACCCAAGGCAGGTGCCTCCCTTACCGCGTTGCTTGGTGTAGCTTGACTTCTCAGGCTGGTGATTATGCTGCTGCCTGGGGCAGGTGACTCCTTTTACC
CTTTGATTAGGGCCTAACCTTGCCCACCTCATCCTTTAATTTCCCAGGAACCCCTACCACCTCCACCAGGGATGCCCTGGGCTCTGGAAGCTGCAGCCCCAGGAAGCAGGC
AGGGTTGGCCTTCAGGATCAGGACTGCCCTTTCATACGGGGCAGGTGGATCTGCCCCTCCAGCCCAGGCACTGTGATAGTCAGGCAGGCAGAAGGTGACAGGAGCCCTCGT
CTCTCCTCCCCACCTCAGAGTTCAGTGTCAGGGGAACAGGTTTGGGATGTTGGACTGCGGCACTGCCCTAACATGTGGGCAAGCATTGGGTGGAGACAGAGCTGCTCTAAG
GGAGACTGTCAGAGAATAAGGTGATGGCCCAACGTAGGGACATACACAGTGGCTCATACACATTGTGGGTGGGGCCGGGAGAAGTCCCAGGCTCCATCCCCTCTCCTCAGG
GAGGCCTTGCCAGCTAATTGCTGATTTCCTACTCTAGGAAACAACCCCGAGGTGGGCAAGGGCACGCACGTGATCATCCCAGTGGGCAAGGGGGGCAGTGGAGGCTGGAAA
GCCCAGGTGGTCAAGGCCAGTGGGCAGAATCTGAACCTGCGGGTCCACACTTCCCCCAACGCCATCATCGGCAAGTTTCAGTTCACAGTCCGCACACAATCAGACGCTGGG
GAGTTCCAGTTGCCCTTTGACCCCCGCAATGAGATCTACATCCTCTTCAACCCCTGGTGCCCAGGTGAGCAGCTGGGGTCTGGAGCAGAGGGTAGATGGGAGGGAAAGACA
GGGAAGGGCTTCCTGCCCTCGGATGTGGCGGGGTGCCTAGGTGCCTGACCCCAGGAGAAGCCAGCCTCCCATTGGGCAGATGAGAGGACCGAGGCCTGGTGCAGAAAGGTG
GCCAGCCCAGGGTCACACAGCCAGCCACACCTCTCCTGGCAAAGGCCCCTGGCTACAGCCAATCTCCTCCACCATCTGTCATGCTCCCCGTGTCCTCCCACAGCAGTCCTG
TCAGGCTAGGGGCATCCCCGCATTTTCTTCTGTGGACATGAGTCCTGCAGAACTGGCCAGAAGTAGGTGAGGCCCAGGGAGAGAACTGGAGGGAACCAGTTTTGTGTGTTC
TGGGGACACAGAGGACATTGTGTACGTGGACCATGAGGATTGGCGGCAGGAGTATGTTCTTAATGAGTCTGGGAGAATTTACTACGGGACCGAAGCACAGATTGGTGAGCG
GACCTGGAACTACGGCCAGGTATGGTGCGGCTGGCCAGGGTCACATACACCCAGAGGAGCTGGGGTGGGTGTGGGGGCTGGGGTATTGGAGCAGGGTGCTGGCCTAGGGTT
CAGGCTGTCACCTCTGCCCTCCATCCCTCCTTCCAGTTTGACCACGGGGTGCTGGATGCCTGCTTATACATCCTGGACCGGCGGGGGATGCCATATGGAGGCCGTGGAGAC
CCAGTCAATGTCTCCCGGGTCATCTCTGCCATGGTGAGCACCCTCACATCTCTGAGTCCTACTGTGTGTGTGCCTGCTGCCTCGCCACACCCCCACCCTCCTCCTGCCCTT
TCTGGATTCCTCAAGAACCCCCAGCCCTGTCCCTGCCCTCGTAAAGGATACTTCAGGAAGGGGTCTCCCTCCTCTTCTCTCCCTTGCCTGCCCCCCACCCCGCCTTTCCTG
GCTCCTGTCCCAGCTCCTGTGGAATGTAGGGCCAAACCCAAAACTTGGCACACCCAGGATTCACATTCCTGTGTCAGAGCCCGGGTGGGAGGGGGGCAGATGGCTGAGGGA
AGGGCTGGGTGGAGCTCAGGCTGGTGTCTGGATCCTGAGGCATTTAGGGGTAAGGGGTGGTTGCAGGATCGGGGCCCGGCTGGTCCCCATGGACCACATGGCAGGTCCTGA
GCCCCTCCTCCACCTCCGCCCTATCTCATCCTGTCCTGGGCAGGTGAACTCCCTGGATGACAATGGAGTCCTGATTGGGAACTGGTCTGGTGATTACTCCCGAGGCACCAA
CCCATCAGCGTGGGTGGGCAGCGTGGAGATCCTGCTTAGCTACCTACGCACGGGATATTCCGTCCCCTATGGCCAGTGCTGGGTCTTTGCTGGCGTGACCACCACAGGTAG
TGGAGGGACTGGGACAGGAGTGGCCCGGGCCCTACAGGGAGAAGGGAGGAAAGCCAGGCTTACCCAGCCCTGCCCAGATGTGGCTACAGTGCAGGGTGCAGGCGGGGGTGG
GTAATAGGCTGGCCTTAATTATCATTAATTAGTGTTAACAACAGAGAGAGGCCTAAGGGAAAGGAGGGAGAAGGGACAGGCAAGGACTGAGCCAGGCAGGTGGCCTAGCTT
CCCCTGACCCCAAGTGCTCTGTAGCCCAAAGGCCTTGTGGCCCCCGTGGCCCTGACCCTGGCAGTCTATGCCCAGCTTGGCAATCCTAGTTGCCCTTCTCCCCCTGATACT
CCTGACACGATGCCTCACTTGCCCCCAACCCATGCCTTGAACCCCAACCTTCGCTCCTCCACAGTGCTGCGCTGCCTGGGTCTGGCCACCCGTACTGTCACCAACTTCAAC
TCCGCCCACGACACAGACACATCCCTTACCATGGACATCTACTTCGACGAGAACATGAAGCCCCTGGAGCACCTGAACCATGATTCTGTCTGGTGAGCTGGGGTGGACTGC
CCATCTGTGCTGAAGAATGGTGACCTGAGCTTTGGAGGTGGAGGGTGGGCAGGGCTGGGGAGGCTGGGGAAACTGAGGACTCCTGACCCTGGGGCTCAGACCCAGCCCCTC
CTCCATGTCCATCCAGGAACTTCCATGTGTGGAACGACTGCTGGATGAAGAGGCCGGATCTGCCCTCGGGCTTTGATGGGTGGCAGGTGGTGGATGCCACACCCCAAGAGA
CTAGCAGTGGTAAGCTGGGCCCCCCACTCCCCACCATGCCCCTGCTTATCTCCCCCACCTGATTAACACAGTCCAGAGTGTCAGTGCTGGGCCGGGCCGGAGAGCCAAGCC
ATGTAACCCCGCGGCTTCTTAAACGGGTTTGTCAGCTGAACCACCACGTGGATGTTAGTTTATTTTTACCATTCTATATAACTCTCTATTTGCCTACATTTTACTTATTTA
TAATGGTTTATTTTATTTATAATGGTTAATATACAAGGGAGGTTCAAAATTCAAAAGCTGGAAGAGTATTCAGAGAAAGAATTCATATTCACTATTCTTACTTGCTTTTTA
ACTTACATATCCTGGAGATAATTAACTGAAGGACCATTTTAAAGTCATTTTTATAAGTAATACATGCTTGGAAGGAAAAAAAAAGTATAGAATTGAACAACACAAAATGTA
AAGCTTCCCTATTCTCTCTCAGGAGGTATTTGGGTTTTTTTTCAAAAATATTTTTAAAGTAAATTTCTAGGTTCTACTCCAAACCTGATACATTAGACTTTCCCAGGATAG
ATGCTAACAATGTATATTTTTGAAAGTTCTTCTGGTGATTCTGACGATCAGCCGGATTTTTTGACCCACTCCAAAGGGTGGCCAGAGAGATGGAGTGACCTCCTCTGGGGC
ACAGAGTGGTAGGTCAGTGACAGAGCAAGTCCAGTCCTTTTAAGCACATACTCTTGCTTTCAACGAGTTCCTTCCTGTCTGTTCCATTGCTCACTGTGAGTTATATATACA
GCATAGAAAAAAATCAGGGCTCAGATAGGTTCAAAGAAGAAAATAGCCCTTATGACCCTGCCTTAATATATAATTACTGTTAATATAACCAACGGGTCTCTTTTCTGTGTC
CACATACATGTGTTTCTGCAGAAATTAGAACTACCATACACTCTGTTCTCAAACAAACCCCCACCCCTTCTTACCGTGATACCCCAGCCCCAGGCCACCAGGCCTGCCCTG
CCAGCTACCTTCTGTCCAGCCTCTCCCTTACCCCAGGTCAGAGAGTCCTCCCTAGCCTACATTCCTCCCCTGGCCTCTTCCCAGTGCCCTCCCTCTCACTCAGATATGTGA
ACACAGCCCCATGAGCTGTGCAACCCGGTGATGCCTGTGATGCTGAAACAGCCTGTCCCAGACGCTGGCAGATCTGAGCTCTGGCCCCCAACTCTGGGAAGTGGTGGGGTG
GACATCAGGCAGGGGGCTCAGGGCCAGCCAGACTGGGGCTTGATCCCAGCCTGATCACTTCTCAGATGTATGCTCTTGGGCAAGCACCTCGAACTTTTAGGCTTTGGTGGT
TTGGATGTAATTTGGGGTAATAGATACCATATATGGTGATTGGGAGGATTAAATGAGAAATTCTGTATCTGAAGGGACCAGCAGCATGCCGGGAGTGTAGCAGCTACTCAA
AAAGTCTGAGCTCCTTTCATTCTCCCTGCACCACCTGTCTTGTGAGATCCTGGCATGAACTTCACCTTGCTGTTTCCCCGTCTGTAAAGGAGTGTGTAGTGTTATGTCTTG
TCTAAGATTCTTTCCGGTTGGATGGTACTCACTTACCACTCTGTCCTCTCTTGCCAGGCCTCTTCCTGTCAGCCTATCTCTTCTAGACAGCCTCCTGAGTATGATAAGGGC
AGACTCTATTTCCCACCAGCTCCCCCCAGTAGAAAGGGCAGCAGGCTGCAGAGCTGAGGGTTGTTCCGCATCTCTCTGGAATGGTGGTGGCGGTGGTGTCCTCGCTCTCCC
AGCCAGCAGGCAGGGCCACTGTGGACTCTGGGTTGGTTATGATAAAGGATGGGGCGTCCTGTCCTCTGGGGGTCACAGATGGTGGACATCCAGTGTCAGCCAGCCATGAAA
GAGTATGTCAGGGACTTGGTTATCAGGCAGGCTGGTCCGCACATCTGTACCCTGCCCCCATCCTCCAGCAGAGCAGCCTGGGCTGAGGAAAGCAGATTGAGAGCCAAATGG
AGCCGCTCTCTCTTTGTTTTCCATACCTGTGTTGCACTGGCTGTCAACTGACCCCCAAGTGTTTGGGTCAGGGCTGATGACCTTGTTCCAGAGGCCACCAGGCAGTGGGAC
CGGGCCTGAGTGGGTGGGGCTGGGGCCCTGTGTGGACCTTACCCAGGGTCTGAAGAGCCCTCTCCGCCTTCTCAGATCTCTTGCCTCTGTCCCGCCTCACTCTCTAGGCAT
CTTCTGCTGCGGCCCCTGCTCTGTGGAGTCCATCAAGAATGGCCTGGTCTACATGAAGTACGACACGCCTTTCATTTTTGCTGAGGTGAGGGCTGGGCTCCAGGTGCCTTC
TTGGGCTTCAGTCATGGGTTCTCTGGTCCCAACTTCACCGCTGACTGACCAGCTCTGCGACCCGGGGCAAGTCACTCATTATACAAGTCAGTTTCCTCACCTGTAAAAGGG
GATAATCAAGGCTGCCTTGCTTCCTATACTCGGGATCAGAAGAGATAACGGGTATGAAACTGCTTCACTAGTCGTAAAGTCAGCCATTGAGTGTGAGGGATTACTGATATT
GCCAAAGTGCTCTGCCAGGAGCCTCCCAGACCAGCTTCTTCCCTGCACTCTGGCCTCGTCTGGCTCGTCTTGGAAAGTCTCCCCTTGGGCTCTGCCAGGAAGCTCCCACCC
TTGGGACCAAGCCCTGTCCCATAGCTCCTGGGATCAGGGGAAGGAGGCCGGGAGTCAGGCCACCCTCAGACCCTCTGGCTCACTCATTCCTGCTCACCCCCACCCCCCACA
GGTGAATAGTGACAAGGTGTACTGGCAGCGGCAGGATGATGGCAGCTTCAAGATTGTTTATGTGGAGGAGAAGGCCATCGGCACACTCATTGTCACAAAGGCCATCAGCTC
CAACATGCGGGAGGACATCACCTACCTCTATAAGCACCCAGAAGGTACCATTCCCCCAACCCAGCCAGCTCTGGGCCCCACAACAAGTGTTCCTGCCAAGTGCTTGGCCAC
CCACGTGGGAATTGGAACCTCACCCTTGACCCTCAACCCCCAGGCTCAGACGCAGAGCGGAAGGCAGTAGAGACAGCAGCAGCCCACGGCAGCAAACCCAATGTGTATGCC
AACCGGGGCTCAGCGGAGGATGTGGCCATGCAGGTGGAGGCACAGGACGCGGTGATGGGGCAGGATCTGATGGTCTCTGTGATGCTGATCAATCACAGCAGCAGCCGCCGC
ACAGTGAAACTGCACCTCTACCTCTCAGTCACTTTCTATACTGGTGTCAGTGGTACCATCTTCAAGGAGACCAAGAAGGAAGTGGAGCTGGCACCAGGGGCCTGTAAGTGC
```

TCCTTACCCAGCCCTGCCCCCTGGATGGCTGGGCACCTCAGGGCTGTGGACATGGAGACCCCAGGAGCAGTGGGGAGTCCCTGGGGGAAGCCTCATGTAGGGAAGCAGGCC
TCAGTGACGCCATGCCTCTTCTCCACAGCGGACCGTGTGACCATGCCAGTGGCCTACAAGGAATACCGGCCCCATCTTGTGGACCAGGGGGCCATGCTGCTCAATGTCTCA
GGCCACGTCAAGGAGAGCGGGCAGGTGCTGGCCAAGCAGCACACCTTCCGTCTGCGCACCCCAGACCTCTCCCTCACGGTGAATGCAGTTTGCTGGGGCATGAGGGGTGGT
CAGAGAGTGAAGGCCAGGGGATAAGGACATCAGAGGTGGGCGCTAAGCCAGCAGCAGGCTGGCCTGATGAGCCTTTGTAGGAGAAGGTCAAGGACCGGCTCTGGGGCCTCA
GTTTCCTCATCTGTAGCATGGGATCTTATGTCTCTGGTTCTGTGACAGCCTTTATTGAGGAATAGGTTGGAGTTTCTTCTCTGACTTCTGGGGAATCTTCTCTCAGGAGGA
TAAATGTGGGACTCGGGGAGGTTTTGCTTCTAAGCAGCTTCCCCTCTCTGGTGCAGTGTACGGTCCCCGTGTGTTTGGCCAGGGTCTCTGACGTGGGGGATGGGCCTCTTT
TTCTCTCAGTTACTGGGAGCAGCAGTGGTTGGCCAGGAGTGTGAAGTACAGATTGTCTTCAAGAACCCCCTTCCCGTCACCCTCACCAATGTCGTCTTCCGGCTCGAAGGC
TCTGGGTTACAGAGGCCCAAGATCCTCAACGTTGGGTGAGTCTGGCCTCTCCCCCTACCCCTGCTTCCACTCTGGCCTTCCCCACAGGCTGCCCTGGCTCCCAGCTTTGCT
CCCTCTCTGTCTGGACAAGTATGTTCTGTCAGGACCCAACCAAGCAGGTTGGGGCCAGCTAGCAATACCTCCCGGCCCAGCTCAGTCCCACCAGCCTTTAGGGAGACCTCT
TGGGCACCTGGTGCTATGCCATGTCCTTGCCCTGCCTCACTATCTAATTAGGGAGCAGAAGGCCCACATAGGAATCATTAGTAACTCATTGCTAATATTTGCCCAGCACTG
AAGAATTGACCAGGCCCTTTCAGGAATATTATCTCCTTTGGACACAAAAACTCTAATTTAAGTACATTAGAAGGCCAGGTGTGGTGGCTCACACCTGTAATCCCAGCACTT
TGGCAGGCCAAGGTGAGTGGATCACTTGAGCCCAGGAGTTGGAGACCAGCCTGGGCAACATGATGAAACCCTGTTTCTACAAAAAATTTTTAAAAAATTAACCAGGTTTGG
TGCTGTGCGCCTGTTGTCCCAGCTACTTGGGAGTCTAAGGTGGGAGGATCACCTGAGCCTGGAGAGGTTGAGGCTGAAGTGAGCCATGTTTGTGCCACTGCACTCCAGCCT
GGGCGACAGAGTGAGACCCCGTGTGTCTCTCTCTCTCTCTGTCACACACACACACAGACACACACACACACACAGAGACACACACACACAAGCGAAGTTTTGGAATCCTAA
TGATACGGGTTCAGCTCTAGCACTTACTGGCTATATGCCCTTGGGCAAGCGACTTCTCTCCTTCAAGCCTCAGTGTTCTCACCTGTAAAGCAGGGATAGCAATAGCCCCTG
GACCTAGGGTTGTTTAGTGCACTGCTTGGCACAAAGTTAGTGCTCCATTGGTAGTTGTTATTTTTATTATTGCCATTATTACCCCCACTTACAGATGGGGAAGCTGGCGGT
CACATCTCATATGGTCTCACAGCTCGTAGGGGGCAAACTCTGCTCTTCCCACTGTGGTGCATACCATGCACAGGCTCTCAAACTCATTGTTTGTTTTGTGCCATGAGGGCC
AAGTAGCAGTTCCAGAGGAGAGAACTCCATCCCTCAGGCCCTTACCCAGCCCGTCTGGGCCTGATCCTGGGCTGGGCAAGCCCCTGGGTGTAGTGAGGGCACACACATGCT
GTGTTCCTGGGCCTGCGGCTGTCATCTTTGATACAGACTTACACGTGCTGCCCCGTCCCCGCCTAGGAAAAGCACGGAGGGAAAGCAGAGCAGGCCAGAGCCTGGCCTTCT
AGATCAGCCTTCTGGGACAGGGGCAGGCTTCATCTTCAGACAGCGAGTTCCTGAGAAAGAACAATGGCTTGGGGTGGGGAAACTGAGGGGAGGAAGCAGAGGACCCCCTGC
CCACTTGCCCACCCCTCCCTCACACACAGCAGCTCTCCTGGTGGTGGCTGCCACCTTCGCCATTAGGCCAAAGCTGAGAAGGAAGCTATGTTGATCTTTGCCCCAGCCTGG
GCCCCTGGGAATGCTATGATGTGTCACAAAGTAGGGCTTTCCTCATGTCACCATACCATGGTCTCTGGCAGCTCAGTGAACAGAGCCTGAACCCCCCGGGTAGCCTGCTCT
GCTAGTCTTGCCCAGCAGGCATCTCTTACCCACACCCCCACCATGGTGGGGGGAGGTTCCTCGTCCTAAGGGATCCGCAGAGCTGGACTTGCCTCAGCAGCCCCACCAGGC
CTTTGCCTCTTGGCCCTGCCTGGAAGTAAGGAAAGGGCCACTGTGCTGAGTGTTGGGGGACCTGTTTCCTGTCTTTAGCTGTAGGGTTTATGTGAGTCTCCCTACAGTAAA
AGACAGGGTTGGATGATGGTTTCTTAAGGCAGATCCTCCCTGGACAGTCTGTGGCTCCTTAGCTGCTGTGAGCCCAGACCAGAGCCTCCAGTACAATTGGGAGCCTCCAGG
GGGACATCTCTGAGCTGTTGGTTCAGGTACCTAAGATTCCAAGATGGGTGTACTTTGCCTCTCATTCGCACATCAGAGGCTGTGGCATGGAACCCCATTATAGCATAAGGT
GTGCCACCCCCCTGTGGTGACTGGCCACTTAATAAACATTCCAGAACTCACTCCTCCAGATGGCTGTTGTCACCACCAAAAGCAGTCACCAGAGAGGCTGGAAGTCTGTTC
CAAACCCTTCCTGGGAATTTCCCTCAGAGTCTGAATCATATTCTCTAGAATGTCCTCAGAGTGGTCAGTCTGCCTTCTTAGGGGTAGATTTGACTTTGCAGAGAAACCTGA
AGTCATCAAGAGCTAAGACTAATTAATAAGATAAGTGAACAAGTGGGTAACTGCCCTTTTCCATCAGAACCGAGGCATGCTGTGGAAGGAAGGACAGGCTGACCGTGGCAT
CACTGAGCACACGCCTGTGTGTATGGTTTAAATGGCTCTGAAGAAGAGTCCAGGAGGAGTTTTAAACATGTTTCAAGCCCAGGCAATACGATTGAACAAGTACACAATTGG
ACAATGCTCGTTTCGACGTCTAAGTTCTGGTGTCCTTGTTGGGGAGTTGGTTTCATGACCCACACTCAGTTCCAGGAACATCCCAGCTTTAGCAGATGGCTTGTACTCAGC
TTCAGCTCACAAAAGAGGTGTGGTAAAGTCCCCCACCTCACAATGAGACCAGGGGAGGGAGAGATCTTGACATTTCCACCTAGAAAACAGTTTCTCTTGAAAATAATCCGT
TACTGCAATGTAAAGGTTACTTTCTGAAATATTATAGTTCAATTATGGTATAGAGTAAATAGACTTCTGAGGGCTGGTCTGAGCTTGAACAACCTTGAATCTTGTATCTAT
GAAAAGTTAGCAAAAATTAAGAGTGCAAATTTGTAAGCAGTCAGGACATAGCCTAGCCATTAAGTTATAGACTTAATTTATAGACTTCCTATCATACTACGTTCTATTATA
TATCCCTGCCTCCCTCCTTCAGAGAGCTCAGAAACACCTTCAAGATAATCAGATCCCACTCCTCCTTTTACTTTCACTTCCATTTTATGACCCAGAAAGTGAGGAACAGAG
AAGTTAAATAGCTTCTCATAAGCCTCACAGCTAACAAGTGGTAGAAAAGGGAGTAGAGTGGTCCAGGGAAACCCCTCAGAACCAGGTTCCAAGCCAAATGCTTTGCCCTGA
AAAAAACTCATCCTTGGAGTTAGGGATGTTGGCTCATTCTCTTAGAGGACAATAGTTAATGCACAGGTTCTATGTCTGGAAAGGGTCCCCAGCGGCAGGCTTGGGTTGAAT
AGGGCACCAGAGGTAAGGTTTAGGCAAAGGCAGGAAGGACAAATTCCCACAGGCACGAGCAAATGCAATATAAATGCAGTCATGTTCAGTGTATAAAATGAAACGTGCAGA
TTGCTGGAGGAGCTCAGAGAAGGGTGAGTTTCCTTTTCCTAGAGGCAGGAGGTGCTCAGGGGAGCCGTCACTGAGGAAGTGGTGTGCCAGGCCTTGGAGGAAAAGTAGGCC
TTCACAAGGACACCCAGGGTGGGGAGGCAGAGCTGGGTGCTCATTCCACACAAGGCAAAGACTGCAAAGCATGACTGTGGAGGTGAGTCTGGGGAAGAAGGCATTCGGTAG
GGCTGCTCAGCAGGAGGAATGATGGGGGGTGATGAGGCTGTGCAAGTAGCAGGGACTGGGTCAGAGTGAGCCTGTGTGTCAAGTTAGAGAGCCAGGATCGTGTGCCACAAG
CACCAAAGGAGCACTAAATCCTTGAAGGATTTAGTTCACCTCTGAGTGGTTAGCAGAACAGAATACAAAAGTGGGATGCGAGGCAAGGAGTCCCGTTAGAGGACTAGTGAA
ATCATGATTCCCAGGGGAGGTCTGGGAATCAATTTTTCAGAGGCCCATGTGGAAAGGAGAAAATGAAAACACTGGTTGTGTGTGTGTGTGTGTGTTTGTCCACCCACCTCT
CTGTCTCTCTCACACACACACACACACACACACACACACACAGAGACATAAGGGTTTATGTCCATTTAAAGATGTCTGTTGTCTGTTCTTGGGAAGGATTTTCCTTTTTTT
TCAAAGATAACATCCTTCTAATGTTTTGGTGTGTGAAAATGCTCTTTTCTCTTTGGAACGACGATCATAGCAGAGAGTGATTACTTTTCTTCAACCTATGCTCTTGTCAAA
ACTGAAAGTATTTGAAAATATTGTGTTGGGCCCCCAAAGTCTCATTTTGAAGTTTTCTTGGTTTTGGAAACCCCAGGTGAGATGAGGATGTTCTGTCTGGGGCAGGAGCAG
TGGTGATGGAGGGGATGGCATGAGAGGCCTCTGCGGTGGCATCAGCAGTCTTTGTCCAGGTGGATGTGGGAGCAGGGAGGGAGGAGTCCCAGACGATGGGGAGAGTTCCAG
CACAGGCAGCTGGTGGGTGGCTGCTGAGCTGGCTATGCAGAGGCGCCCTGGGGATCCTGGTCTTGGGTGTGGAAATTTCAGGTAAGCCTGGAATTGTCTGAATGGGTCCAC
CTCCTGCTTCTTCCTCCTGACTGCCCTGGATCAGCTCTTACTCCCCACTCCACCCCCAATTACTCCAGGCCCATGACCTTGTCTCTGCCCACAGGGACATTGGAGGCAATG
AAACAGTGACACTGCGCCAGTCGTTTGTGCCTGTGCGACCAGGCCCCCGCCAGCTCATTGCCAGCTTGGACAGCCCACAGCTCTCCCAGGTGCACGGTGTCATCCAGGTGG
ATGTGGCCCCAGCCCCTGGGGATGGGGGCTTCTTCTCAGACGCTGGAGGTGACAGTCACTTAGGAGAGACCATCCCTATGGCATCTCGAGGTGGAGCTTAGCCCCGTGCCA
GGAGCAATGGGACTGGAGTCAGATGAGCAAGGACATTGCCCCAAGATAGGGGCACACTACAGAGCAGCTCCCCAGGAGCTCAGGTGGGGAGTCCAGGGCTCCCGGAGGGGG
AGTCCAGGGCTCCCGGAGAGGGAGTCAGTCTTCACTTGCACTGGGGGAACAGATGCTAATAAACTGTTTTTTAATGAA

L

Dot plot: Human *TGM1* vs. human *TGM1*

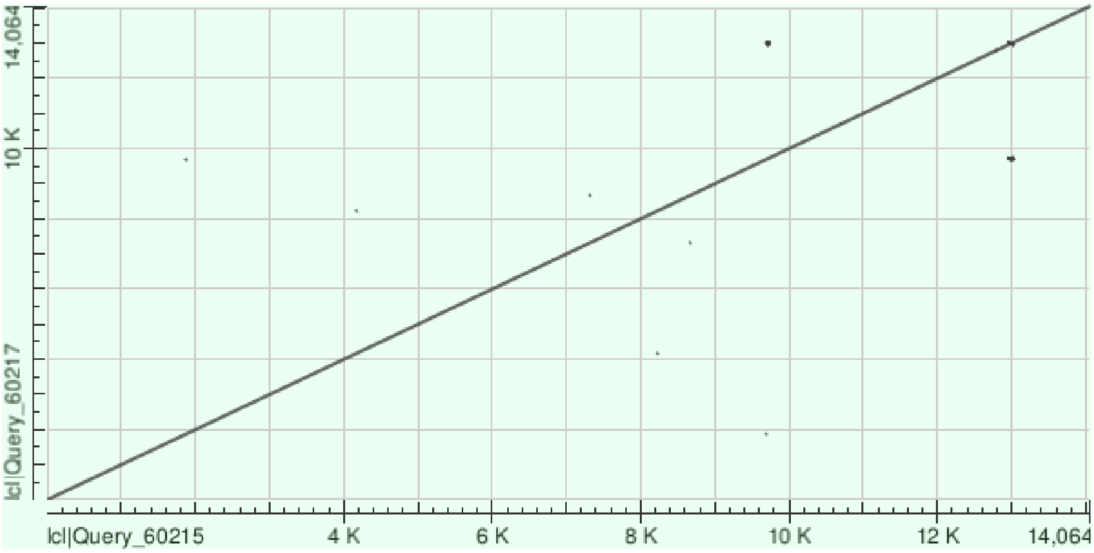

Figure S4. Dot plot analysis of chicken *TGM1* introns and zebra finch *TGM1* in comparison to zebra finch *TGM2* and human *TGM1*. To visualize sequence repeats, the nucleotide sequences of the last-but-one **(A)** and last **(B)** intron of chicken *TGM1* (accession numbers provided in the figure) were subjected to dot plot analysis using the NCBI BLASTn tool using default parameters (Word size 11; Expect value 0.05; Match/mismatch scores 2,-3, Gap costs 5,2; Filter string F; Genetic code 1; Karlin-Altschul statistics: Lambda 0.633731, 0.625; K 0.408146, 0.41; H 0.912438, 0.78). The dot matrix view shows regions of sequence similarity based upon the BLAST results. The same sequences were used as query (X-axis) and subject (Y-axis). Numbers represent nucleotides. Alignments are shown as lines. Plus strand matches are slanted from the bottom left to the upper right corner, and minus strand matches are slanted from the upper left to the lower right. The number of lines shown in the plot is identical to the number of alignments found by BLASTn. Nucleotide sequences of zebra finch TGM1 **(C)**, zebra finch TGM2 **(I)** and human TGM1 **(K)** are shown in FASTA format with color-coded nucleotides. The nucleotide sequences of zebra finch *TGM1* **(D)**, zebra finch *TGM2* **(J)** and human *TGM1* **(L)** and the sequences of zebra finch *TGM1* intron 1 **(E)**, intron 2 **(F)**, intron 3 **(G)** and intron 4 **(H)** were subjected to dot plot analysis. K, kilo (1000).
